# The Impact of COVID-19 on Mental Healthcare Utilization in Switzerland Was Strongest Among Young Females—Retrospective Study in 2018–2020

**DOI:** 10.3389/ijph.2023.1605839

**Published:** 2023-05-19

**Authors:** Yael Rachamin, Levy Jäger, Reka Schweighoffer, Andri Signorell, Caroline Bähler, Carola A. Huber, Eva Blozik, Erich Seifritz, Thomas Grischott, Oliver Senn

**Affiliations:** ^1^ Institute of Primary Care, University of Zurich and University Hospital Zurich, Zurich, Switzerland; ^2^ Campus Stiftung Lindenhof Bern (SLB), Bern, Switzerland; ^3^ Institute for Clinical Research, Department of Medicine, University of Basel, Basel, Switzerland; ^4^ Department of Health Sciences, Helsana Group, Zurich, Switzerland; ^5^ SWICA Health Services Research, Winterthur, Switzerland; ^6^ Department of Psychiatry, Psychotherapy and Psychosomatics, University Hospital of Psychiatry Zurich, Zurich, Switzerland

**Keywords:** mental health, COVID-19, health services research, psychiatric care, interrupted time series analysis, administrative data

## Abstract

**Objectives:** To provide a thorough assessment of the impact of the COVID-19 pandemic on the utilization of inpatient and outpatient mental healthcare in Switzerland.

**Methods:** Retrospective cohort study using nationwide hospital data (*n* > 8 million) and claims data from a large Swiss health insurer (*n* > 1 million) in 2018–2020. Incidence proportions of different types of psychiatric inpatient admissions, psychiatric consultations, and psychotropic medication claims were analyzed using interrupted time series models for the general population and for the vulnerable subgroup of young people.

**Results:** Inpatient psychiatric admissions in the general population decreased by 16.2% (95% confidence interval: −19.2% to −13.2%) during the first and by 3.9% (−6.7% to −0.2%) during the second pandemic shutdown, whereas outpatient mental healthcare utilization was not substantially affected. We observed distinct patterns for young people, most strikingly, an increase in mental healthcare utilization among females aged <20 years.

**Conclusion:** Mental healthcare provision for the majority of the population was largely maintained, but special attention should be paid to young people. Our findings highlight the importance of monitoring mental healthcare utilization among different populations.

## Introduction

The COVID-19 pandemic has had major disruptive effects on people’s lives globally, with potential adverse effects on their mental health. Mitigation measures such as spatial distancing can cause feelings of isolation and loneliness, and medical and financial uncertainties have contributed to increased levels of stress and anxiety [1–3]. Accordingly, many survey-based studies have reported an increase in mental health problems in different populations especially in the beginning of the pandemic [4–7]. In Switzerland, evidence suggests that the mental health status of the majority of the population has not been severely affected by the pandemic, but that young people and especially young females have been particularly vulnerable to the detrimental mental health effects of the pandemic [7, 8]. These findings are well in line with the international literature [9–14].

At the same time, the pandemic has affected the provision of mental healthcare, particularly in the beginning of the pandemic and in periods with many COVID-19 cases when healthcare services had to be reorganized in order to reduce the spread of the virus and to ensure care capacities for people with COVID-19. Accordingly, decreases in different forms of mental healthcare utilization, especially during shutdown periods, have been reported, e.g., in hospitalizations and/or emergency department visits for mental health diagnoses [15–18], presentations for or diagnoses of mental health conditions in primary care [19, 20], and general population antidepressant drug purchase [21]. However, some studies also reported increases for certain mental healthcare utilization outcomes as the pandemic progressed, e.g., increases in antidepressants fillings in the general population [22] or in mental health outpatient visits especially among children and adolescents in the summer of 2020 [9, 23].

To thoroughly understand mental health service utilization, a comprehensive examination of the various forms of mental health service utilization in different settings is needed. Here, we set out to assess the impact of the COVID-19 pandemic in 2020 on inpatient and outpatient mental healthcare utilization in Switzerland for the whole population and for the vulnerable group of young people specifically.

## Methods

### Study Design, Setting, and Data Sources

We performed a retrospective cohort study in January 2018 to December 2020. We used Swiss routine data from two sources: 1) the Medical Statistic of Hospitals of the Federal Statistical Office (“Bundesamt für Statistik, Medizinische Statistik der Krankenhäuser 2018–2020,” short MedStat [24]), and 2) the claims database of the Swiss health insurer Helsana Group. Ethics committee approval was not required because all data were retrospectively collected and anonymized (Federal Act on Research involving Human Beings, Art. 2).

Inpatient admission data was retrieved from the MedStat, an official database which collects inpatient data of all Swiss hospitals for the purpose of epidemiological surveillance, healthcare planning, quality control, cantonal comparisons, etc. Due to incompleteness of data sets (hospitals are not required to provide detailed information on cases that span more than one calendar year), data for the first and last week of each year were omitted. For outpatient data, there is no nationwide database in Switzerland. Therefore, outpatient data for psychotherapy consultations and psychotropic medication claims was provided by Helsana Group, which is one of the largest health insurers in Switzerland and provides basic health insurance to around 15% of Swiss residents (as of January 2020) [25]. Basic health insurance is mandatory in Switzerland for every person even if they additionally purchase private/supplementary insurance. Health insurance companies must accept all applicants for basic insurance and the benefit package is the same among all insurance companies throughout the country. Insurance switch is possible per 1st January of each year, which leads to (minor) annual changes in the insured collectives from which the claims originate. Psychotropic drugs and psychiatric consultations are covered by basic health insurance if prescribed by physicians. It is estimated that only around 3% of claims are paid out-of-pocket [26].

### Outcomes

For each outcome, we investigated weekly incidence proportions (see analysis section below). Outcomes in the inpatient setting were:

- Psychiatric admissions: all psychiatric admissions, admissions for affective disorders, neurotic disorders, and psychotic disorders.

Psychiatric admissions were identified as admissions with the main diagnosis in the International Classification of Disease 10th Revision (ICD-10) chapter V (“Mental and behavioral disorders”). For the sake of concise terminology, we used the term *affective disorders* for admissions with the main diagnosis in ICD-10 F30-F39 (“Mood [affective] disorders”), *neurotic disorders* for ICD-10 F40-F48 (“Neurotic, stress-related and somatoform disorders”), and *psychotic disorders* for ICD-10 F20-27 (“Schizophrenia, schizotypal and delusional disorders”).

Outcomes in the outpatient setting were:

- Psychotherapy consultations: all psychotherapy consultations, and specific groups, namely face-to-face vs. teleconsultations, first vs. further consultations.
- Psychotropic medication claims: all psychotropic medications, and specific groups, namely antidepressants, anxiolytics, and antipsychotics.

Psychotherapy consultations were defined according to TARMED (Swiss fee for-service tariff system) positions (Supplementary File S1). Psychotherapy consultations included both consultations with psychiatrists as well as psychologists, except for the analysis of first vs. further consultations, for which only consultations with psychiatrists were considered (because discrimination was not possible for consultations with psychologists). Medication groups were adopted from the anatomical therapeutic chemical (ATC) codes. For “all psychotropic medications”, we considered psycholeptics (N05) and psychoanaleptics (N06).

### Time Periods

Data was collected from both sources between January 2018 and December 2020, thus defining the observation period to cover a pre-pandemic period of over 2 years and the first calendar year of the COVID-19 pandemic in Switzerland up to December 2020. In contrast to other countries, Switzerland imposed no strict lockdowns, but rather a gradual introduction and relief of measures depending on current pandemic developments. A detailed overview of mitigation measures in Switzerland and a quantification of the stringency of COVID-19 policy measures are found elsewhere [27, 28]. We defined four pandemic periods in 2020 as follows:

- Pre-shutdown: calendar weeks 9–11. Time between the first confirmed COVID-19 case in Switzerland and the shutdown (see next definition); characterized by awareness of COVID-19, but before any mitigation measures were introduced.
- First shutdown: calendar weeks 12–19. Period with strict mitigation measures, including the closure of schools and non-essential businesses and, importantly, a ban on non-urgent healthcare up to week 17.
- Summer: calendar weeks 20–42. Relatively loose mitigation measures.
- Second shutdown: calendar weeks 43–52. Again, closure of non-essential businesses and restaurants, but no ban on non-urgent healthcare.

### Statistical Analysis

For each outcome, we used an uncontrolled interrupted time series (ITS) analysis approach with weekly incidence proportions per 100,000 people in the respective population as response variable. We assumed a constant population throughout the entire calendar year, disregarding births, deaths, (im)migration etc. We fitted generalized additive models (GAMs) with linear terms to account for the secular trend over the entire observation period, cyclic cubic splines with 52-week periods to account for seasonality [29], and indicator variables for different vacation periods. In addition, the models/predictors included terms of the form $\beta_{p}+\beta_{tp}\left(t-t_{p}\right)$ for each of the previously defined pandemic periods ( p=1,2,3,4 ), where $\beta_{p}$ is the level change and $\beta_{tp}$ the trend change in period p starting in week $t_{p}$ . Each of these terms was set to zero outside of the respective period. This approach corresponds to the use of segmented linear regression to assess the effect of the different pandemic periods [30, 31]. For each outcome, we conducted a subgroup analysis on the population aged ≤30 years. Within these subgroups, we used the same model structure as in the models for the overall population, but allowed for coefficients specific to the four strata specified by combinations of gender (male, female) and age decade (<20 years, 20–30 years; see Supplementary File S1 for the definition of age groups). Results of model diagnostics including residual variance structure specifications are provided in the Supplementary Files S1, S2 and regression coefficients are presented in the Supplementary File S3.

To quantify the effect of the pandemic periods, we used the ITS models to predict the weekly incidence proportions once under the true COVID-19 scenario and once under a counterfactual scenario in absence of COVID-19 (i.e., where the coefficient estimates for all $\beta_{p}$ and $\beta_{tp}$ were set to zero). For each outcome and each pandemic period p , we report an absolute effect estimate as the difference between the predictions of the two scenarios cumulated over the entire period (thus corresponding to an absolute difference of incidence proportions over the entire period). We computed 95% confidence intervals (CIs) of these effect estimates based on the estimated covariance of the estimates for $\beta_{p}$ and $\beta_{tp}$ (see Supplementary File S1). For the subgroup analyses, effect estimates were computed stratum-wise. In addition, we calculated relative effect estimates as the ratio of these absolute effect estimates to the cumulated prediction over the respective period in the counterfactual scenario (thus corresponding to a relative difference of incidence proportions over the entire period). Corresponding 95% CIs were obtained via posterior simulation conditioned on the model estimates using 500 samples [32, 33]. (Note that assessment of statistical significance by means of the 95% CIs for the absolute and the relative effect estimates may lead to slightly different results.) We further used time series charts for visualization of the observed data and the predictions from the ITS models under both the true COVID-19 and the counterfactual scenario with 95% confidence intervals derived from the models’ parametric terms.

We used R version 4.2.0 (R Foundation for Statistical Computing, Vienna, Austria) for statistical analysis and visualization [34]. GAMs allowing for stratum-specific smoothing terms and residual variance structure specification were fitted with the function gamm() of the package mgcv [32]. Figures were created with the package ggplot2 [35].

## Results

### Study Sample

The study sample included all Swiss inhabitants for the inpatient setting (data source 1) and all people insured with Helsana Group for the outpatient setting (data source 2) in the years 2018–2020. An overview of the study sample and its healthcare utilization in the different observation years is given in Table 1.

**TABLE 1:** Description of study population and mental healthcare utilization in the years 2018–2020, overall and for the subgroup of young people. Switzerland, 2018–2020.

	Year 2018		Year 2019		Year 2020	
	All	Subgroup (age ≤30 years)	All	Subgroup (age ≤30 years)	All	Subgroup (age ≤30 years)
**Inpatient**						
*n* (%)	8,369,611 (100%)	2,742,134 (100%)	8,444,967 (100%)	2,751,676 (100%)	8,511,560 (100%)	2,754,858 (100%)
Female, *n* (%)	4,230,171 (51%)	1,345,060 (49%)	4,264,964 (51%)	1,348,243 (49%)	4,296,704 (50%)	1,348,847 (49%)
Age in years, *n* (%):						
<20 years	1,546,519 (18%)	1,546,519 (56%)	1,558,207 (18%)	1,558,207 (57%)	1,571,627 (18%)	1,571,627 (57%)
20–30 years	1,195,615 (14%)	1,195,615 (44%)	1,193,469 (14%)	1,193,469 (43%)	1,183,231 (14%)	1,183,231 (43%)
30–40 years	1,189,141 (14%)	0	1,207,648 (14%)	0	1,222,901 (14%)	0
40–50 years	1,203,845 (14%)	0	1,193,982 (14%)	0	1,188,643 (14%)	0
50–60 years	1,249,309 (15%)	0	1,267,057 (15%)	0	1,278,709 (15%)	0
60–70 years	904,757 (11%)	0	916,031 (11%)	0	932,985 (11%)	0
70–80 years	671,159 (8%)	0	690,289 (8%)	0	706,158 (8%)	0
80+ years	409,266 (5%)	0	418,284 (5%)	0	427,306 (5%)	0
Psychiatric inpatient admissions, mean weekly incidence per 100,000 people (SD)						
Total	23.7 (1.3)	23.8 (1.0)	23.5 (2.2)	16.7 (1.4)	16.8 (1.1)	17.4 (2.0)
For affective disorders	7.1 (0.5)	6.9 (0.4)	6.8 (0.8)	4.0 (0.6)	4.0 (0.5)	4.3 (0.7)
For neurotic disorders	3.6 (0.3)	3.5 (0.3)	3.4 (0.4)	3.4 (0.5)	3.3 (0.5)	3.1 (0.5)
For psychotic disorders	3.4 (0.3)	3.4 (0.2)	3.5 (0.3)	2.5 (0.3)	2.5 (0.3)	2.5 (0.4)
**Outpatient**						
*n* (%)	1,087,961 (100%)	349,945 (100%)	1,146,520 (100%)	375,520 (100%)	1,262,056 (100%)	419,551 (100%)
Female, *n* (%)	565,621 (52%)	171,874 (49%)	593,282 (52%)	184,431 (49%)	648,629 (51%)	206,068 (49%)
Age in years, *n* (%):						
<20 years	199,346 (18%)	199,346 (57%)	214,832 (19%)	214,832 (57%)	240,155 (19%)	240,155 (57%)
20–30 years	150,599 (14%)	150,599 (43%)	160,688 (14%)	160,688 (43%)	179,396 (14%)	179,396 (43%)
30–40 years	129,998 (12%)	0	143,957 (13%)	0	173,116 (14%)	0
40–50 years	143,026 (13%)	0	151,748 (13%)	0	170,570 (14%)	0
50–60 years	150,121 (14%)	0	157,071 (14%)	0	170,822 (14%)	0
60–70 years	127,078 (12%)	0	127,652 (11%)	0	133,323 (11%)	0
70–80 years	110,249 (10%)	0	112,023 (10%)	0	114,610 (9%)	0
80+ years	77,544 (7%)	0	78,549 (7%)	0	80,064 (6%)	0
Outpatient psychotherapy consultations, mean weekly incidence per 100,000 people (SD)						
Total	1,602.5 (342.8)	1,598.4 (345.1)	1,593.7 (352.0)	1,436.0 (361.5)	1,447.5 (368.2)	1,435.0 (360.1)
Face to face	1,481.0 (322.4)	1,477.6 (324.0)	1,392.4 (335.1)	1,320.0 (338.8)	1,332.0 (343.4)	1,258.0 (344.4)
Telemedicine	133.8 (23.9)	132.6 (24.4)	211.8 (118.2)	132.4 (28.8)	130.4 (30.9)	189.8 (101.8)
First consultation^a^	48.3 (10.1)	48.6 (10.4)	44.7 (11.8)	48.7 (11.2)	49.5 (10.7)	45.0 (12.5)
Further consultations^a^	1,046.9 (218.7)	1,021.7 (215.5)	1,008.1 (216.8)	743.4 (181.7)	733.8 (183.7)	734.3 (179.5)
Outpatient psychotropic medication claims, mean weekly incidence per 100,000 people (SD)						
Total^b^	2,583.9 (236.0)	2,545.7 (215.4)	2,353.3 (310.2)	503.7 (70.7)	502.2 (62.9)	499.3 (84.3)
Antidepressants	911.2 (88.4)	903.9 (78.3)	838.8 (115.5)	165.2 (22.1)	166.2 (19.9)	168.5 (28.9)
Anxiolytics	557.9 (51.5)	545.4 (46.1)	498.5 (66.7)	78.2 (9.2)	76.8 (8.7)	77.1 (11.9)
Antipsychotics	524.4 (41.5)	530.3 (45.3)	492.1 (59.1)	108.2 (13.8)	104.2 (11.4)	107.8 (15.1)

^a^ Only considering consultations with psychiatrists (not psychologists; due to the tariff structure).

^b^ Considering all drugs in the ATC group N05 (“psycholeptics”) and N06 (“psychoanaleptics”).

Abbrevations: SD, standard deviation; ATC, anatomical therapeutic chemical.

### Inpatient Setting

**FIGURE 1:**
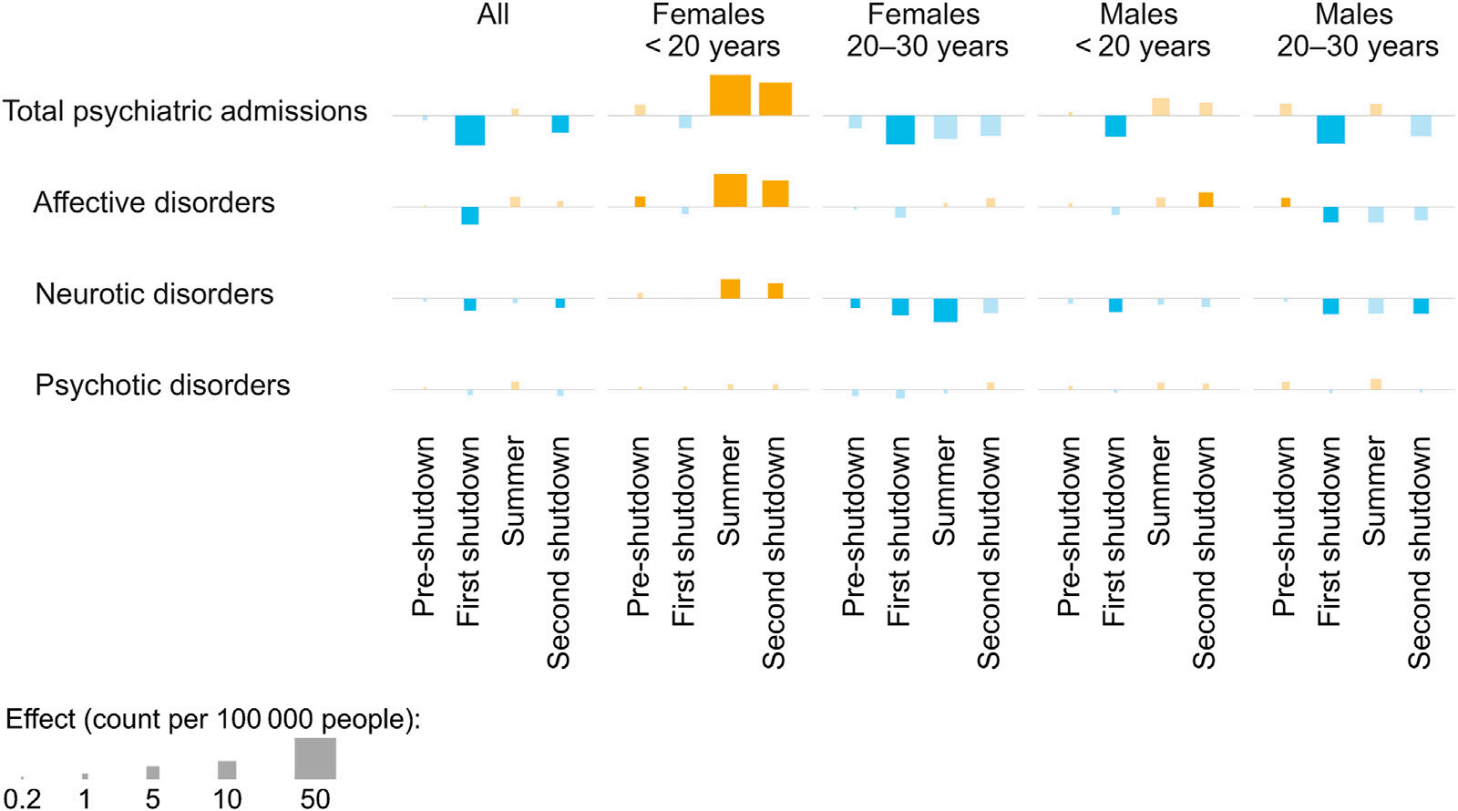
Effects of different pandemic periods on inpatient admissions in the year 2020. The squares represent the increases (orange)/decreases (blue) in incidence proportions over the respective period resulting from the pandemic scenario (vs. the pandemic-free scenario), for different outcomes. Significant effects are indicated by opaque squares (vs. transparent squares). Number of observed people in 2020, overall and per strata: n_all_ = 8,511,560, n_f,<20_ = 763,618, n_f,20–30_ = 585,229, n_m,<20_ = 808,009, n_m,20–30_ = 598,002. Switzerland, 2018–2020.

**FIGURE 2:**
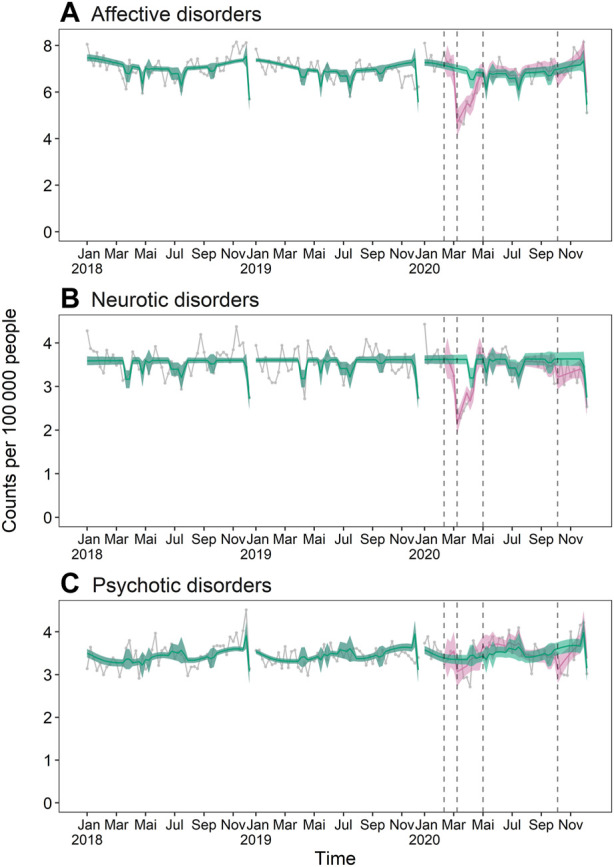
Weekly psychiatric inpatient admission incidence in the years 2018–2020: **(A)** affective disorders, **(B)** neurotic disorders, **(C)** psychotic disorders. The turquoise lines and bands represent the expected weekly incidence per 100,000 people in absence of a pandemic (pandemic-free scenario) with 95% confidence interval. The pink lines and bands represent the expected weekly incidence in consideration of the pandemic (pandemic scenario) with 95% confidence interval. The grey connected dots are observed values, and the black vertical dashed lines separate the different pandemic periods. Number of observed people per year: n_2018_ = 8,369,611, n_2019_ = 8,444,967, n_2020_ = 8,511,560. Switzerland, 2018–2020.

During the first shutdown, total psychiatric admissions were decreased by 16.2% (95% CI −19.2% to −13.2%) in the pandemic scenario compared to the pandemic free scenario (Figure 1; for an overview of all absolute and relative effects, see Supplementary File S4). A similar pattern could be observed for admissions for affective and neurotic disorders, but not psychotic disorders, which were not significantly affected (Figures 1, 2A–C). In the summer, total psychiatric admissions were not affected by the pandemic, whereas in the second shutdown, they were again decreased by 3.9% (95% CI −6.7% to −0.2%) (Figure 1).

The subgroup analyses revealed similar patterns, except in the stratum of females aged <20 years, whose total psychiatric admissions were not significantly affected by the first shutdown but exhibited pandemic-related increases from summer on (Figure 1). Total psychiatric admission incidence of females aged <20 years in the pandemic scenario exceeded numbers in the pandemic-free scenario by 17.6% (95% CI 12.2%–27.8%) in summer and by 24.4% (95% CI 14.4%–34.7%) in the second shutdown. Again, the same pattern could be observed for affective and neurotic disorders, but not for psychotic disorders (Figure 1).

### Outpatient Setting

**FIGURE 3:**
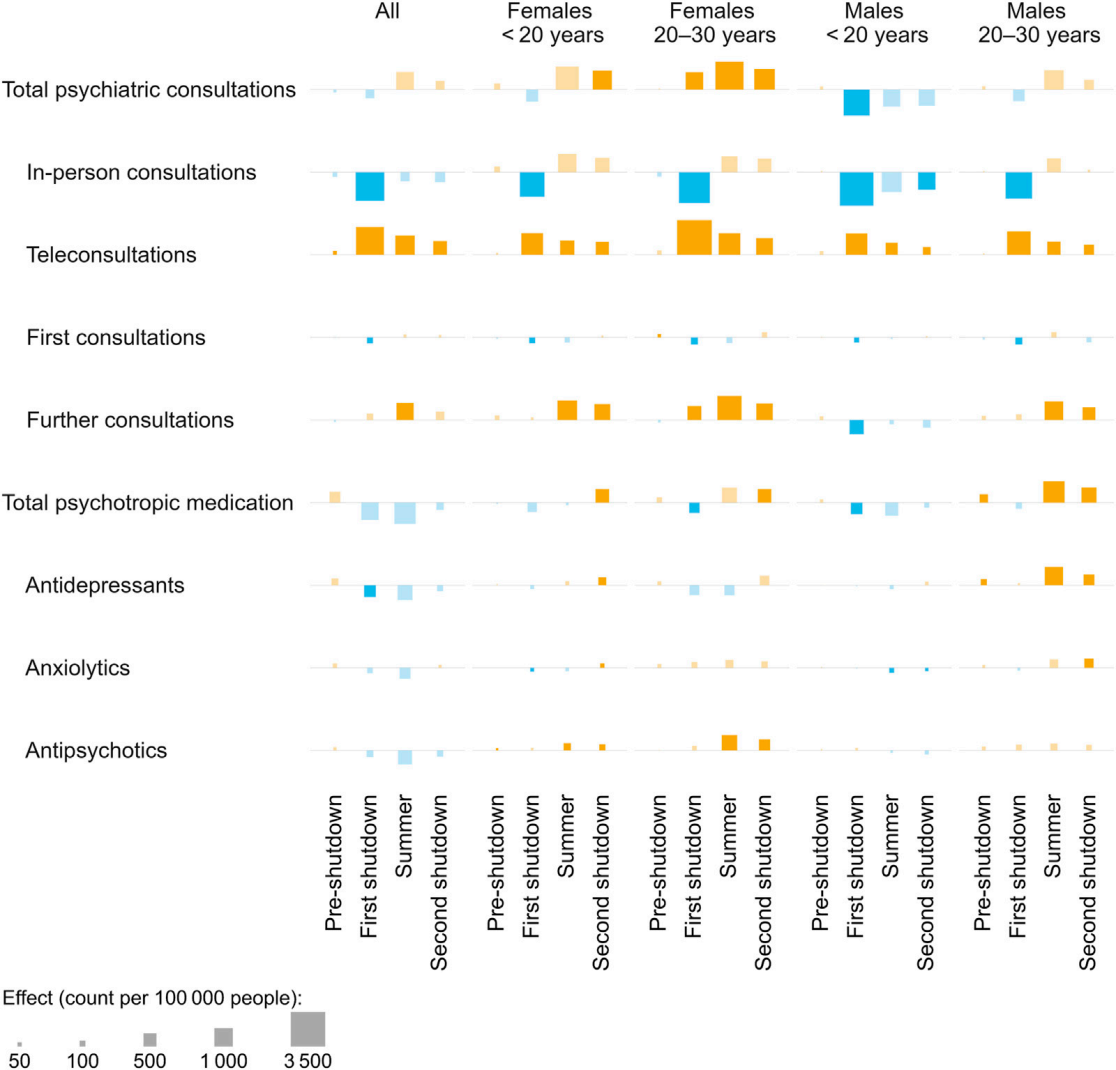
Effects of different pandemic periods on outpatient mental healthcare in the year 2020. The squares represent the increases (orange)/decreases (blue) in incidence proportions over the respective period resulting from the pandemic scenario (vs. the pandemic-free scenario), for different outcomes. Significant effects are indicated by opaque squares (vs. transparent squares). Number of observed people in 2020, overall and per strata: n_all_ = 1,262,056, n_f,<20_ = 116,754, n_f,20–30_ = 89,314, n_m,<20_ = 123,401, n_m,20–30_ = 90,082. Switzerland, 2018–2020.

**FIGURE 4:**
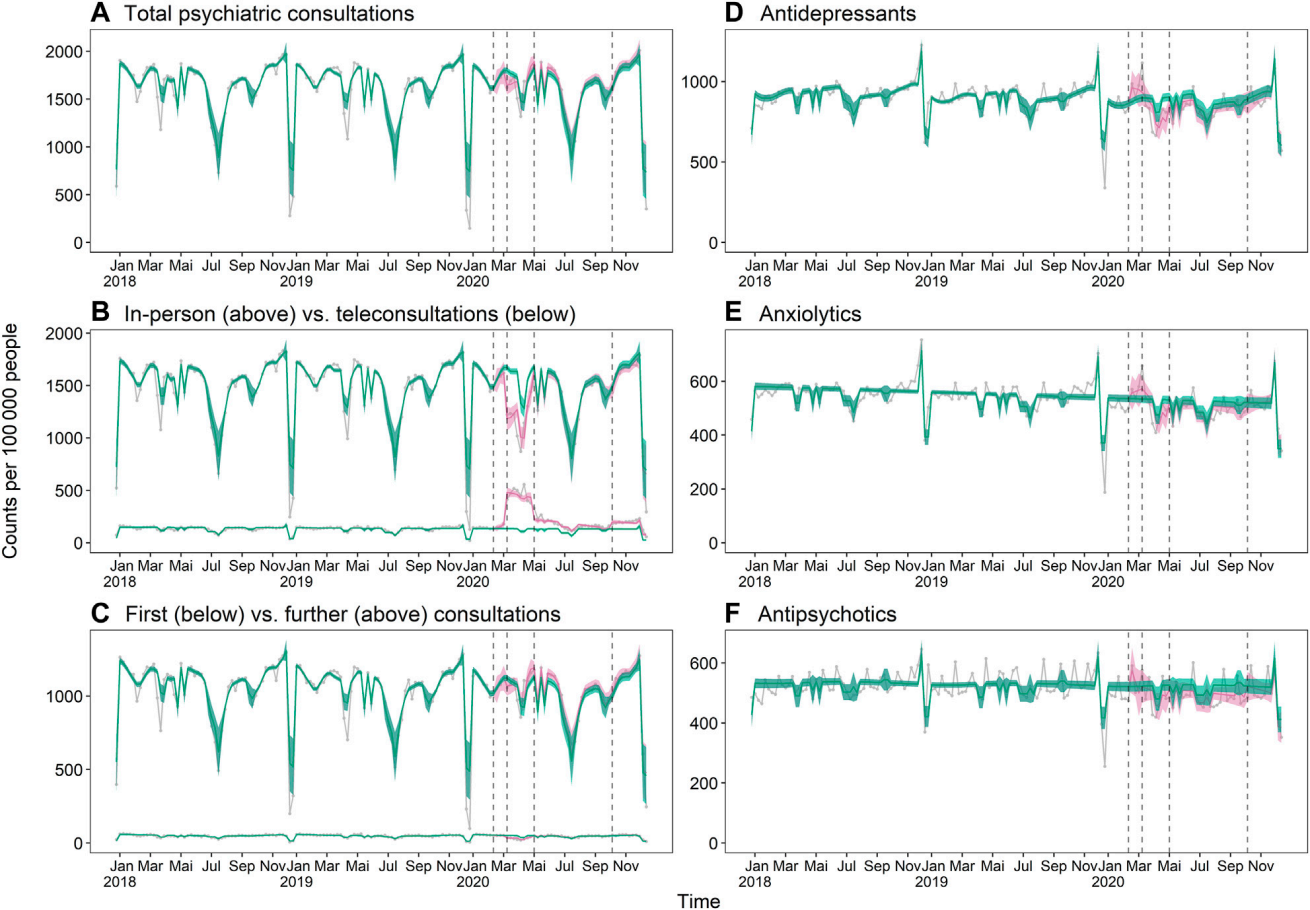
Weekly outpatient healthcare utilization incidence in the years 2018–2020: **(A)** total psychiatric consultations, **(B)** in-person vs. teleconsultations, **(C)** first vs. further consultations, **(D)** antidepressants, **(E)** anxiolytics, **(F)** antipsychotics. The turquoise lines and band represent the expected weekly incidence per 100,000 people in absence of a pandemic (pandemic-free scenario) with 95% confidence interval. The pink lines and band represent the expected weekly incidence in consideration of the pandemic (pandemic scenario) with 95% confidence interval. The grey connected dots are observed values, and the black vertical dashed lines separate the different pandemic periods. Number of observed people per year: n_2018_ = 1,087,961, n_2019_ = 1,146,520, n_2020_ = 1,262,056. Switzerland, 2018–2020.

Total outpatient psychiatric consultation incidence of the whole population was not significantly affected by any of the pandemic periods (Figures 3, 4A). However, there was a shift in the modality of these consultations during the first shutdown, with in-person consultations decreasing by 22.4% (95% CI −26.3% to −18.5%) and teleconsultations increasing by 255.4% (95% CI 226.0%–281.8%) in the pandemic scenario compared to the pandemic-free scenario. Teleconsultations remained elevated in the following period, but less pronouncedly (Figures 3, 4B). First consultations with psychiatrists were 32.6% (95% CI −40.1% to −23.7%) lower than expected during the first shutdown, whereas further consultations with psychiatrists were 4.1% (95% CI 0.6%–7.9%) higher than expected during the summer (Figures 3, 4C). Outpatient psychiatric medication claims of the whole population were not significantly affected by any of the pandemic periods (Figures 3, 4D–F).

The subgroup analysis revealed mostly similar patterns. Among all strata, there was a shift from in-person to teleconsultations, a decrease in first consultation with psychiatrists during the first shutdown and (except for males aged <20 years) an increase in further consultations starting from the first shutdown or summer (Figure 3). However, some patterns were strikingly different among different strata: males aged <20 years exhibited a decrease in total psychiatric consultations in the first shutdown (−26.1%, 95% CI −33.5% to −18.8%) whereas both female strata exhibited increases in later periods instead (females aged <20 years: 12.2%, CI 2.6%–22.4% in the second shutdown; females aged 20–30 years: 4.2%, 95% CI 1.1%–7.2% in summer and 5.4%, 95% CI 1.9%–9.3% in the second shutdown).

Psychotropic medication claims were increased in the summer and/or second shutdown in all strata except males aged <20 years. For females, this increase was particularly pronounced for antipsychotics (females aged <20 years: 35.3%, 95% CI 15.6%–58.2% in summer and 55.7%, 95% CI 33.7%–84.1% in the second shutdown; females aged 20–30 years: 22.2%, 95% CI 12.6%–32.1% in summer and 26.9%, 95% CI 14.8%–38.7% in the second shutdown), whereas for males aged 20–30 years, it was particularly pronounced for antidepressants (19.2%, 95% CI 11.6%–28.9% in summer and 14.5%, 95% CI 6.2%–23.5% in the second shutdown).

## Discussion

In this retrospective cohort study, we found that inpatient psychiatric admissions decreased during the first two pandemic shutdowns in Switzerland, whereas the incidence of outpatient mental healthcare utilization was not substantially impacted. Importantly, we observed distinct patterns for the subgroup of young people, most strikingly, an increase in both inpatient and outpatient mental healthcare utilization of females aged <20 years after the first shutdown.

We observed a decrease in inpatient but not outpatient mental healthcare utilization during the shutdowns and particularly during the first shutdown in spring 2020. The greater impact of the COVID-19 pandemic on inpatient care compared with outpatient care has also been observed in general healthcare utilization in Switzerland, e.g., in studies of all-cause hospital admissions [36] and primary care visits [37]. This difference between the inpatient and outpatient setting can plausibly be explained by the fact that part of the outpatient services could be performed remotely and therefore remained accessible. Indeed, we observed a rapid uptake of telemedicine which compensated decreased in-person psychotherapy during the shutdown, in line with findings in outpatient settings in the UK [38] and the US [39]. Psychiatric inpatient admissions that decreased during the first shutdown also recovered quickly after the relief of the measures, similar to observations from Canada [15] and Italy [40] and faster than in South Africa [17] and South Korea [18]. Importantly, the decrease in psychiatric inpatient admissions during the first shutdown was comparable to that of general emergency inpatient procedures but considerably smaller than that of general elective inpatient procedures in Switzerland [36]. Taken together, provided that the mental health status of the majority of the population in Switzerland was not affected by the pandemic in 2020 [7], access to mental healthcare appears to have been largely maintained [36, 37].

Interestingly, teleconsultations remained somewhat elevated after the first shutdown, when most mitigation measures were lifted. This is remarkable, considering that in the two years prior to the pandemic, telepsychiatry had not increased at all. The lessons learned from the partial shift to telemedicine—including remuneration aspects [9], identification of groups for whom teleconsultations are particularly appropriate [39, 41], and assessment of patient preferences [42]—could be a valuable contribution towards future mental healthcare delivery.

Whereas the mental healthcare utilization of the overall population remained similar or was slightly reduced compared to pandemic-free scenarios, this was not the case for young females. For females aged <20 years, in particular, we observed an increase in mental healthcare utilization in the second half of the year 2020, supporting the growing evidence of the negative mental health impact of the pandemic on this population [7, 9, 11, 12, 43]. Given that the pandemic has reduced social contacts and increased stress (e.g., due to uncertain job prospects) among young people [7, 43, 44], we expected increases in mental healthcare use to manifest mainly in affective and neurotic disorders. In fact, we did observe an increase in admissions for depressive and neurotic disorders and in antidepressant and anxiolytic drug claims in females aged <20 years. Nevertheless, we also saw an increase in antipsychotic medication in both females <20 years and those aged 20–30 years, which does not fit the rest of the picture. However, this could be due to the fact that the ATC code does not perfectly reflect the disorder for which the medication is prescribed. Besides, it should be noted that an increased number of medication claims does not necessarily indicate an increased drug use, but could be caused in part by precautionary purchases during times of uncertainty.

Importantly, and in contrast to results observed for young females, young males and especially those aged <20 years showed lower mental healthcare use than expected in absence of a pandemic. This is surprising, because even though young females were reportedly more affected by the pandemic than young males, the latter were still described as being vulnerable to its negative effects [7], and thus, we would have expected mental healthcare utilization to increase rather than decrease. However, our observations are consistent with a study from Ontario, Canada [9]. While the authors of this study concluded that pandemic-related changes disproportionately affected young female individuals, it is also conceivable that young male individuals, who are known to be less likely to seek help for mental health disorders [45], have been undertreated during the pandemic. In this context, it should not go unmentioned that already in 2016, a report on behalf of the Federal Office of Public Health pointed out a shortage in pediatric psychiatric care in Switzerland, both in the outpatient and inpatient settings [46]. Thus, it is well possible that the demand for mental healthcare among young people was higher than our data suggest.

### Strengths and Limitations

This was a large-scale study based on two administrative data sets from the inpatient and the outpatient setting. By combining these two settings, our study offers a detailed insight into mental healthcare utilization during the first year of the COVID-19 pandemic in Switzerland, broken down into different periods, with consideration of secular time trends, and with special attention to the particularly vulnerable youth. For the inpatient setting, the dataset comprised the complete Swiss population. For the outpatient setting, the sample covered approximately 15% of the Swiss population. We consider the outpatient sample to be largely representative of the Swiss population, given that people can freely choose their health insurance provider and that all providers offer the same basic benefit package. Moreover, there were no major differences in age and sex distributions between the two samples (Table 1).

Our study has certain limitations inherent to observational studies based on routine data. For instance, we could not adjust for two often reported determinants of a negative health impact of the COVID-19 pandemic, namely, socioeconomic status and preexisting mental health disorders [4–6, 47, 48]. Moreover, we could not assess whether there was a shift in patterns of requests for individual patients, i.e., if additional consultations were due to excessive use by some patients or a moderate increase among many patients. We could, however, observe that further consultations with a psychiatrist increased over the course of the pandemic, while first consultations did not, which speaks to the higher vulnerability of people with preexisting mental disorders. The MedStat data specifically had the limitation of a time lag in its availability, which restricted the analysis to the first year of the pandemic. This study thus focused on the early phase of the pandemic, when the most stringent mitigation measures were in place. It would be worthwhile to investigate the evolution of the observed patterns of healthcare utilization in subsequent years and to explore their interplay with the mental health status of the population. Limitations related to the insurance claims data were the following: First, we lack data from supplementary insurances and thus from psychiatric consultations with psychologists, which are sometimes reimbursed by supplementary insurances rather than mandatory insurance or paid out of pocket. Second, we lack information on (non-prescribed/non-reimbursed) over-the-counter medication (e.g., St. John’s-Wort). Third, medication was grouped according to the ATC classification system, but might sometimes be prescribed for other conditions, e.g., antidepressants for sleeping complaints [49] or anxiety.

### Conclusion

Our observations suggest that mental healthcare provision of the majority of the population could be largely maintained in the first year of the pandemic. However, special attention should be paid to young people, who were reportedly particularly vulnerable to the negative mental health effects of the pandemic and for whom access to mental healthcare was already scarce before the pandemic. In summary, our results point to the importance of monitoring mental healthcare utilization among different populations to detect irregularities in future pandemics or pandemic phases. This will help ensure that action can be taken when needed to ensure that vulnerable people receive appropriate access.
