# Supplementary material for: The Impact of COVID-19 on Mental Healthcare Utilization in Switzerland Was Strongest Among Young Females—Retrospective Study in 2018–2020
Source: Int J Public Health. 2023 May 19;68:1605839. doi: 10.3389/ijph.2023.1605839 (PMC10235482; doi:10.3389/ijph.2023.1605839)
Supplement: Supplementary file 1 [file DataSheet4.PDF]

International Journal of Public Health

“The impact of COVID-19 on mental health care utilization in Switzerland was strongest among young females – retrospective study in 2018-2020”

Supplementary File 4

Absolute and relative effects of different pandemic periods on different outcomes. Absolute effects represent the difference in incidences between the pandemic scenario and the pandemic free scenario, with 95% confidence intervals; relative effects represent these differences with respect to the pandemic free scenario, with 95% confidence intervals.

Outcome	Pandemic phase	All<br>Absolute | Relative	Females < 20 years<br>Absolute | Relative	Females 20-30 years<br>Absolute | Relative	Males < 20 years<br>Absolute | Relative	Males 20-30 years<br>Absolute | Relative
Total psychiatric inpatient admissions	Pre-shutdown	-0.7 (-3.2−1.8) | -1.4% (-7%−2.7%)	3.5 (-0.6−7.5) | 12.6% (0.1%−28%)	-4.9 (-10.2−0.4) | -10.2% (-20.7%−-0.1%)	0.4 (-2.8−3.6) | 2.2% (-9.7%−25.3%)	4.2 (-1.7−10) | 7.9% (-3.8%−21.6%)
	First shutdown	-26.8 (-32.6−-21.1) | -16.2% (-19.2%−-13.2%)	-4.7 (-15−5.5) | -5.3% (-18%−7.1%)	-24.8 (-37.6−-12.1) | -14.9% (-22%−-8.6%)	-13.3 (-21.5−-5.1) | -21.2% (-31.8%−-9.7%)	-23.3 (-38.2−-8.4) | -13.1% (-21.4%−-6.1%)
	Summer	1.4 (-13.9−16.7) | 0.3% (-1.6%−3.3%)	49.4 (26−72.7) | 17.6% (12.2%−27.8%)	-16.2 (-47.9−15.4) | -3.1% (-10.5%−4%)	9 (-10.1−28.1) | 4.9% (-5.3%−15.1%)	4 (-31.2−39.2) | 0.7% (-5.5%−5.8%)
	Second shutdown	-8.7 (-16.5−-1) | -3.9% (-6.7%−-0.2%)	32 (19.7−44.2) | 24.4% (14.4%−34.7%)	-12.3 (-29.2−4.7) | -5.6% (-13.1%−3%)	5.1 (-4.9−15) | 5.8% (-7.7%−19.1%)	-12.7 (-31−5.6) | -5.1% (-10.4%−1.6%)
Psychiatric inpatient admissions for affective disorders	Pre-shutdown	0.1 (-0.9−1) | 0.5% (-6.8%−10%)	3.2 (1.2−5.2) | 37.3% (11.2%−64.3%)	-0.2 (-2.7−2.3) | -1.6% (-22.7%−17.6%)	0.4 (-0.8−1.6) | 10.3% (-21.1%−39.4%)	2.4 (0.1−4.8) | 23.1% (-3.3%−54.5%)
	First shutdown	-8.9 (-11.6−-6.3) | -18.7% (-23.3%−-14.5%)	-1.4 (-6.5−3.7) | -4.9% (-23%−11.8%)	-3.4 (-9.4−2.6) | -7.8% (-16.7%−6.4%)	-1.9 (-4.9−1.1) | -15.6% (-36.2%−6%)	-7 (-13−-1) | -19.7% (-36.7%−-7.9%)
	Summer	3 (-2.8−8.8) | 2% (-2.9%−6.1%)	32.5 (20.6−44.3) | 44.6% (26.5%−64.1%)	0.4 (-14.5−15.4) | 0.3% (-10.2%−12.8%)	2.5 (-4.6−9.7) | 7.5% (-11%−31%)	-7.1 (-21.4−7.3) | -6.1% (-15.6%−6.4%)
	Second shutdown	1.1 (-1.9−4.1) | 1.7% (-3.4%−6.1%)	20.5 (14.3−26.7) | 56.3% (34.3%−82.7%)	2.3 (-5.8−10.4) | 4.1% (-8.6%−20.9%)	6.3 (2.6−10) | 37.9% (15.8%−62.7%)	-5.2 (-12.7−2.2) | -10.1% (-21.6%−3%)
Psychiatric inpatient admissions for neurotic disorders	Pre-shutdown	-0.3 (-0.9−0.3) | -4.4% (-11.3%−4%)	0.9 (-0.9−2.7) | 15.4% (-11.7%−41.2%)	-2.8 (-5.2−-0.4) | -28.5% (-51.2%−-6.9%)	-0.8 (-2.1−0.4) | -20.5% (-46.5%−13.1%)	-0.3 (-2.4−1.7) | -4.1% (-27%−16.7%)
	First shutdown	-4.5 (-5.9−-3.1) | -18.5% (-23.8%−-14%)	-0.1 (-4.6−4.5) | -0.3% (-19.1%−26.2%)	-8.5 (-14.5−-2.5) | -24.4% (-39.7%−-6.7%)	-5.5 (-8.6−-2.3) | -42.2% (-65.4%−-19.8%)	-7.6 (-12.4−-2.7) | -27.7% (-40.9%−-11.3%)
	Summer	-0.7 (-4.5−3.1) | -0.9% (-4.6%−3.9%)	11.1 (0.6−21.7) | 18.5% (1.9%−46.2%)	-17 (-31.6−-2.4) | -14.7% (-26.9%−-4.1%)	-1.2 (-8.8−6.5) | -3.2% (-21.7%−22.4%)	-7 (-19.2−5.1) | -8.1% (-20%−2.8%)
	Second shutdown	-2.7 (-4.6−-0.7) | -8.3% (-13.3%−-2.5%)	6.8 (1.3−12.3) | 22.9% (5.1%−50.4%)	-6.5 (-14.1−1.2) | -14.2% (-28.6%−0.4%)	-2.2 (-6.1−1.8) | -12.5% (-26.7%−9.5%)	-7.2 (-13.7−-0.6) | -19.5% (-33.6%−-6.3%)
Psychiatric inpatient admissions for psychotic disorders	Pre-shutdown	0.2 (-0.4−0.9) | 3.1% (-4.1%−12.3%)	0.3 (-0.3−0.9) | 30% (-41.2%−105.2%)	-1.2 (-3.1−0.7) | -23.9% (-61.6%−8.6%)	0.5 (-0.5−1.5) | 26.5% (-14.2%−85.9%)	1.8 (-1.1−4.7) | 12.9% (-12.4%−30.7%)
	First shutdown	-0.9 (-2.5−0.8) | -3.7% (-9.4%−2.2%)	0.3 (-1.2−1.8) | 10.1% (-22.1%−67.2%)	-2.3 (-6.7−2.1) | -12.9% (-30.5%−13.8%)	-0.3 (-2.7−2.1) | -5.2% (-40.7%−37.1%)	-0.4 (-7.6−6.8) | -0.8% (-13.8%−13.2%)
	Summer	1.9 (-1.9−5.7) | 2.5% (-2.6%−6.8%)	0.9 (-2.8−4.6) | 9% (-27.3%−60.4%)	-0.4 (-11.5−10.7) | -0.7% (-17.8%−23.1%)	1.5 (-4.5−7.5) | 8% (-14.5%−67.4%)	3.5 (-14−20.9) | 2.2% (-7.5%−16.3%)

<table>
<tr><th></th><th></th><th></th><th></th><th></th><th></th><th></th></tr>
<tr><td></td><td>Second shutdown</td><td>-1.2 (-3.2–0.8) |
-3.6% (-8.8%–3.1%)</td><td>0.8 (-1.1–2.8) |
18.5% (-23.6%–66.8%)</td><td>1.6 (-4.5–7.6) |
6.7% (-12.9%–37.4%)</td><td>1.2 (-2.1–4.4) |
14.4% (-19.2%–71.3%)</td><td>-0.2 (-9.4–8.9) |
-0.3% (-11.7%–11.1%)</td></tr>
<tr><td rowspan="4">Total outpatient psychotherapy consultations</td><td>Pre-shutdown</td><td>-26.2 (-203.6–151.1) |
-0.7% (-6%–3.7%)</td><td>107.4 (-195.3–410) |
5.3% (-8.7%–21.7%)</td><td>4.1 (-310.3–318.6) |
0.1% (-5.4%–5.6%)</td><td>29 (-276.9–335) |
1.2% (-9.8%–13.4%)</td><td>31.7 (-265.6–329) |
0.9% (-7%–10.2%)</td></tr>
<tr><td>First shutdown</td><td>-225 (-679.5–229.5) |
-1.9% (-5.4%–2.2%)</td><td>-417.5 (-1148.4–313.3) |
-6.4% (-17.1%–4.6%)</td><td>891.5 (154.3–1628.8) |
4.9% (1%–9.4%)</td><td>-1983.5 (-2715.8–-1251.2) |
-26.1% (-33.5%–-18.8%)</td><td>-390.1 (-1118.4–338.2) |
-3.5% (-8.9%–3.5%)</td></tr>
<tr><td>Summer</td><td>908.5 (-137.9–1955) |
2.7% (-0.3%–5.7%)</td><td>1552.1 (-90–3194.3) |
8.5% (-0.5%–17.8%)</td><td>2289.5 (641.2–3937.9) |
4.2% (1.1%–7.2%)</td><td>-853.3 (-2497–790.5) |
-4.1% (-11.5%–3%)</td><td>1101.1 (-538.5–2740.7) |
3.4% (-1.5%–8.6%)</td></tr>
<tr><td>Second shutdown</td><td>225.5 (-271.7–722.7) |
1.6% (-1.7%–4.8%)</td><td>1057.2 (205.7–1908.7) |
12.2% (2.6%–22.4%)</td><td>1227.9 (371.3–2084.5) |
5.4% (1.9%–9.3%)</td><td>-765 (-1617.7–87.7) |
-6.2% (-13.4%–2.2%)</td><td>271.3 (-578.4–1121) |
2.5% (-3.2%–9%)</td></tr>
<tr><td rowspan="4">Face-to-face outpatient psychotherapy consultations</td><td>Pre-shutdown</td><td>-62 (-217.8–93.8) |
-1.9% (-6.1%–2.9%)</td><td>96.7 (-188.3–381.7) |
5.1% (-9%–23%)</td><td>-54.9 (-351–241.3) |
-1.1% (-6.6%–4.5%)</td><td>4.6 (-283.2–292.5) |
0.2% (-11.5%–14.1%)</td><td>4.5 (-276.1–285) |
0.1% (-8.1%–9.9%)</td></tr>
<tr><td>First shutdown</td><td>-2402.3 (-2894.9–-1909.7) |
-22.4% (-26.3%–-18.5%)</td><td>-1781.7 (-2470–-1093.4) |
-29.5% (-39.2%–-18.5%)</td><td>-2803.3 (-3497.6–-2109) |
-16.5% (-20.1%–-13.3%)</td><td>-3288.8 (-3978.3–-2599.3) |
-47.8% (-55.6%–-40.1%)</td><td>-2055.9 (-2742.1–-1369.7) |
-20.1% (-25.8%–-13.6%)</td></tr>
<tr><td>Summer</td><td>-240.5 (-1168–686.9) |
-0.8% (-3.5%–2%)</td><td>982.7 (-563.8–2529.3) |
5.9% (-2.8%–16.1%)</td><td>740.6 (-811.7–2292.9) |
1.5% (-1.6%–4.5%)</td><td>-1147.7 (-2695.6–400.1) |
-6.2% (-13.6%–2.6%)</td><td>556.5 (-987.9–2100.9) |
1.9% (-3.1%–6.9%)</td></tr>
<tr><td>Second shutdown</td><td>-297.2 (-737.3–142.9) |
-2.2% (-5.2%–0.9%)</td><td>605.9 (-196–1407.8) |
8.1% (-1%–18.3%)</td><td>539.4 (-267.3–1346.1) |
2.8% (-0.4%–6.3%)</td><td>-887.7 (-1690.6–-84.7) |
-8.3% (-16%–0.7%)</td><td>17 (-783.4–817.4) |
0.7% (-5%–7%)</td></tr>
<tr><td rowspan="4">Telemedicine outpatient psychotherapy consultations</td><td>Pre-shutdown</td><td>43.6 (2.1–85.1) |
16.1% (-0.6%–30.2%)</td><td>12.9 (-25.4–51.1) |
7.3% (-14.9%–30.6%)</td><td>59.5 (-22.1–141) |
15.9% (-4.8%–36.1%)</td><td>39.3 (-6.6–85.2) |
15.3% (-1.5%–35.6%)</td><td>6 (-42.4–54.4) |
2.1% (-15.3%–20.3%)</td></tr>
<tr><td>First shutdown</td><td>2267.9 (2076.7–2459) |
255.4% (226%–281.8%)</td><td>1374.5 (1228.6–1520.5) |
244.2% (204.9%–289.4%)</td><td>3497.8 (3119.8–3875.8) |
281.8% (244.4%–320.8%)</td><td>1342.7 (1203.5–1482) |
181.1% (153.4%–213.1%)</td><td>1606.9 (1421.7–1792.1) |
172.6% (145.9%–201.9%)</td></tr>
<tr><td>Summer</td><td>1090.9 (860.3–1321.5) |
41.3% (31.2%–52.4%)</td><td>586.8 (398.5–775.2) |
33.4% (21.1%–48.2%)</td><td>1366.5 (910.6–1822.5) |
36.5% (23.2%–52.5%)</td><td>416.6 (228–605.1) |
18.2% (10%–27.8%)</td><td>514.6 (235.2–794.1) |
17.8% (7.3%–30.8%)</td></tr>
<tr><td>Second shutdown</td><td>548 (422.2–673.8) |
50.6% (36.5%–64.4%)</td><td>499.3 (379.6–619) |
58.4% (41.9%–77.7%)</td><td>809.9 (550.6–1069.1) |
52.6% (34.4%–73.7%)</td><td>172 (57.6–286.4) |
15.7% (5.6%–26.4%)</td><td>292.6 (145.5–439.6) |
26% (13.3%–40.9%)</td></tr>
<tr><td rowspan="4">First consultations with psychiatrist</td><td>Pre-shutdown</td><td>-1.4 (-13.3–10.5) |
-1.3% (-11.7%–10.5%)</td><td>-8 (-24.4–8.4) |
-11.2% (-32.9%–13.7%)</td><td>34 (2.8–65.2) |
19.6% (1%–40.3%)</td><td>2.1 (-14.8–18.9) |
3.2% (-20.2%–28.7%)</td><td>-13.4 (-38.5–11.6) |
-9.4% (-27%–9.5%)</td></tr>
<tr><td>First shutdown</td><td>-108.1 (-138.4–-77.8) |
-32.6% (-40.1%–-23.7%)</td><td>-105.2 (-146.8–-63.7) |
-47.1% (-60.5%–-30.1%)</td><td>-145.6 (-219.4–-71.8) |
-25% (-36.3%–-13%)</td><td>-76.8 (-119.4–-34.2) |
-38.7% (-55.4%–-20.7%)</td><td>-144.6 (-207.9–-81.4) |
-32.8% (-44.7%–-20.4%)</td></tr>
<tr><td>Summer</td><td>24.8 (-45.1–94.7) |
2.5% (-4.2%–10.3%)</td><td>-76.9 (-173.5–19.6) |
-11.5% (-22.3%–3.3%)</td><td>-98.1 (-281.2–85) |
-5.4% (-14.1%–4.5%)</td><td>-8.8 (-107.7–90.2) |
-1.5% (-17.5%–18.7%)</td><td>81.2 (-66.6–229) |
6.4% (-4.3%–19.1%)</td></tr>
<tr><td>Second shutdown</td><td>17.3 (-18.6–53.3) |
4.1% (-4.5%–13.4%)</td><td>11.5 (-38–61.1) |
3.8% (-10.9%–24.1%)</td><td>78.5 (-19.2–176.2) |
9% (-4.6%–21.4%)</td><td>6.3 (-44.5–57.1) |
3.9% (-12.6%–25.8%)</td><td>-69.6 (-145.3–6.1) |
-11.5% (-21.7%–1.7%)</td></tr>
<tr><td rowspan="3">Further consultations with psychiatrist</td><td>Pre-shutdown</td><td>-11 (-135.8–113.8) |
-0.5% (-5.9%–5.3%)</td><td>63.7 (-104.9–232.3) |
6.7% (-9.5%–28.1%)</td><td>-20.3 (-195.7–155.1) |
-0.8% (-7%–5.5%)</td><td>38.4 (-132–208.9) |
3.3% (-11.1%–18.3%)</td><td>50 (-119–219) |
2.6% (-6.2%–11.4%)</td></tr>
<tr><td>First shutdown</td><td>121.1 (-190.6–432.9) |
1.7% (-2.8%–6%)</td><td>21.9 (-386.8–430.6) |
0.7% (-10.6%–15.1%)</td><td>561.7 (149.4–973.9) |
6.5% (1.6%–11.6%)</td><td>-588.3 (-997.9–-178.7) |
-16.9% (-27.2%–-5.9%)</td><td>91.3 (-317.6–500.3) |
1.4% (-4.5%–7.8%)</td></tr>
<tr><td>Summer</td><td>857.8 (125.1–1590.5) |
4.1% (0.6%–7.9%)</td><td>1121.4 (202.6–2040.3) |
13.3% (2.7%–25.2%)</td><td>1692.7 (770.4–2615.1) |
6.5% (2.6%–10.3%)</td><td>-53.7 (-973.5–866) |
-0.6% (-9.5%–9%)</td><td>1002.1 (83–1921.2) |
5.4% (0.3%–10.7%)</td></tr>
</table>

	Second shutdown	207.7 (-141.9−557.4) | 2.2% (-1.7%−6.6%)	733.6 (257.3−1210) | 18% (7.5%−30.4%)	789.6 (310.4−1268.8) | 7.1% (3.5%−11.4%)	-166.1 (-643.1−311) | -2.4% (-11.6%−8.4%)	477.9 (1.4−954.5) | 6.3% (0.7%−12.3%)
Total psychotropic medication claims	Pre-shutdown	337 (-58.7−732.8) | 6.8% (-0.9%−16.3%)	-6.8 (-115.2−101.6) | -1.9% (-32%−31.1%)	81.7 (-48.7−212.1) | 4.3% (-2.3%−11.4%)	30.9 (-87.8−149.6) | 5.4% (-14.1%−27.2%)	203.5 (75.8−331.2) | 13.1% (5.5%−21.7%)
	First shutdown	-872.4 (-1873.7−128.8) | -5.1% (-10.6%−0.4%)	-254.5 (-513.5−4.4) | -21.4% (-37.8%−2.2%)	-316.6 (-623.8−-9.3) | -4.9% (-9.2%−-0.4%)	-396.5 (-695.6−-97.4) | -19.7% (-33.3%−-4.9%)	-112.3 (-418.1−193.5) | -2.1% (-7.2%−3%)
	Summer	-1318.9 (-3601.4−963.6) | -2.5% (-6.6%−1.2%)	-18.9 (-659−621.1) | -0.5% (-16.5%−18.2%)	650.9 (-36.9−1338.8) | 3.3% (-0.3%−6.8%)	-494.4 (-1175.8−187) | -8.4% (-18.5%−3.3%)	1315.6 (629.1−2002) | 7.9% (3.7%−12.5%)
	Second shutdown	-155.5 (-1335−1023.9) | -0.5% (-5.5%−4.8%)	534.4 (194.4−874.4) | 37.1% (12.1%−65.9%)	534.2 (177−891.5) | 6.4% (2.6%−10.9%)	-74.9 (-426.8−277) | -2.5% (-13.2%−9.1%)	658 (301.9−1014) | 9% (3.8%−13.8%)
Antidepressants	Pre-shutdown	139.7 (-10.6−289.9) | 7.9% (-0.8%−16.3%)	4.5 (-14.8−23.8) | 5.4% (-16.5%−28.8%)	43.1 (-89.5−175.8) | 4.9% (-10.1%−20.4%)	0.4 (-13−13.8) | 0.9% (-26.7%−31.2%)	108.2 (38.1−178.4) | 22.7% (6.8%−39.3%)
	First shutdown	-404.4 (-782.4−-26.3) | -6.6% (-12.4%−-0.2%)	-42.7 (-91.4−6) | -14.3% (-28.5%−1.6%)	-281.2 (-593.6−31.2) | -9.2% (-18.7%−0.8%)	-3.8 (-36.2−28.6) | -2.3% (-20.2%−19.2%)	13.4 (-154.9−181.6) | 0.8% (-9.6%−11.2%)
	Summer	-643.2 (-1500.2−213.9) | -3.4% (-7.5%−1.2%)	46.4 (-64.5−157.2) | 4.9% (-6.6%−18.7%)	-293.5 (-1070.5−483.5) | -3% (-10.5%−5.3%)	-45.7 (-125.2−33.9) | -9.2% (-23.4%−5.4%)	966.4 (588.6−1344.3) | 19.2% (11.6%−28.9%)
	Second shutdown	-104.1 (-547.7−339.5) | -1.1% (-6.1%−4.5%)	179.4 (122.1−236.6) | 40.2% (25.2%−57.6%)	261.7 (-153.8−677.2) | 6.4% (-4%−17.3%)	36.6 (-5.6−78.7) | 17.9% (-0.5%−40.2%)	340.4 (144.4−536.4) | 14.5% (6.2%−23.5%)
Anxiolytics	Pre-shutdown	54.6 (-30.2−139.3) | 5.1% (-2.4%−12.8%)	0 (-11.3−11.3) | -0.1% (-28.2%−28.1%)	44.8 (-8.6−98.2) | 14.6% (-1.5%−30.7%)	2.9 (-7.4−13.2) | 6.9% (-16.3%−32.9%)	23.3 (-21.2−67.9) | 8.2% (-7.5%−24.4%)
	First shutdown	-87.6 (-294.6−119.4) | -2.4% (-7.5%−2.9%)	-44.6 (-71.6−-17.6) | -34.6% (-52.3%−-15.8%)	100.9 (-31.2−233) | 9.5% (-2.1%−22.3%)	-2.7 (-27.4−22) | -2% (-17.6%−16.1%)	-23 (-132.9−86.9) | -2.3% (-13.3%−9.3%)
	Summer	-343.7 (-848.3−160.8) | -3.1% (-7.2%−1.4%)	-35.7 (-102.2−30.9) | -8.9% (-23.7%−10.1%)	185.9 (-132.2−504.1) | 5.5% (-4.1%−15.4%)	-71.4 (-132.2−-10.6) | -16.1% (-27.6%−-4.4%)	207.1 (-58.1−472.4) | 6.6% (-2.1%−16.2%)
	Second shutdown	26.6 (-239.6−292.8) | 0.8% (-4.2%−6.7%)	55.3 (19.9−90.7) | 33.3% (11.9%−61.6%)	119.6 (-44−283.2) | 8.1% (-1.7%−21.3%)	-34.7 (-66.9−-2.4) | -17% (-30.4%−-0.6%)	247.7 (111.1−384.3) | 18.4% (7%−29.7%)
Antipsychotics	Pre-shutdown	30.1 (-73.2−133.5) | 2.9% (-6.4%−12.8%)	16.5 (4.3−28.8) | 44.6% (7.9%−90.7%)	1.6 (-45.8−49) | 0.5% (-14.8%−15.3%)	5.3 (-11.8−22.4) | 6.7% (-14.2%−28%)	42.6 (-24.2−109.4) | 9.1% (-4.1%−24.3%)
	First shutdown	-133.7 (-389.9−122.4) | -3.7% (-10.6%−3.8%)	20.8 (-10.2−51.9) | 15.3% (-7.4%−42.6%)	62.9 (-56.9−182.7) | 5.8% (-5.2%−17.3%)	19.6 (-23.7−62.8) | 6.9% (-7.3%−24.3%)	96 (-67.8−259.8) | 6.1% (-4.4%−16.8%)
	Summer	-566.7 (-1182.8−49.3) | -5% (-10.3%−0.3%)	154.1 (82.7−225.5) | 35.3% (15.6%−58.2%)	694.7 (417.4−972.1) | 22.2% (12.6%−33.1%)	-18.4 (-117.8−81) | -2.1% (-13.1%−9.4%)	143.6 (-225.4−512.6) | 2.8% (-4.1%−9.7%)
	Second shutdown	-112.4 (-432.8−207.9) | -2.7% (-9.5%−3.4%)	108 (71.2−144.7) | 55.7% (33.7%−84.1%)	363.7 (221.3−506.1) | 26.9% (14.8%−38.7%)	-47.8 (-99−3.4) | -10.1% (-20.8%−1.3%)	89.2 (-101.9−280.3) | 3.7% (-4.1%−12.1%)
