# Supplementary material for: The Impact of COVID-19 on Mental Healthcare Utilization in Switzerland Was Strongest Among Young Females—Retrospective Study in 2018–2020
Source: Int J Public Health. 2023 May 19;68:1605839. doi: 10.3389/ijph.2023.1605839 (PMC10235482; doi:10.3389/ijph.2023.1605839)
Supplement: Supplementary file 2 [file DataSheet3.PDF]

International Journal of Public Health

"The impact of COVID-19 on mental health care utilization in Switzerland was strongest among young females – retrospective study in 2018-2020"

Supplementary File 3

Overview of regression coefficients, total population

Total psychiatric admissions

Variable	Coefficient estimate	Standard error	p-Value
Intercept	23.63	0.21	< 0.001
Overall trend	0.01	0.00	0.087
Ascension Day	-1.66	0.57	0.004
Spring/Easter break	-0.86	0.42	0.040
Autumn holidays	-0.46	0.46	0.317
Midsummer holidays	-1.32	0.59	0.027
New Year's holidays	-4.37	0.55	< 0.001
Pentecost	-0.90	0.59	0.131
Summer holidays	-0.31	0.36	0.390
Christmas holidays	0.56	0.66	0.394
Pre-shutdown level	0.70	1.55	0.651
Pre-shutdown trend	-0.52	0.69	0.450
First shutdown level	-7.72	0.67	< 0.001
First shutdown trend	0.86	0.14	< 0.001
Summer level	0.64	0.56	0.255
Summer trend	-0.05	0.04	0.182
Second shutdown level	-1.92	0.76	0.013
Second shutdown trend	0.17	0.11	0.134

Affective disorders

Variable	Coefficient estimate	Standard error	p-Value
Intercept	7.22	0.08	< 0.001
Overall trend	0.00	0.00	0.110
Ascension Day	-0.67	0.25	0.007
Spring/Easter break	-0.34	0.18	0.064
Autumn holidays	-0.21	0.18	0.229
Midsummer holidays	-0.72	0.25	0.005
New Year's holidays	-1.72	0.26	< 0.001
Pentecost	-0.04	0.24	0.877
Summer holidays	-0.21	0.14	0.142
Christmas holidays	0.16	0.23	0.491
Pre-shutdown level	0.38	0.59	0.516
Pre-shutdown trend	-0.17	0.27	0.522
First shutdown level	-2.63	0.37	< 0.001
First shutdown trend	0.30	0.07	< 0.001
Summer level	0.07	0.22	0.734
Summer trend	0.01	0.01	0.706
Second shutdown level	-0.36	0.31	0.237
Second shutdown trend	0.09	0.05	0.066

Neurotic disorders

Variable	Coefficient estimate	Standard error	p-Value
Intercept	3.59	0.05	< 0.001
Overall trend	0.00	0.00	0.724
Ascension Day	-0.31	0.15	0.045
Spring/Easter break	-0.43	0.10	< 0.001
Autumn holidays	-0.08	0.11	0.478
Midsummer holidays	-0.39	0.14	0.007
New Year's holidays	-0.89	0.14	< 0.001
Pentecost	-0.05	0.16	0.761
Summer holidays	-0.20	0.08	0.013
Christmas holidays	-0.15	0.16	0.332
Pre-shutdown level	0.22	0.40	0.585
Pre-shutdown trend	-0.19	0.18	0.293
First shutdown level	-1.69	0.17	< 0.001
First shutdown trend	0.23	0.04	< 0.001
Summer level	0.03	0.13	0.821
Summer trend	-0.01	0.01	0.549
Second shutdown level	-0.44	0.19	0.021
Second shutdown trend	0.03	0.03	0.343

Psychotic disorders

Variable	Coefficient estimate	Standard error	p-Value
Intercept	3.39	0.05	< 0.001
Overall trend	0.00	0.00	0.352
Ascension Day	-0.10	0.16	0.510
Spring/Easter break	0.09	0.12	0.451
Autumn holidays	-0.07	0.12	0.533
Midsummer holidays	0.20	0.16	0.207
New Year's holidays	-0.47	0.16	0.004
Pentecost	-0.04	0.15	0.772
Summer holidays	0.12	0.10	0.240
Christmas holidays	0.35	0.16	0.030
Pre-shutdown level	-0.08	0.39	0.847
Pre-shutdown trend	0.09	0.18	0.610
First shutdown level	-0.33	0.22	0.126
First shutdown trend	0.05	0.04	0.256
Summer level	0.26	0.14	0.064
Summer trend	-0.01	0.01	0.112
Second shutdown level	-0.57	0.20	0.005
Second shutdown trend	0.08	0.03	0.010

Total psychiatric consultations

Variable	Coefficient estimate	Standard error	p-Value
Intercept	1719.92	15.45	< 0.001
Overall trend	-0.19	0.21	0.362
Ascension Day	-378.32	51.10	< 0.001
Spring/Easter break	-209.77	43.37	< 0.001
Autumn holidays	-72.09	55.36	0.195
Midsummer holidays	-301.89	132.09	0.024
New Year's holidays	-1137.95	143.08	< 0.001
Pentecost	-282.12	46.87	< 0.001
Summer holidays	-98.78	69.04	0.155
Christmas holidays	34.85	59.56	0.560
Pre-shutdown level	0.04	108.28	1.000
Pre-shutdown trend	-6.57	47.93	0.891
First shutdown level	-180.72	62.70	0.005
First shutdown trend	33.02	11.82	0.006
Summer level	75.96	37.34	0.044
Summer trend	-2.89	2.51	0.251
Second shutdown level	12.40	55.49	0.824
Second shutdown trend	2.30	8.91	0.797

In-person consultations

Variable	Coefficient estimate	Standard error	p-Value
Intercept	1591.47	13.75	< 0.001
Overall trend	-0.19	0.18	0.303
Ascension Day	-354.64	46.62	< 0.001
Spring/Easter break	-254.90	42.28	< 0.001
Autumn holidays	-60.45	51.72	0.245
Midsummer holidays	-246.34	123.91	0.049
New Year's holidays	-1038.60	139.08	< 0.001
Pentecost	-255.06	41.92	< 0.001
Summer holidays	-75.95	64.68	0.243
Christmas holidays	23.62	56.62	0.677
Pre-shutdown level	16.61	95.60	0.862
Pre-shutdown trend	-23.81	42.63	0.578
First shutdown level	-525.67	70.29	< 0.001
First shutdown trend	40.55	12.89	0.002
Summer level	-33.93	34.24	0.324
Summer trend	1.92	2.29	0.403
Second shutdown level	-27.20	49.70	0.585
Second shutdown trend	-1.06	8.01	0.895

Teleconsultations

Variable	Coefficient estimate	Standard error	p-Value
Intercept	148.18	2.95	< 0.001
Overall trend	-0.11	0.04	0.009
Ascension Day	-14.91	9.76	0.129
Spring/Easter break	-28.07	6.90	< 0.001
Autumn holidays	-15.18	6.58	0.022
Midsummer holidays	-68.72	6.56	< 0.001
New Year's holidays	-103.60	3.37	< 0.001
Pentecost	-14.69	9.77	0.135
Summer holidays	-39.50	4.44	< 0.001
Christmas holidays	28.27	11.39	0.014
Pre-shutdown level	-12.40	26.11	0.635
Pre-shutdown trend	17.10	12.61	0.177
First shutdown level	354.31	29.30	< 0.001
First shutdown trend	-6.74	5.57	0.228
Summer level	96.87	10.16	< 0.001
Summer trend	-3.94	0.67	< 0.001
Second shutdown level	70.75	12.99	< 0.001
Second shutdown trend	-1.79	1.49	0.230

First consultations

Variable	Coefficient estimate	Standard error	p-Value
Intercept	52.85	0.96	< 0.001
Overall trend	-0.02	0.01	0.250
Ascension Day	-11.28	2.91	< 0.001
Spring/Easter break	-12.40	2.20	< 0.001
Autumn holidays	-2.53	2.21	0.255
Midsummer holidays	-17.33	2.99	< 0.001
New Year's holidays	-42.69	2.19	< 0.001
Pentecost	-9.20	2.89	0.002
Summer holidays	-6.30	1.83	< 0.001
Christmas holidays	-8.96	2.97	0.003
Pre-shutdown level	-1.09	7.27	0.881
Pre-shutdown trend	0.20	3.31	0.953
First shutdown level	-19.78	4.02	< 0.001
First shutdown trend	0.96	0.76	0.205
Summer level	2.91	2.62	0.269
Summer trend	-0.15	0.17	0.388
Second shutdown level	2.28	3.56	0.523
Second shutdown trend	-0.06	0.50	0.898

Further consultations

Variable	Coefficient estimate	Standard error	p-Value
Intercept	1138.68	10.27	< 0.001
Overall trend	-0.60	0.14	< 0.001
Ascension Day	-241.21	35.59	< 0.001
Spring/Easter break	-128.28	29.38	< 0.001
Autumn holidays	-55.59	36.03	0.125
Midsummer holidays	-213.12	87.18	0.016
New Year's holidays	-714.63	98.31	< 0.001
Pentecost	-181.64	32.65	< 0.001
Summer holidays	-80.31	44.47	0.073
Christmas holidays	43.12	37.27	0.250
Pre-shutdown level	-19.30	76.51	0.801
Pre-shutdown trend	6.91	33.67	0.838
First shutdown level	-71.08	42.80	0.099
First shutdown trend	19.64	8.05	0.016
Summer level	63.45	26.10	0.017
Summer trend	-2.04	1.76	0.250
Second shutdown level	27.84	39.34	0.481
Second shutdown trend	-0.86	6.27	0.890

Total psychotropic medication

Variable	Coefficient estimate	Standard error	p-Value
Intercept	2701.72	31.35	< 0.001
Overall trend	-1.63	0.47	< 0.001
Ascension Day	-243.32	96.27	0.013
Spring/Easter break	-244.71	73.79	0.001
Autumn holidays	-56.68	75.51	0.454
Midsummer holidays	-345.65	103.51	0.001
New Year's holidays	-810.12	75.75	< 0.001
Pentecost	-221.90	95.17	0.021
Summer holidays	-192.15	66.82	0.005
Christmas holidays	579.43	99.86	< 0.001
Pre-shutdown level	218.55	238.04	0.360
Pre-shutdown trend	-25.02	108.23	0.818
First shutdown level	144.44	134.50	0.285
First shutdown trend	-59.79	25.31	0.020
Summer level	-91.35	85.99	0.290
Summer trend	2.62	5.64	0.643
Second shutdown level	-75.94	117.57	0.519
Second shutdown trend	10.66	16.52	0.520

Antipsychotics

Variable	Coefficient estimate	Standard error	p-Value
Intercept	536.75	8.47	< 0.001
Overall trend	-0.10	0.13	0.448
Ascension Day	-25.47	25.62	0.322
Spring/Easter break	-43.64	18.83	0.022
Autumn holidays	10.41	18.76	0.580
Midsummer holidays	-16.42	25.27	0.517
New Year's holidays	-106.33	18.36	< 0.001
Pentecost	-30.50	25.48	0.233
Summer holidays	-35.12	13.70	0.011
Christmas holidays	97.04	25.93	< 0.001
Pre-shutdown level	88.96	65.54	0.177
Pre-shutdown trend	-36.94	30.03	0.221
First shutdown level	3.71	34.61	0.915
First shutdown trend	-5.07	6.64	0.446
Summer level	-13.71	22.33	0.540
Summer trend	-1.00	1.45	0.491
Second shutdown level	1.91	31.20	0.951
Second shutdown trend	-2.62	4.42	0.555

Antidepressants

Variable	Coefficient estimate	Standard error	p-Value
Intercept	948.12	11.83	< 0.001
Overall trend	-0.43	0.18	0.015
Ascension Day	-81.21	36.45	0.028
Spring/Easter break	-84.83	28.34	0.003
Autumn holidays	-22.12	29.42	0.453
Midsummer holidays	-123.95	41.02	0.003
New Year's holidays	-275.25	29.97	< 0.001
Pentecost	-82.85	36.02	0.023
Summer holidays	-70.18	27.48	0.012
Christmas holidays	219.76	38.57	< 0.001
Pre-shutdown level	99.45	89.73	0.270
Pre-shutdown trend	-14.81	40.76	0.717
First shutdown level	60.10	51.38	0.244
First shutdown trend	-26.19	9.68	0.008
Summer level	-51.79	32.36	0.112
Summer trend	1.88	2.12	0.378
Second shutdown level	-28.40	44.47	0.524
Second shutdown trend	3.06	6.26	0.625

Anxiolytics

Variable	Coefficient estimate	Standard error	p-Value
Intercept	582.58	6.97	< 0.001
Overall trend	-0.42	0.10	< 0.001
Ascension Day	-52.87	21.20	0.014
Spring/Easter break	-57.14	15.51	< 0.001
Autumn holidays	-11.01	15.43	0.477
Midsummer holidays	-83.93	20.80	< 0.001
New Year's holidays	-167.81	15.06	< 0.001
Pentecost	-37.63	21.06	0.076
Summer holidays	-42.77	10.95	< 0.001
Christmas holidays	151.90	21.46	< 0.001
Pre-shutdown level	16.22	54.50	0.766
Pre-shutdown trend	5.53	25.02	0.826
First shutdown level	52.29	28.49	0.069
First shutdown trend	-14.40	5.51	0.010
Summer level	-7.64	18.04	0.673
Summer trend	-0.67	1.19	0.575
Second shutdown level	-7.00	25.83	0.787
Second shutdown trend	1.81	3.66	0.622

Overview of regression coefficients, subgroup

Total psychiatric admissions

Variable	Coefficient estimate	Standard error	p-Value
Intercept	14.26	0.31	< 0.001
Ascension Day	-1.74	0.52	< 0.001
Spring/Easter break	-0.72	0.39	0.066
Autumn holidays	-1.29	0.40	0.001
Midsummer holidays	-1.76	0.54	0.001
New Year's holidays	-4.78	0.54	< 0.001
Pentecost	0.16	0.51	0.760
Summer holidays	-0.71	0.32	0.030
Christmas holidays	-0.88	0.53	0.096
Female, 20–30 years (overall level)	7.38	0.53	< 0.001
Male, < 20 years (overall level)	-5.17	0.40	< 0.001
Male, 20–30 years (overall level)	10.86	0.57	< 0.001
Female, < 20 years (overall trend)	0.00	0.00	0.419
Female, 20–30 years (overall trend)	0.02	0.01	0.003
Male, < 20 years (overall trend)	0.00	0.00	0.797
Male, 20–30 years (overall trend)	0.01	0.01	0.047
Female, < 20 years (pre-shutdown level)	0.97	2.42	0.688
Female, 20–30 years (pre-shutdown level)	0.36	3.39	0.914
Male, < 20 years (pre-shutdown level)	0.83	1.96	0.671
Male, 20–30 years (pre-shutdown level)	4.41	3.62	0.224
Female, < 20 years (pre-shutdown trend)	0.38	1.10	0.729
Female, 20–30 years (pre-shutdown trend)	-1.40	1.55	0.370
Male, < 20 years (pre-shutdown trend)	-0.32	0.89	0.723
Male, 20–30 years (pre-shutdown trend)	-1.16	1.65	0.482
Female, < 20 years (first shutdown level)	-2.02	1.38	0.145
Female, 20–30 years (first shutdown level)	-6.88	1.78	< 0.001
Male, < 20 years (first shutdown level)	-2.00	1.09	0.066
Male, 20–30 years (first shutdown level)	-10.27	1.97	< 0.001
Female, < 20 years (first shutdown trend)	0.30	0.26	0.249
Female, 20–30 years (first shutdown trend)	0.74	0.34	0.030
Male, < 20 years (first shutdown trend)	0.02	0.20	0.913
Male, 20–30 years (first shutdown trend)	1.54	0.37	< 0.001
Female, < 20 years (summer level)	1.64	0.87	0.061
Female, 20–30 years (summer level)	-1.54	1.10	0.163
Male, < 20 years (summer level)	-0.12	0.71	0.871
Male, 20–30 years (summer level)	2.80	1.29	0.031
Female, < 20 years (summer trend)	0.05	0.06	0.377
Female, 20–30 years (summer trend)	0.07	0.07	0.350
Male, < 20 years (summer trend)	0.04	0.05	0.341
Male, 20–30 years (summer trend)	-0.22	0.08	0.009
Female, < 20 years (second shutdown level)	4.96	1.24	< 0.001
Female, 20–30 years (second shutdown level)	-0.82	1.64	0.617
Male, < 20 years (second shutdown level)	-0.90	0.99	0.363
Male, 20–30 years (second shutdown level)	-4.89	1.78	0.006
Female, < 20 years (second shutdown trend)	-0.26	0.19	0.174
Female, 20–30 years (second shutdown trend)	-0.10	0.25	0.689
Male, < 20 years (second shutdown trend)	0.27	0.15	0.076
Male, 20–30 years (second shutdown trend)	0.63	0.27	0.018

Affective disorders

Variable	Coefficient estimate	Standard error	p-Value
Intercept	4.20	0.16	< 0.001
Ascension Day	-0.62	0.22	0.005
Spring/Easter break	-0.11	0.16	0.496
Autumn holidays	-0.44	0.16	0.006
Midsummer holidays	-0.58	0.22	0.008
New Year's holidays	-1.57	0.22	< 0.001
Pentecost	0.30	0.22	0.166
Summer holidays	-0.26	0.12	0.036
Christmas holidays	-0.21	0.22	0.336
Female, 20–30 years (overall level)	1.97	0.26	< 0.001
Male, < 20 years (overall level)	-2.30	0.19	< 0.001
Male, 20–30 years (overall level)	0.70	0.25	0.005
Female, < 20 years (overall trend)	0.00	0.00	0.351
Female, 20–30 years (overall trend)	0.00	0.00	0.809
Male, < 20 years (overall trend)	0.00	0.00	0.649
Male, 20–30 years (overall trend)	0.00	0.00	0.147
Female, < 20 years (pre-shutdown level)	0.55	1.22	0.650
Female, 20–30 years (pre-shutdown level)	0.41	1.63	0.800
Male, < 20 years (pre-shutdown level)	0.51	0.74	0.488
Male, 20–30 years (pre-shutdown level)	3.30	1.48	0.026
Female, < 20 years (pre-shutdown trend)	0.52	0.56	0.348
Female, 20–30 years (pre-shutdown trend)	-0.26	0.75	0.731
Male, < 20 years (pre-shutdown trend)	-0.16	0.34	0.639
Male, 20–30 years (pre-shutdown trend)	-1.04	0.68	0.126
Female, < 20 years (first shutdown level)	0.15	0.68	0.824
Female, 20–30 years (first shutdown level)	-1.70	0.85	0.045
Male, < 20 years (first shutdown level)	-0.53	0.40	0.183
Male, 20–30 years (first shutdown level)	-2.12	0.80	0.008
Female, < 20 years (first shutdown trend)	-0.08	0.13	0.536
Female, 20–30 years (first shutdown trend)	0.27	0.16	0.097
Male, < 20 years (first shutdown trend)	0.06	0.08	0.443
Male, 20–30 years (first shutdown trend)	0.25	0.15	0.096
Female, < 20 years (summer level)	0.48	0.44	0.280
Female, 20–30 years (summer level)	0.21	0.52	0.686
Male, < 20 years (summer level)	-0.12	0.26	0.649
Male, 20–30 years (summer level)	-0.67	0.52	0.200
Female, < 20 years (summer trend)	0.08	0.03	0.004
Female, 20–30 years (summer trend)	-0.02	0.03	0.641
Male, < 20 years (summer trend)	0.02	0.02	0.247
Male, 20–30 years (summer trend)	0.03	0.03	0.386
Female, < 20 years (second shutdown level)	2.71	0.61	< 0.001
Female, 20–30 years (second shutdown level)	-0.34	0.78	0.661
Male, < 20 years (second shutdown level)	-0.50	0.37	0.176
Male, 20–30 years (second shutdown level)	-1.90	0.73	0.009
Female, < 20 years (second shutdown trend)	-0.08	0.09	0.398
Female, 20–30 years (second shutdown trend)	0.11	0.12	0.355
Male, < 20 years (second shutdown trend)	0.22	0.06	< 0.001
Male, 20–30 years (second shutdown trend)	0.24	0.11	0.029

Neurotic disorders

Variable	Coefficient estimate	Standard error	p-Value
Intercept	4.06	0.14	< 0.001
Ascension Day	-0.54	0.21	0.011
Spring/Easter break	-0.45	0.16	0.005
Autumn holidays	-0.35	0.16	0.027
Midsummer holidays	-0.93	0.21	< 0.001
New Year's holidays	-1.16	0.22	< 0.001
Pentecost	0.16	0.21	0.449
Summer holidays	-0.39	0.12	0.001
Christmas holidays	-0.37	0.22	0.088
Female, 20–30 years (overall level)	0.58	0.24	0.017
Male, < 20 years (overall level)	-2.03	0.17	< 0.001
Male, 20–30 years (overall level)	-0.55	0.22	0.012
Female, < 20 years (overall trend)	-0.01	0.00	< 0.001
Female, 20–30 years (overall trend)	0.00	0.00	0.156
Male, < 20 years (overall trend)	0.00	0.00	0.618
Male, 20–30 years (overall trend)	0.00	0.00	0.087
Female, < 20 years (pre-shutdown level)	0.86	1.09	0.430
Female, 20–30 years (pre-shutdown level)	-0.33	1.52	0.829
Male, < 20 years (pre-shutdown level)	-0.68	0.79	0.386
Male, 20–30 years (pre-shutdown level)	-0.83	1.31	0.530
Female, < 20 years (pre-shutdown trend)	-0.20	0.49	0.687
Female, 20–30 years (pre-shutdown trend)	-0.54	0.70	0.441
Male, < 20 years (pre-shutdown trend)	0.13	0.36	0.710
Male, 20–30 years (pre-shutdown trend)	0.33	0.60	0.585
Female, < 20 years (first shutdown level)	-0.73	0.60	0.225
Female, 20–30 years (first shutdown level)	-2.19	0.81	0.007
Male, < 20 years (first shutdown level)	-1.38	0.42	0.001
Male, 20–30 years (first shutdown level)	-2.12	0.69	0.002
Female, < 20 years (first shutdown trend)	0.16	0.11	0.153
Female, 20–30 years (first shutdown trend)	0.22	0.15	0.157
Male, < 20 years (first shutdown trend)	0.13	0.08	0.093
Male, 20–30 years (first shutdown trend)	0.23	0.13	0.079
Female, < 20 years (summer level)	0.64	0.39	0.103
Female, 20–30 years (summer level)	-1.56	0.52	0.003
Male, < 20 years (summer level)	-0.26	0.28	0.350
Male, 20–30 years (summer level)	0.13	0.42	0.762
Female, < 20 years (summer trend)	-0.01	0.03	0.662
Female, 20–30 years (summer trend)	0.07	0.03	0.050
Male, < 20 years (summer trend)	0.02	0.02	0.332
Male, 20–30 years (summer trend)	-0.04	0.03	0.173
Female, < 20 years (second shutdown level)	0.94	0.55	0.085
Female, 20–30 years (second shutdown level)	-1.06	0.74	0.155
Male, < 20 years (second shutdown level)	-0.51	0.39	0.188
Male, 20–30 years (second shutdown level)	-1.21	0.64	0.057
Female, < 20 years (second shutdown trend)	-0.03	0.08	0.674
Female, 20–30 years (second shutdown trend)	0.06	0.11	0.583
Male, < 20 years (second shutdown trend)	0.05	0.06	0.402
Male, 20–30 years (second shutdown trend)	0.08	0.10	0.429

Psychotic disorders

Variable	Coefficient estimate	Standard error	p-Value
Intercept	0.38	0.05	< 0.001
Ascension Day	-0.02	0.12	0.872
Spring/Easter break	0.01	0.09	0.949
Autumn holidays	-0.14	0.09	0.121
Midsummer holidays	-0.08	0.12	0.511
New Year's holidays	-0.01	0.13	0.967
Pentecost	0.11	0.12	0.368
Summer holidays	-0.06	0.06	0.367
Christmas holidays	0.09	0.13	0.502
Female, 20–30 years (overall level)	2.09	0.16	< 0.001
Male, < 20 years (overall level)	0.51	0.10	< 0.001
Male, 20–30 years (overall level)	6.76	0.24	< 0.001
Female, < 20 years (overall trend)	0.00	0.00	0.305
Female, 20–30 years (overall trend)	0.00	0.00	0.776
Male, < 20 years (overall trend)	0.00	0.00	0.968
Male, 20–30 years (overall trend)	0.00	0.00	0.965
Female, < 20 years (pre-shutdown level)	0.54	0.40	0.179
Female, 20–30 years (pre-shutdown level)	-1.29	1.21	0.285
Male, < 20 years (pre-shutdown level)	0.73	0.65	0.259
Male, 20–30 years (pre-shutdown level)	1.01	1.83	0.579
Female, < 20 years (pre-shutdown trend)	-0.20	0.18	0.281
Female, 20–30 years (pre-shutdown trend)	0.34	0.55	0.538
Male, < 20 years (pre-shutdown trend)	-0.25	0.30	0.404
Male, 20–30 years (pre-shutdown trend)	-0.05	0.84	0.952
Female, < 20 years (first shutdown level)	0.16	0.21	0.428
Female, 20–30 years (first shutdown level)	-0.43	0.63	0.490
Male, < 20 years (first shutdown level)	-0.07	0.34	0.832
Male, 20–30 years (first shutdown level)	-2.14	0.97	0.028
Female, < 20 years (first shutdown trend)	-0.03	0.04	0.517
Female, 20–30 years (first shutdown trend)	0.02	0.12	0.846
Male, < 20 years (first shutdown trend)	0.01	0.06	0.930
Male, 20–30 years (first shutdown trend)	0.46	0.18	0.012
Female, < 20 years (summer level)	-0.04	0.13	0.779
Female, 20–30 years (summer level)	-0.18	0.38	0.631
Male, < 20 years (summer level)	-0.01	0.21	0.961
Male, 20–30 years (summer level)	1.49	0.63	0.018
Female, < 20 years (summer trend)	0.01	0.01	0.443
Female, 20–30 years (summer trend)	0.01	0.02	0.582
Male, < 20 years (summer trend)	0.01	0.01	0.628
Male, 20–30 years (summer trend)	-0.11	0.04	0.005
Female, < 20 years (second shutdown level)	0.36	0.20	0.066
Female, 20–30 years (second shutdown level)	1.10	0.58	0.059
Male, < 20 years (second shutdown level)	0.00	0.31	0.995
Male, 20–30 years (second shutdown level)	-1.60	0.88	0.071
Female, < 20 years (second shutdown trend)	-0.05	0.03	0.105
Female, 20–30 years (second shutdown trend)	-0.17	0.09	0.052
Male, < 20 years (second shutdown trend)	0.02	0.05	0.615
Male, 20–30 years (second shutdown trend)	0.29	0.13	0.031

Total psychiatric consultations

Variable	Coefficient estimate	Standard error	p-Value
Intercept	1009.72	22.34	< 0.001
Ascension Day	-344.31	36.24	< 0.001
Spring/Easter break	-382.05	30.58	< 0.001
Autumn holidays	-40.84	40.91	0.319
Midsummer holidays	-306.63	61.17	< 0.001
New Year's holidays	-1235.91	46.81	< 0.001
Pentecost	-262.01	35.87	< 0.001
Summer holidays	-147.51	44.19	< 0.001
Christmas holidays	37.35	50.62	0.461
Female, 20–30 years (overall level)	1571.45	30.68	< 0.001
Male, < 20 years (overall level)	124.90	30.65	< 0.001
Male, 20–30 years (overall level)	685.70	30.63	< 0.001
Female, < 20 years (overall trend)	0.01	0.34	0.976
Female, 20–30 years (overall trend)	0.60	0.34	0.079
Male, < 20 years (overall trend)	0.07	0.34	0.847
Male, 20–30 years (overall trend)	-0.49	0.34	0.149
Female, < 20 years (pre-shutdown level)	96.17	179.16	0.592
Female, 20–30 years (pre-shutdown level)	10.75	197.12	0.957
Male, < 20 years (pre-shutdown level)	174.94	182.31	0.338
Male, 20–30 years (pre-shutdown level)	-5.05	175.54	0.977
Female, < 20 years (pre-shutdown trend)	-21.24	81.22	0.794
Female, 20–30 years (pre-shutdown trend)	-4.34	90.10	0.962
Male, < 20 years (pre-shutdown trend)	-80.21	82.72	0.333
Male, 20–30 years (pre-shutdown trend)	10.46	79.58	0.895
Female, < 20 years (first shutdown level)	-214.73	103.02	0.038
Female, 20–30 years (first shutdown level)	-157.65	105.96	0.137
Male, < 20 years (first shutdown level)	-504.59	103.76	< 0.001
Male, 20–30 years (first shutdown level)	-131.24	101.65	0.197
Female, < 20 years (first shutdown trend)	34.46	19.56	0.079
Female, 20–30 years (first shutdown trend)	63.34	20.30	0.002
Male, < 20 years (first shutdown trend)	49.16	19.75	0.013
Male, 20–30 years (first shutdown trend)	16.78	19.22	0.383
Female, < 20 years (summer level)	50.85	62.34	0.415
Female, 20–30 years (summer level)	173.71	62.90	0.006
Male, < 20 years (summer level)	-64.55	62.48	0.302
Male, 20–30 years (summer level)	96.97	62.12	0.119
Female, < 20 years (summer trend)	1.64	4.11	0.690
Female, 20–30 years (summer trend)	-5.80	4.16	0.164
Male, < 20 years (summer trend)	2.15	4.12	0.603
Male, 20–30 years (summer trend)	-3.91	4.09	0.340
Female, < 20 years (second shutdown level)	41.89	86.77	0.629
Female, 20–30 years (second shutdown level)	44.51	88.47	0.615
Male, < 20 years (second shutdown level)	-220.25	87.19	0.012
Male, 20–30 years (second shutdown level)	-79.29	86.10	0.358
Female, < 20 years (second shutdown trend)	13.74	12.18	0.260
Female, 20–30 years (second shutdown trend)	16.71	12.43	0.179
Male, < 20 years (second shutdown trend)	24.59	12.24	0.045
Male, 20–30 years (second shutdown trend)	19.90	12.08	0.100

In-person consultations

Variable	Coefficient estimate	Standard error	p-Value
Intercept	925.52	21.04	< 0.001
Ascension Day	-332.29	34.13	< 0.001
Spring/Easter break	-374.55	28.81	< 0.001
Autumn holidays	-24.75	38.56	0.521
Midsummer holidays	-270.64	57.78	< 0.001
New Year's holidays	-1138.62	44.19	< 0.001
Pentecost	-237.40	33.79	< 0.001
Summer holidays	-129.89	41.78	0.002
Christmas holidays	31.87	47.73	0.505
Female, 20–30 years (overall level)	1465.27	28.89	< 0.001
Male, < 20 years (overall level)	101.28	28.87	< 0.001
Male, 20–30 years (overall level)	638.19	28.85	< 0.001
Female, < 20 years (overall trend)	0.02	0.32	0.958
Female, 20–30 years (overall trend)	0.70	0.32	0.029
Male, < 20 years (overall trend)	0.03	0.32	0.914
Male, 20–30 years (overall trend)	-0.48	0.32	0.133
Female, < 20 years (pre-shutdown level)	117.36	168.70	0.487
Female, 20–30 years (pre-shutdown level)	35.22	185.61	0.850
Male, < 20 years (pre-shutdown level)	189.05	171.34	0.270
Male, 20–30 years (pre-shutdown level)	11.59	165.64	0.944
Female, < 20 years (pre-shutdown trend)	-34.50	76.48	0.652
Female, 20–30 years (pre-shutdown trend)	-31.33	84.84	0.712
Male, < 20 years (pre-shutdown trend)	-93.37	77.73	0.230
Male, 20–30 years (pre-shutdown trend)	-4.68	75.09	0.950
Female, < 20 years (first shutdown level)	-433.74	97.01	< 0.001
Female, 20–30 years (first shutdown level)	-678.58	99.78	< 0.001
Male, < 20 years (first shutdown level)	-697.59	97.65	< 0.001
Male, 20–30 years (first shutdown level)	-416.23	95.89	< 0.001
Female, < 20 years (first shutdown trend)	39.83	18.42	0.031
Female, 20–30 years (first shutdown trend)	61.80	19.11	0.001
Male, < 20 years (first shutdown trend)	50.61	18.58	0.007
Male, 20–30 years (first shutdown trend)	27.23	18.14	0.134
Female, < 20 years (summer level)	2.30	58.71	0.969
Female, 20–30 years (summer level)	31.97	59.24	0.590
Male, < 20 years (summer level)	-94.47	58.83	0.109
Male, 20–30 years (summer level)	29.39	58.53	0.616
Female, < 20 years (summer trend)	3.53	3.87	0.362
Female, 20–30 years (summer trend)	0.14	3.92	0.971
Male, < 20 years (summer trend)	3.52	3.88	0.364
Male, 20–30 years (summer trend)	-0.34	3.85	0.929
Female, < 20 years (second shutdown level)	-26.48	81.72	0.746
Female, 20–30 years (second shutdown level)	-43.16	83.32	0.605
Male, < 20 years (second shutdown level)	-228.20	82.07	0.006
Male, 20–30 years (second shutdown level)	-93.40	81.16	0.250
Female, < 20 years (second shutdown trend)	17.05	11.47	0.138
Female, 20–30 years (second shutdown trend)	18.74	11.70	0.110
Male, < 20 years (second shutdown trend)	23.56	11.52	0.041
Male, 20–30 years (second shutdown trend)	17.32	11.39	0.129

Teleconsultations

Variable	Coefficient estimate	Standard error	p-Value
Intercept	103.25	2.48	< 0.001
Ascension Day	-14.59	5.97	0.015
Spring/Easter break	-29.08	4.44	< 0.001
Autumn holidays	-17.89	4.82	< 0.001
Midsummer holidays	-53.28	4.82	< 0.001
New Year's holidays	-110.53	3.61	< 0.001
Pentecost	-19.38	5.85	0.001
Summer holidays	-36.79	3.81	< 0.001
Christmas holidays	14.37	7.04	0.042
Female, 20–30 years (overall level)	106.00	6.45	< 0.001
Male, < 20 years (overall level)	27.47	3.46	< 0.001
Male, 20–30 years (overall level)	45.48	4.32	< 0.001
Female, < 20 years (overall trend)	-0.07	0.04	0.049
Female, 20–30 years (overall trend)	-0.20	0.09	0.026
Male, < 20 years (overall trend)	-0.07	0.04	0.057
Male, 20–30 years (overall trend)	-0.04	0.05	0.466
Female, < 20 years (pre-shutdown level)	-13.24	21.63	0.541
Female, 20–30 years (pre-shutdown level)	-14.92	52.83	0.778
Male, < 20 years (pre-shutdown level)	0.38	26.83	0.989
Male, 20–30 years (pre-shutdown level)	-26.81	29.09	0.357
Female, < 20 years (pre-shutdown trend)	9.84	10.43	0.346
Female, 20–30 years (pre-shutdown trend)	22.33	25.44	0.380
Male, < 20 years (pre-shutdown trend)	9.63	12.83	0.453
Male, 20–30 years (pre-shutdown trend)	14.91	13.81	0.281
Female, < 20 years (first shutdown level)	219.99	22.96	< 0.001
Female, 20–30 years (first shutdown level)	515.74	55.73	< 0.001
Male, < 20 years (first shutdown level)	204.70	22.36	< 0.001
Male, 20–30 years (first shutdown level)	264.60	27.71	< 0.001
Female, < 20 years (first shutdown trend)	-5.25	4.36	0.229
Female, 20–30 years (first shutdown trend)	-3.57	10.66	0.738
Male, < 20 years (first shutdown trend)	-2.86	4.27	0.503
Male, 20–30 years (first shutdown trend)	-7.79	5.20	0.134
Female, < 20 years (summer level)	34.68	8.69	< 0.001
Female, 20–30 years (summer level)	111.20	19.08	< 0.001
Male, < 20 years (summer level)	27.14	8.56	0.002
Male, 20–30 years (summer level)	59.95	11.20	< 0.001
Female, < 20 years (summer trend)	-0.67	0.60	0.270
Female, 20–30 years (summer trend)	-4.09	1.27	0.001
Male, < 20 years (summer trend)	-0.68	0.58	0.243
Male, 20–30 years (summer trend)	-3.05	0.72	< 0.001
Female, < 20 years (second shutdown level)	90.14	12.95	< 0.001
Female, 20–30 years (second shutdown level)	129.99	25.92	< 0.001
Male, < 20 years (second shutdown level)	17.89	12.21	0.144
Male, 20–30 years (second shutdown level)	17.74	14.60	0.225
Female, < 20 years (second shutdown trend)	-6.30	1.35	< 0.001
Female, 20–30 years (second shutdown trend)	-7.27	2.98	0.015
Male, < 20 years (second shutdown trend)	0.22	1.28	0.862
Male, 20–30 years (second shutdown trend)	2.68	1.76	0.128

First consultations

Variable	Coefficient estimate	Standard erro	p-Value
Intercept	28.91	1.30	< 0.001
Ascension Day	-9.13	2.47	< 0.001
Spring/Easter break	-12.12	1.85	< 0.001
Autumn holidays	-3.10	1.85	0.095
Midsummer holidays	-19.07	2.50	< 0.001
New Year's holidays	-37.59	1.83	< 0.001
Pentecost	-3.90	2.45	0.112
Summer holidays	-10.76	1.48	< 0.001
Christmas holidays	-5.42	2.51	0.031
Female, 20–30 years (overall level)	60.06	2.80	< 0.001
Male, < 20 years (overall level)	6.21	1.83	< 0.001
Male, 20–30 years (overall level)	43.26	2.34	< 0.001
Female, < 20 years (overall trend)	0.06	0.02	0.006
Female, 20–30 years (overall trend)	-0.02	0.04	0.615
Male, < 20 years (overall trend)	-0.02	0.02	0.270
Male, 20–30 years (overall trend)	-0.04	0.03	0.147
Female, < 20 years (pre-shutdown level)	9.22	10.06	0.359
Female, 20–30 years (pre-shutdown level)	39.33	20.11	0.051
Male, < 20 years (pre-shutdown level)	-20.69	10.30	0.045
Male, 20–30 years (pre-shutdown level)	-17.83	15.39	0.247
Female, < 20 years (pre-shutdown trend)	-6.62	4.58	0.149
Female, 20–30 years (pre-shutdown trend)	-11.18	9.24	0.227
Male, < 20 years (pre-shutdown trend)	10.86	4.69	0.021
Male, 20–30 years (pre-shutdown trend)	5.56	7.02	0.428
Female, < 20 years (first shutdown level)	-20.49	5.51	< 0.001
Female, 20–30 years (first shutdown level)	-26.78	10.50	0.011
Male, < 20 years (first shutdown level)	-15.38	5.66	0.007
Male, 20–30 years (first shutdown level)	-28.31	8.40	< 0.001
Female, < 20 years (first shutdown trend)	1.21	1.03	0.241
Female, 20–30 years (first shutdown trend)	1.33	2.02	0.510
Male, < 20 years (first shutdown trend)	0.98	1.06	0.355
Male, 20–30 years (first shutdown trend)	1.70	1.57	0.280
Female, < 20 years (summer level)	-6.18	3.60	0.086
Female, 20–30 years (summer level)	-9.22	6.34	0.147
Male, < 20 years (summer level)	1.39	3.69	0.707
Male, 20–30 years (summer level)	11.83	5.48	0.031
Female, < 20 years (summer trend)	0.22	0.23	0.338
Female, 20–30 years (summer trend)	0.40	0.42	0.340
Male, < 20 years (summer trend)	-0.15	0.24	0.535
Male, 20–30 years (summer trend)	-0.68	0.35	0.056
Female, < 20 years (second shutdown level)	0.91	4.81	0.850
Female, 20–30 years (second shutdown level)	25.54	9.24	0.006
Male, < 20 years (second shutdown level)	-4.13	4.93	0.403
Male, 20–30 years (second shutdown level)	-10.85	7.29	0.137
Female, < 20 years (second shutdown trend)	0.07	0.67	0.919
Female, 20–30 years (second shutdown trend)	-3.06	1.26	0.016
Male, < 20 years (second shutdown trend)	0.88	0.68	0.199
Male, 20–30 years (second shutdown trend)	0.57	1.00	0.572

Further consultations

Variable	Coefficient estimate	Standard error	p-Value
Intercept	483.13	12.51	< 0.001
Ascension Day	-171.37	20.25	< 0.001
Spring/Easter break	-201.59	17.14	< 0.001
Autumn holidays	-19.83	22.87	0.386
Midsummer holidays	-136.50	34.50	< 0.001
New Year's holidays	-638.04	26.46	< 0.001
Pentecost	-141.00	20.06	< 0.001
Summer holidays	-66.80	25.08	0.008
Christmas holidays	37.85	28.28	0.181
Female, 20–30 years (overall level)	807.97	17.17	< 0.001
Male, < 20 years (overall level)	82.91	17.16	< 0.001
Male, 20–30 years (overall level)	542.64	17.15	< 0.001
Female, < 20 years (overall trend)	-0.08	0.19	0.670
Female, 20–30 years (overall trend)	-0.06	0.19	0.741
Male, < 20 years (overall trend)	-0.21	0.19	0.261
Male, 20–30 years (overall trend)	-0.68	0.19	< 0.001
Female, < 20 years (pre-shutdown level)	41.71	99.65	0.676
Female, 20–30 years (pre-shutdown level)	-68.96	108.96	0.527
Male, < 20 years (pre-shutdown level)	79.28	101.19	0.434
Male, 20–30 years (pre-shutdown level)	13.09	99.99	0.896
Female, < 20 years (pre-shutdown trend)	-4.94	45.17	0.913
Female, 20–30 years (pre-shutdown trend)	29.40	49.74	0.555
Male, < 20 years (pre-shutdown trend)	-30.03	45.89	0.513
Male, 20–30 years (pre-shutdown trend)	5.96	45.33	0.895
Female, < 20 years (first shutdown level)	-67.37	57.47	0.242
Female, 20–30 years (first shutdown level)	-74.33	59.13	0.209
Male, < 20 years (first shutdown level)	-195.19	57.90	< 0.001
Male, 20–30 years (first shutdown level)	-34.99	57.58	0.544
Female, < 20 years (first shutdown trend)	15.67	10.90	0.151
Female, 20–30 years (first shutdown trend)	34.35	11.32	0.002
Male, < 20 years (first shutdown trend)	24.70	11.01	0.025
Male, 20–30 years (first shutdown trend)	10.68	10.93	0.329
Female, < 20 years (summer level)	41.92	34.87	0.230
Female, 20–30 years (summer level)	106.08	35.18	0.003
Male, < 20 years (summer level)	-13.17	34.94	0.706
Male, 20–30 years (summer level)	45.34	34.88	0.194
Female, < 20 years (summer trend)	0.75	2.30	0.743
Female, 20–30 years (summer trend)	-2.43	2.33	0.297
Male, < 20 years (summer trend)	0.89	2.30	0.698
Male, 20–30 years (summer trend)	0.02	2.30	0.994
Female, < 20 years (second shutdown level)	54.58	48.47	0.261
Female, 20–30 years (second shutdown level)	39.73	49.42	0.422
Male, < 20 years (second shutdown level)	-79.79	48.70	0.102
Male, 20–30 years (second shutdown level)	7.96	48.53	0.870
Female, < 20 years (second shutdown trend)	4.90	6.80	0.472
Female, 20–30 years (second shutdown trend)	8.73	6.94	0.209
Male, < 20 years (second shutdown trend)	11.15	6.83	0.103
Male, 20–30 years (second shutdown trend)	8.21	6.81	0.229

Total psychotropic medication

Variable	Coefficient estimate	Standard error	p-Value
Intercept	168.37	8.75	< 0.001
Ascension Day	-74.52	14.55	< 0.001
Spring/Easter break	-49.11	11.57	< 0.001
Autumn holidays	-25.07	13.23	0.059
Midsummer holidays	-106.70	16.96	< 0.001
New Year's holidays	-178.10	13.49	< 0.001
Pentecost	-66.86	14.42	< 0.001
Summer holidays	-51.60	10.42	< 0.001
Christmas holidays	121.12	16.93	< 0.001
Female, 20–30 years (overall level)	804.16	12.53	< 0.001
Male, < 20 years (overall level)	88.57	12.49	< 0.001
Male, 20–30 years (overall level)	720.38	12.52	< 0.001
Female, < 20 years (overall trend)	0.13	0.13	0.317
Female, 20–30 years (overall trend)	-0.21	0.14	0.140
Male, < 20 years (overall trend)	0.34	0.14	0.015
Male, 20–30 years (overall trend)	-0.77	0.14	< 0.001
Female, < 20 years (pre-shutdown level)	-36.54	69.82	0.601
Female, 20–30 years (pre-shutdown level)	33.98	80.23	0.672
Male, < 20 years (pre-shutdown level)	-10.62	71.23	0.881
Male, 20–30 years (pre-shutdown level)	87.13	76.06	0.252
Female, < 20 years (pre-shutdown trend)	16.57	32.07	0.606
Female, 20–30 years (pre-shutdown trend)	3.43	36.57	0.925
Male, < 20 years (pre-shutdown trend)	13.04	32.37	0.687
Male, 20–30 years (pre-shutdown trend)	7.31	34.51	0.832
Female, < 20 years (first shutdown level)	-24.03	36.47	0.510
Female, 20–30 years (first shutdown level)	17.64	43.96	0.688
Male, < 20 years (first shutdown level)	-19.75	40.31	0.624
Male, 20–30 years (first shutdown level)	23.79	43.30	0.583
Female, < 20 years (first shutdown trend)	-2.74	7.02	0.697
Female, 20–30 years (first shutdown trend)	-13.97	8.40	0.097
Male, < 20 years (first shutdown trend)	-8.20	7.55	0.278
Male, 20–30 years (first shutdown trend)	-8.85	8.24	0.283
Female, < 20 years (summer level)	-0.69	22.36	0.976
Female, 20–30 years (summer level)	-24.26	26.22	0.355
Male, < 20 years (summer level)	-32.60	25.63	0.204
Male, 20–30 years (summer level)	32.02	26.09	0.220
Female, < 20 years (summer trend)	-0.01	1.47	0.992
Female, 20–30 years (summer trend)	4.49	1.73	0.010
Male, < 20 years (summer trend)	0.84	1.68	0.616
Male, 20–30 years (summer trend)	2.31	1.72	0.179
Female, < 20 years (second shutdown level)	2.51	32.53	0.939
Female, 20–30 years (second shutdown level)	27.14	36.79	0.461
Male, < 20 years (second shutdown level)	-10.31	34.80	0.767
Male, 20–30 years (second shutdown level)	62.27	36.39	0.088
Female, < 20 years (second shutdown trend)	10.34	4.52	0.023
Female, 20–30 years (second shutdown trend)	5.86	5.17	0.257
Male, < 20 years (second shutdown trend)	0.36	4.84	0.940
Male, 20–30 years (second shutdown trend)	1.97	5.10	0.700

Antidepressants

Variable	Coefficient estimate	Standard error	p-Value
Intercept	36.59	1.48	< 0.001
Ascension Day	-4.87	2.68	0.070
Spring/Easter break	-2.13	2.01	0.290
Autumn holidays	-1.46	2.02	0.470
Midsummer holidays	-11.76	2.69	< 0.001
New Year's holidays	-17.20	1.99	< 0.001
Pentecost	-7.51	2.66	0.005
Summer holidays	-5.12	1.51	< 0.001
Christmas holidays	21.75	2.75	< 0.001
Female, 20–30 years (overall level)	379.61	10.68	< 0.001
Male, < 20 years (overall level)	-15.82	1.82	< 0.001
Male, 20–30 years (overall level)	245.82	5.20	< 0.001
Female, < 20 years (overall trend)	0.07	0.02	0.003
Female, 20–30 years (overall trend)	0.19	0.16	0.235
Male, < 20 years (overall trend)	0.03	0.02	0.077
Male, 20–30 years (overall trend)	-0.33	0.08	< 0.001
Female, < 20 years (pre-shutdown level)	-16.12	11.59	0.165
Female, 20–30 years (pre-shutdown level)	15.24	85.51	0.859
Male, < 20 years (pre-shutdown level)	-11.08	8.64	0.200
Male, 20–30 years (pre-shutdown level)	68.41	41.72	0.102
Female, < 20 years (pre-shutdown trend)	9.18	5.27	0.082
Female, 20–30 years (pre-shutdown trend)	3.17	39.29	0.936
Male, < 20 years (pre-shutdown trend)	5.64	3.97	0.155
Male, 20–30 years (pre-shutdown trend)	-7.15	18.93	0.706
Female, < 20 years (first shutdown level)	-2.23	6.56	0.735
Female, 20–30 years (first shutdown level)	25.03	44.58	0.575
Male, < 20 years (first shutdown level)	5.00	4.51	0.268
Male, 20–30 years (first shutdown level)	36.94	23.77	0.121
Female, < 20 years (first shutdown trend)	-0.86	1.23	0.484
Female, 20–30 years (first shutdown trend)	-14.49	8.57	0.092
Male, < 20 years (first shutdown trend)	-1.23	0.87	0.158
Male, 20–30 years (first shutdown trend)	-7.78	4.52	0.086
Female, < 20 years (summer level)	-1.99	4.17	0.633
Female, 20–30 years (summer level)	-22.07	26.81	0.411
Male, < 20 years (summer level)	-0.75	2.81	0.790
Male, 20–30 years (summer level)	23.70	14.35	0.099
Female, < 20 years (summer trend)	0.34	0.27	0.211
Female, 20–30 years (summer trend)	0.73	1.76	0.679
Male, < 20 years (summer trend)	-0.11	0.18	0.549
Male, 20–30 years (summer trend)	1.69	0.95	0.076
Female, < 20 years (second shutdown level)	11.87	5.66	0.036
Female, 20–30 years (second shutdown level)	37.98	39.23	0.333
Male, < 20 years (second shutdown level)	-0.13	4.05	0.974
Male, 20–30 years (second shutdown level)	42.72	20.02	0.033
Female, < 20 years (second shutdown trend)	1.47	0.79	0.063
Female, 20–30 years (second shutdown trend)	-1.62	5.31	0.761
Male, < 20 years (second shutdown trend)	0.76	0.57	0.180
Male, 20–30 years (second shutdown trend)	-0.89	2.81	0.751

Anxiolytics

Variable	Coefficient estimate	Standard error	p-Value
Intercept	20.47	0.91	< 0.001
Ascension Day	-2.90	1.86	0.119
Spring/Easter break	-4.39	1.36	0.001
Autumn holidays	-1.75	1.35	0.197
Midsummer holidays	-6.98	1.82	< 0.001
New Year's holidays	-9.39	1.32	< 0.001
Pentecost	-4.18	1.85	0.024
Summer holidays	-3.57	0.96	< 0.001
Christmas holidays	9.83	1.88	< 0.001
Female, 20–30 years (overall level)	147.48	4.34	< 0.001
Male, < 20 years (overall level)	-4.45	1.22	< 0.001
Male, 20–30 years (overall level)	133.75	3.65	< 0.001
Female, < 20 years (overall trend)	-0.01	0.01	0.633
Female, 20–30 years (overall trend)	-0.10	0.07	0.139
Male, < 20 years (overall trend)	0.04	0.01	0.001
Male, 20–30 years (overall trend)	-0.08	0.05	0.141
Female, < 20 years (pre-shutdown level)	2.54	7.27	0.727
Female, 20–30 years (pre-shutdown level)	-24.01	33.55	0.474
Male, < 20 years (pre-shutdown level)	-2.53	6.63	0.703
Male, 20–30 years (pre-shutdown level)	16.58	28.01	0.554
Female, < 20 years (pre-shutdown trend)	-1.28	3.34	0.702
Female, 20–30 years (pre-shutdown trend)	23.20	15.35	0.131
Male, < 20 years (pre-shutdown trend)	1.98	3.04	0.515
Male, 20–30 years (pre-shutdown trend)	-2.46	12.82	0.848
Female, < 20 years (first shutdown level)	-3.40	3.80	0.371
Female, 20–30 years (first shutdown level)	22.86	17.88	0.201
Male, < 20 years (first shutdown level)	-1.15	3.46	0.741
Male, 20–30 years (first shutdown level)	0.37	14.91	0.980
Female, < 20 years (first shutdown trend)	-0.66	0.73	0.366
Female, 20–30 years (first shutdown trend)	-1.88	3.38	0.578
Male, < 20 years (first shutdown trend)	0.17	0.67	0.801
Male, 20–30 years (first shutdown trend)	-0.81	2.82	0.773
Female, < 20 years (summer level)	-4.18	2.33	0.074
Female, 20–30 years (summer level)	12.95	11.48	0.260
Male, < 20 years (summer level)	-3.49	2.14	0.103
Male, 20–30 years (summer level)	5.22	9.55	0.585
Female, < 20 years (summer trend)	0.21	0.15	0.164
Female, 20–30 years (summer trend)	-0.38	0.74	0.610
Male, < 20 years (summer trend)	0.02	0.14	0.884
Male, 20–30 years (summer trend)	0.35	0.61	0.568
Female, < 20 years (second shutdown level)	3.96	3.38	0.242
Female, 20–30 years (second shutdown level)	19.75	15.47	0.202
Male, < 20 years (second shutdown level)	-5.49	3.09	0.076
Male, 20–30 years (second shutdown level)	37.85	12.92	0.004
Female, < 20 years (second shutdown trend)	0.40	0.47	0.395
Female, 20–30 years (second shutdown trend)	-1.17	2.11	0.578
Male, < 20 years (second shutdown trend)	0.30	0.43	0.489
Male, 20–30 years (second shutdown trend)	-1.88	1.76	0.287

Antipsychotics

Variable	Coefficient estimate	Standard error	p-Value
Intercept	22.88	0.97	< 0.001
Ascension Day	-6.87	2.35	0.004
Spring/Easter break	-5.52	1.78	0.002
Autumn holidays	-2.70	1.81	0.136
Midsummer holidays	-9.07	2.45	< 0.001
New Year's holidays	-17.76	1.80	< 0.001
Pentecost	-5.48	2.33	0.019
Summer holidays	-6.06	1.52	< 0.001
Christmas holidays	13.69	2.41	< 0.001
Female, 20–30 years (overall level)	151.60	3.80	< 0.001
Male, < 20 years (overall level)	11.66	1.62	< 0.001
Male, 20–30 years (overall level)	243.90	4.96	< 0.001
Female, < 20 years (overall trend)	-0.01	0.01	0.572
Female, 20–30 years (overall trend)	-0.19	0.06	< 0.001
Male, < 20 years (overall trend)	0.07	0.02	0.001
Male, 20–30 years (overall trend)	-0.21	0.08	0.005
Female, < 20 years (pre-shutdown level)	15.49	7.43	0.038
Female, 20–30 years (pre-shutdown level)	3.91	28.90	0.892
Male, < 20 years (pre-shutdown level)	-2.46	10.36	0.812
Male, 20–30 years (pre-shutdown level)	12.45	39.43	0.752
Female, < 20 years (pre-shutdown trend)	-3.61	3.38	0.286
Female, 20–30 years (pre-shutdown trend)	-1.55	13.16	0.906
Male, < 20 years (pre-shutdown trend)	2.56	4.71	0.587
Male, 20–30 years (pre-shutdown trend)	4.43	17.88	0.804
Female, < 20 years (first shutdown level)	7.78	4.13	0.060
Female, 20–30 years (first shutdown level)	8.92	15.93	0.576
Male, < 20 years (first shutdown level)	8.71	5.76	0.131
Male, 20–30 years (first shutdown level)	34.74	22.79	0.128
Female, < 20 years (first shutdown trend)	-1.07	0.77	0.169
Female, 20–30 years (first shutdown trend)	0.02	2.98	0.996
Male, < 20 years (first shutdown trend)	-1.31	1.08	0.223
Male, 20–30 years (first shutdown trend)	-4.67	4.30	0.278
Female, < 20 years (summer level)	3.95	2.68	0.140
Female, 20–30 years (summer level)	25.69	10.34	0.013
Male, < 20 years (summer level)	-2.48	3.72	0.506
Male, 20–30 years (summer level)	7.89	13.97	0.573
Female, < 20 years (summer trend)	0.25	0.17	0.146
Female, 20–30 years (summer trend)	0.49	0.67	0.466
Male, < 20 years (summer trend)	0.14	0.24	0.573
Male, 20–30 years (summer trend)	-0.11	0.92	0.902
Female, < 20 years (second shutdown level)	8.25	3.61	0.023
Female, 20–30 years (second shutdown level)	48.76	13.75	< 0.001
Male, < 20 years (second shutdown level)	-10.48	5.00	0.037
Male, 20–30 years (second shutdown level)	14.70	19.31	0.447
Female, < 20 years (second shutdown trend)	0.68	0.50	0.177
Female, 20–30 years (second shutdown trend)	-1.52	1.89	0.422
Male, < 20 years (second shutdown trend)	0.94	0.69	0.176
Male, 20–30 years (second shutdown trend)	-0.87	2.70	0.747
