# Supplementary material for: The Impact of COVID-19 on Mental Healthcare Utilization in Switzerland Was Strongest Among Young Females—Retrospective Study in 2018–2020
Source: Int J Public Health. 2023 May 19;68:1605839. doi: 10.3389/ijph.2023.1605839 (PMC10235482; doi:10.3389/ijph.2023.1605839)
Supplement: Supplementary file 3 [file DataSheet1.PDF]

International Journal of Public Health

"The impact of COVID-19 on mental health care utilization in Switzerland was strongest among young females – retrospective study in 2018-2020"

Supplementary File 1

Definitions of psychotherapy consultations

Variables	TARMED positions
All psychotherapy consultations	02.0010 | 02.0020 | 02.0030 | 02.0040 | 02.0050 | 02.0060 | 02.0065 | 02.0066 | 02.0210 | 02.0220 | 02.0230 | 02.0240 | 02.0250
Face-to-face consultations	02.0010 | 02.0020 | 02.0030 | 02.0040 | 02.0050 | 02.0210 | 02.0220 | 02.0230 | 02.0240
Teleconsultations	02.0060 | 02.0065 | 02.0066 |02.0250
First consultations (only psychiatrists)	02.0010
Further consultation (only psychiatrists)	02.0020 | 02.0030 | 02.0040 | 02.0050 | 02.0060 | 02.0065 | 02.0066

Age categories

Combined age category	Data source 1 (MedStat)	Data source 2 (Helsana)
<20	0-19	0-18
20-30	20-29	19-30
30-40	30-39	31-40
40-50	40-49	41-50
50-60	50-59	51-60
60-70	60-69	61-70
70-80	70-79	71-80
80+	80+	81+

Categories are given in years.

Model diagnostics

Residuals of the fitted models were checked for autocorrelation using residual autocorrelation (ACF) and partial residual autocorrelation function (PACF) plots. We further assessed residuals with Tukey-Anscombe plots. If they suggested heteroscedasticity, we re-fitted the corresponding model using a residual variance structure in form of a power law of the observed response as implemented by the varPower() function of the R package nlme (1). In the subgroup models, relevant heteroscedasticity was accounted for with a stratum-wise constant residual variance as implemented by the varIdent() function of the R package nlme or a stratum-wise power law of the observed response as appropriate. The chosen variance structures are summarized in the two tables below, where “y” indicates the respective outcome variable and “| stratum” indicates that stratum-specific terms were used. “Identity” denotes that no specific adjustment for heteroscedasticity was deemed necessary.

Variance structures chosen for models of the inpatient outcomes

**Overall models**	
**Outcome**	**Variance structure**
Total psychiatric admissions	varPower(~ y)
Affective disorders	varPower(~ y)
Neurotic disorders	varPower(~ y)
Psychotic disorders	varPower(~ y)
**Subgroup analyses**	
**Outcome**	**Variance structure**
Total psychiatric admissions	varIdent(~ 1 | stratum)

Affective disorders	varIdent(~ 1 | stratum)
Neurotic disorders	varIdent(~ 1 | stratum)
Psychotic disorders	varIdent(~ 1 | stratum)

Variance structures chosen for the models of the outpatient outcomes

**Overall models**	
**Outcome**	**Variance structure**
Total psychiatric consultations	varPower(~ y)
In-person consultations	varPower(~ y)
Teleconsultations	varPower(~ y)
First consultations	Identity
Further consultations	varPower(~ y)
Total psychotropic medication	Identity
Antidepressants	Identity
Anxiolytics	Identity
Antipsychotics	Identity
**Subgroup analyses**	
**Outcome**	**Variance structure**
Total psychiatric consultations	Identity
In-person consultations	Identity
Teleconsultations	varPower(~ y | stratum)
First consultations	varIdent(~ 1 | stratum)
Further consultations	Identity
Total psychotropic medication	Identity
Antidepressants	varIdent(~ 1|STRATUM)
Anxiolytics	varIdent(~ 1|STRATUM)
Antipsychotics	varIdent(~ 1|STRATUM)

Tukey-Anscombe plots, QQ residual plots, calibration plots, ACF and PACF plots of the final models are stored in the Supplementary Material 2.zip archive. The ACF and PACF plots suggested no relevant residual autocorrelation structure for any outcome. We therefore refrained from using more complex time series structures such as involving autoregressive or moving-average processes.

Computation of effect estimates

For each outcome and each pandemic period $p = 1,2,3,4$, we computed an absolute effect estimate as the difference between the predicted weekly incidences of the true COVID-19 scenario and the counterfactual scenario under absence of COVID-19 cumulated over the entire period. At week t, this difference amounted to $\hat{\beta}_p + \hat{\beta}_{tp}t$, where $\hat{\beta}_p$ and $\hat{\beta}_{tp}$ denote the coefficient estimates for the level and trend change due to the period p, respectively. The cumulated effect was obtained by integration of this difference over t from 1 to the length T_p of the period p:

$$\hat{E}_p = \hat{\beta}_p\left(T_p - 1\right) + \frac{1}{2}\hat{\beta}_{tp}(T_p^2 - 1).$$

Using the estimated variances $\hat{V}_p$ and $\hat{V}_{tp}$ of $\hat{\beta}_p$ and $\hat{\beta}_{tp}$, respectively, and their estimated covariance $\hat{C}_{p,tp}$, a variance estimate of $\hat{E}_p$ was computed as

$$\hat{V}_{E_p} = \left(T_p - 1\right)^2 \hat{V}_p + \frac{1}{4}\left(T_p^2 - 1\right)^2 \hat{V}_{tp} + (T_p - 1)(T_p^2 - 1)\hat{C}_{p,tp}.$$

Given a bivariate normal distribution for the least squares estimates $(\hat{\beta}_p, \hat{\beta}_{tp})$ centered at (β_p, β_{tp}), the absolute effect estimate $\hat{E}_p$ follows a univariate normal distribution of variance $\hat{V}_{E_p}$ centered at the true absolute effect $E_p = \beta_p\left(T_p - 1\right) + \frac{1}{2}\beta_{tp}(T_p^2 - 1)$. Accordingly, bounds of the reported 95% confidence intervals (CIs) for $\hat{E}_p$ were computed as

$$\hat{E}_p \pm z_{0.975}\sqrt{\hat{V}_{E_p}},$$

where $z_{0.975}$ denotes the 0.975-quantile of the standard normal distribution.

We computed corresponding relative effect estimates for the period p as the ratio of $\hat{E}_p$ to the predicted incidence proportion in the period p under the counterfactual scenario, obtained by cumulating the predicted weekly incidence proportion over the entire period p with the trapezoid rule. Note that the above expression for $\hat{E}_p$ is equal to the difference of the predicted weekly incidence proportions under the real and the counterfactual scenario cumulated over the period p with the trapezoid rule.

References

1. Pinheiro JC, DM B. Mixed-Effects Models in S and S-PLUS. New York: Springer-Verlag; 2000.
