# Supplementary figures and images for: The Impact of COVID-19 on Mental Healthcare Utilization in Switzerland Was Strongest Among Young Females—Retrospective Study in 2018–2020
Source: Int J Public Health. 2023 May 19;68:1605839. doi: 10.3389/ijph.2023.1605839 (PMC10235482; doi:10.3389/ijph.2023.1605839)

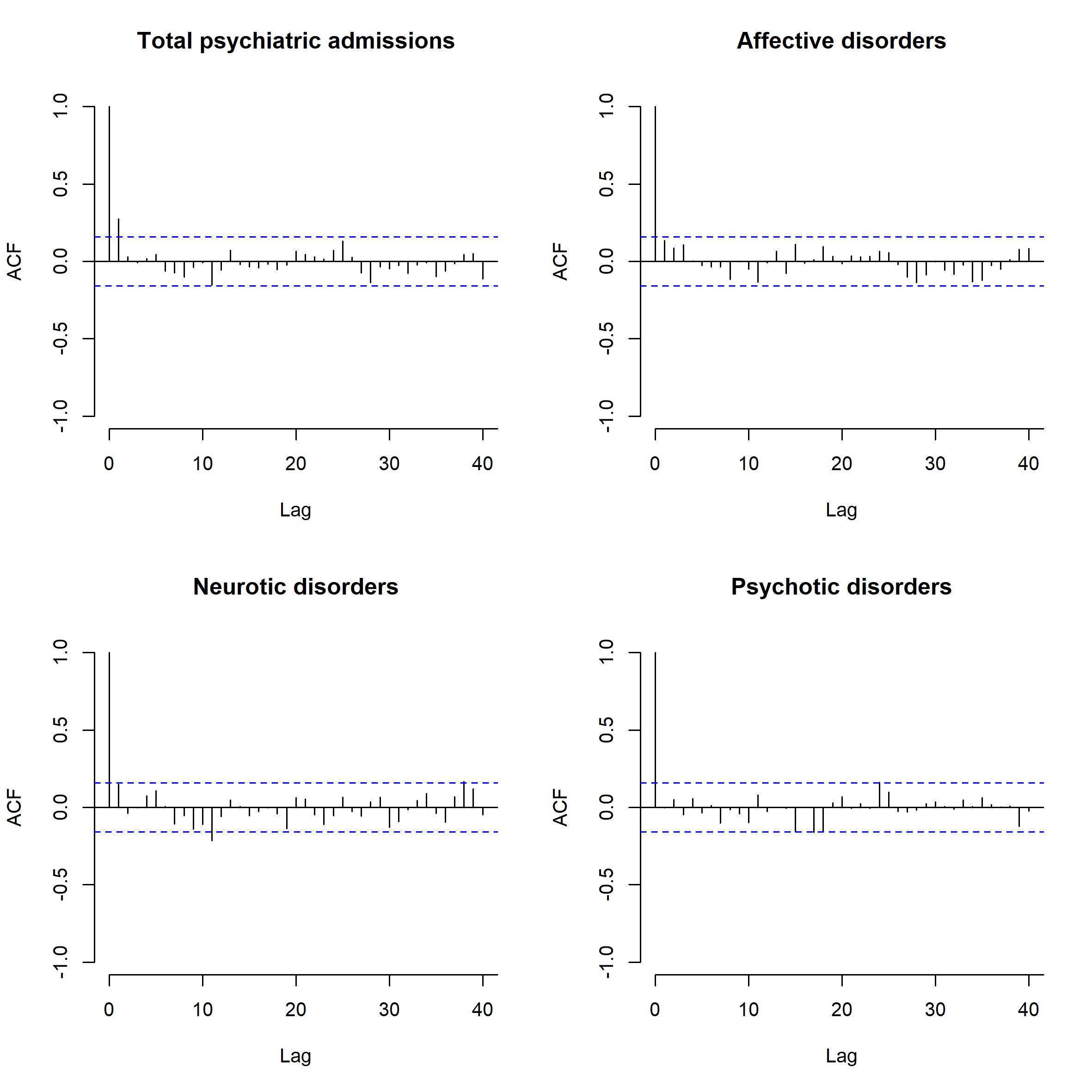

Supplement: Supplementary file 4 [file DataSheet2.ZIP › diagnostic_plots/Inpatient_Overall_ACF.tiff]

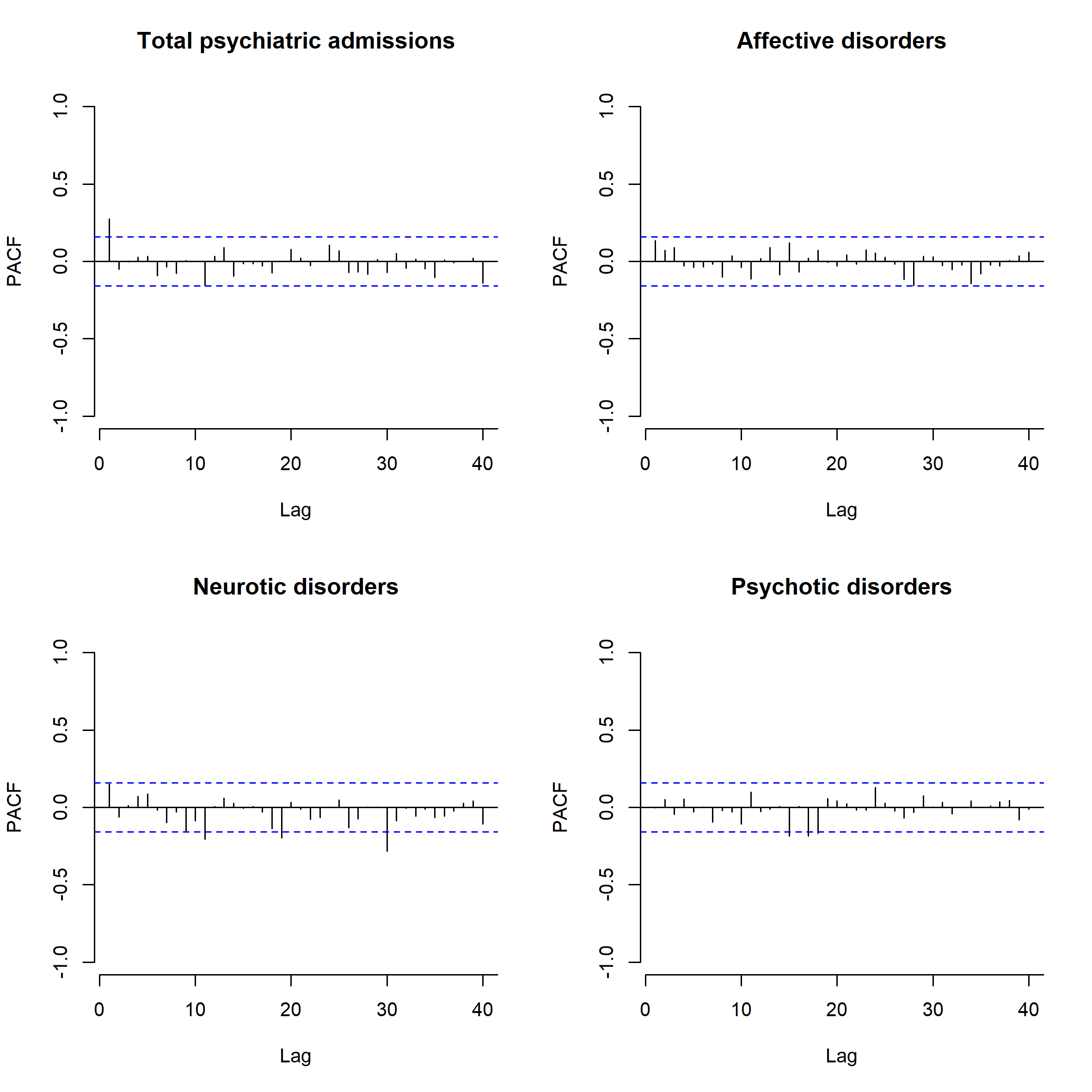

Supplement: Supplementary file 4 [file DataSheet2.ZIP › diagnostic_plots/Inpatient_Overall_PACFs.tiff]

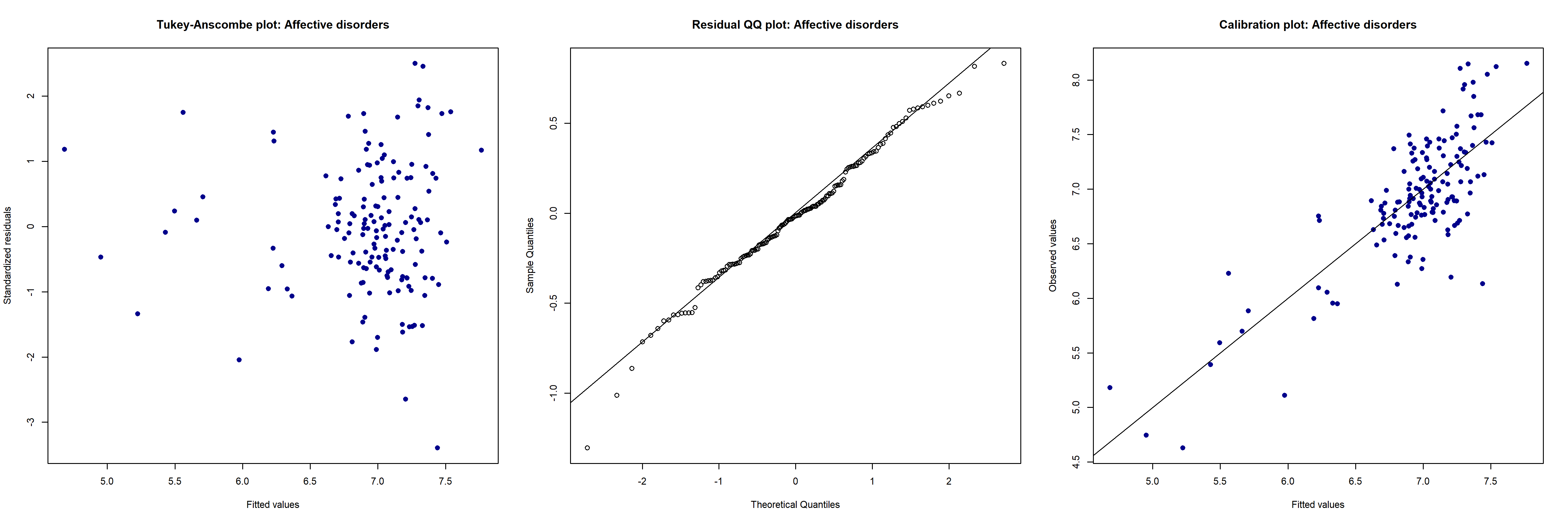

Supplement: Supplementary file 4 [file DataSheet2.ZIP › diagnostic_plots/Inpatient_Overall_Residuals_Affective disorders.tiff]

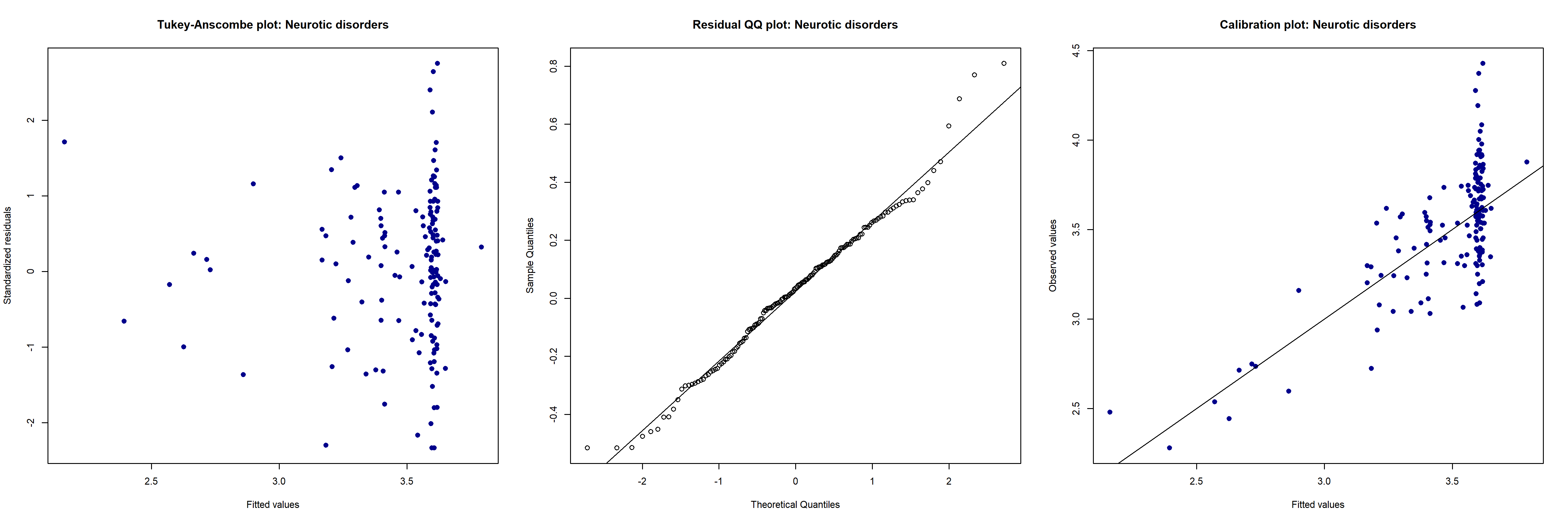

Supplement: Supplementary file 4 [file DataSheet2.ZIP › diagnostic_plots/Inpatient_Overall_Residuals_Neurotic disorders.tiff]

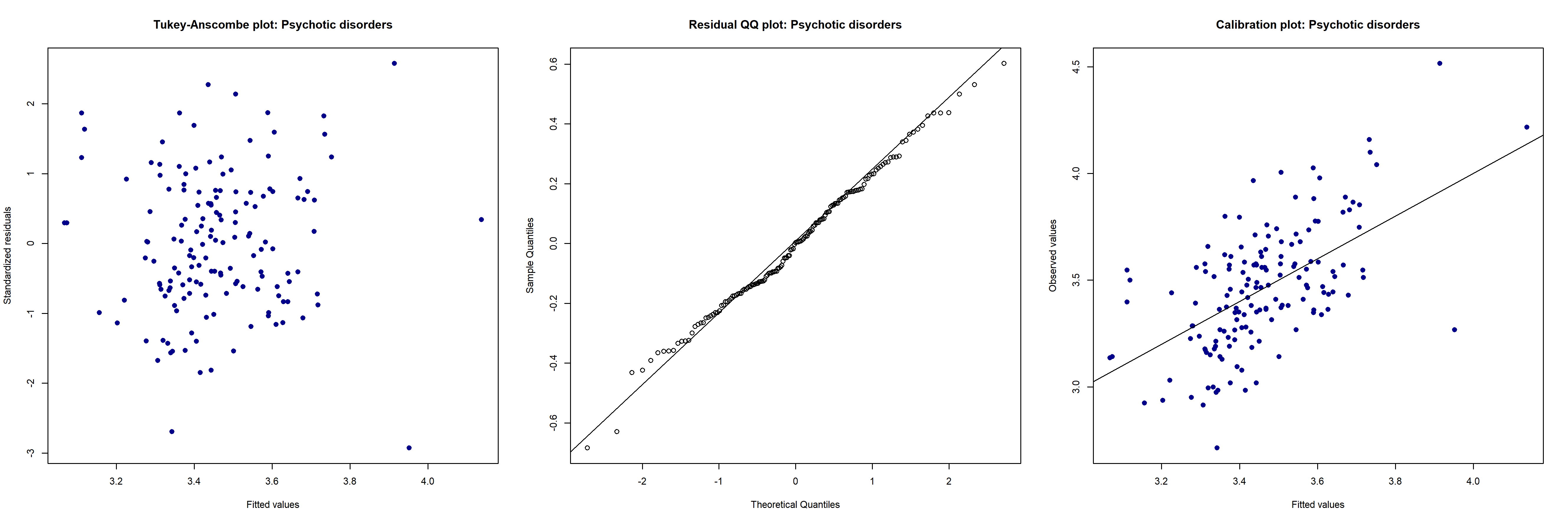

Supplement: Supplementary file 4 [file DataSheet2.ZIP › diagnostic_plots/Inpatient_Overall_Residuals_Psychotic disorders.tiff]

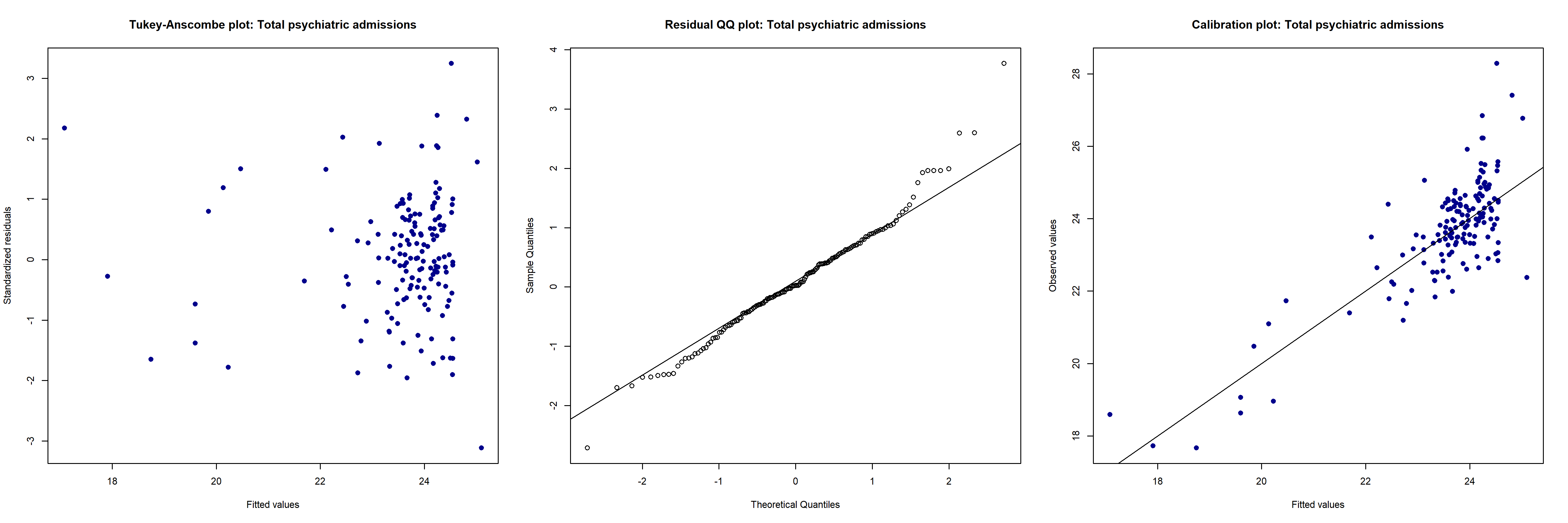

Supplement: Supplementary file 4 [file DataSheet2.ZIP › diagnostic_plots/Inpatient_Overall_Residuals_Total psychiatric admissions.tiff]

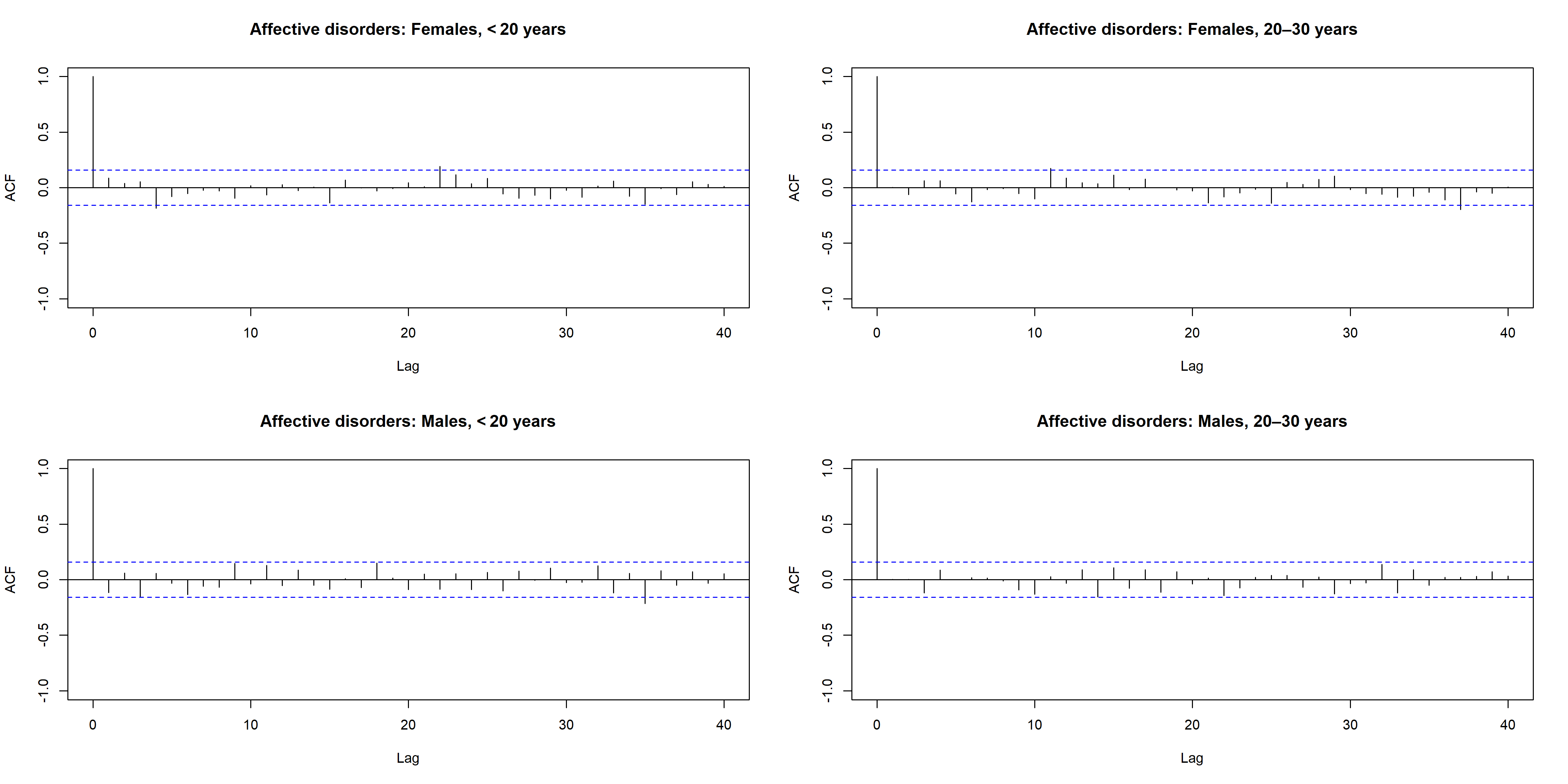

Supplement: Supplementary file 4 [file DataSheet2.ZIP › diagnostic_plots/Inpatient_Stratified_ACF_Affective disorders.tiff]

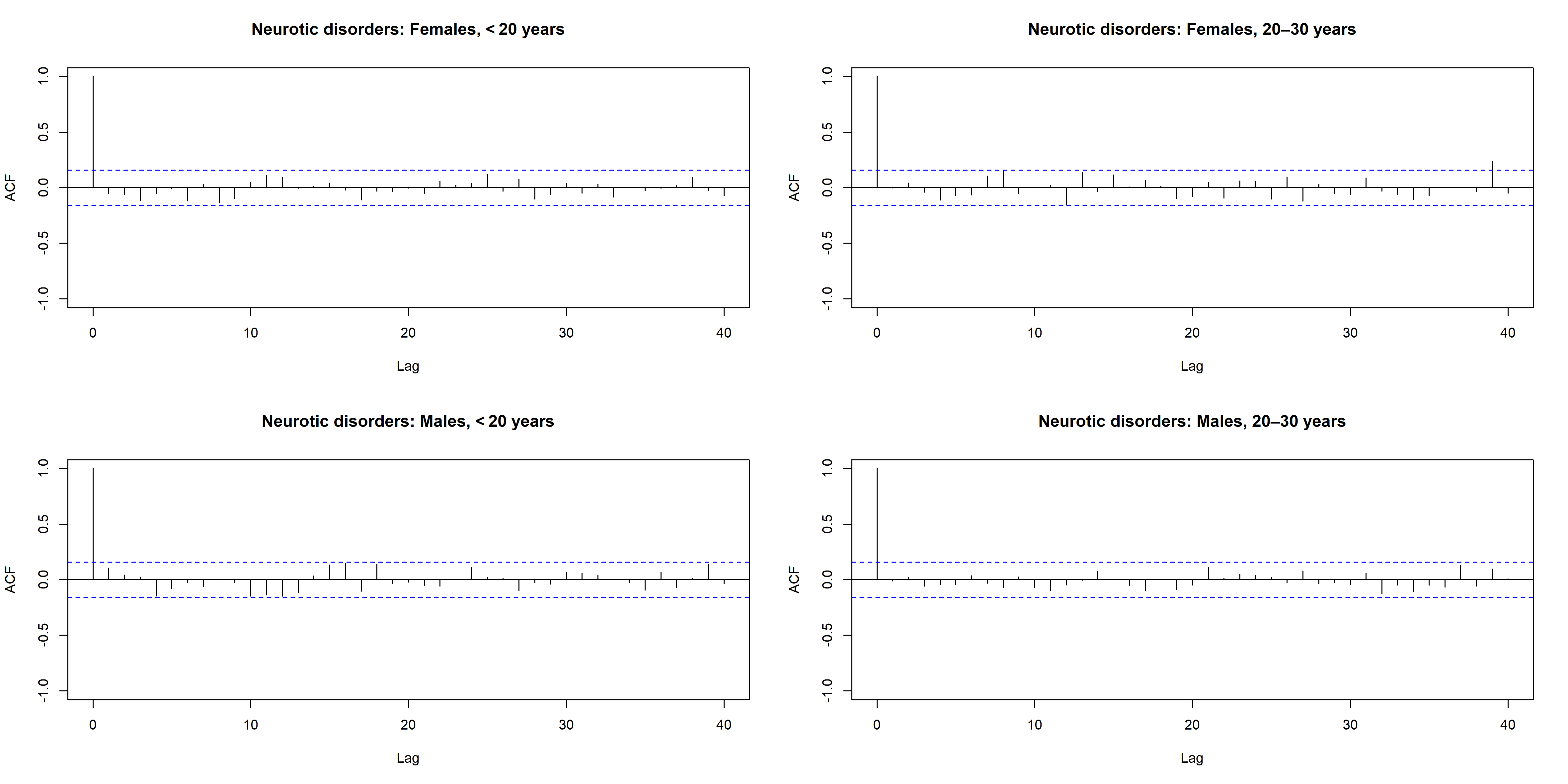

Supplement: Supplementary file 4 [file DataSheet2.ZIP › diagnostic_plots/Inpatient_Stratified_ACF_Neurotic disorders.tiff]

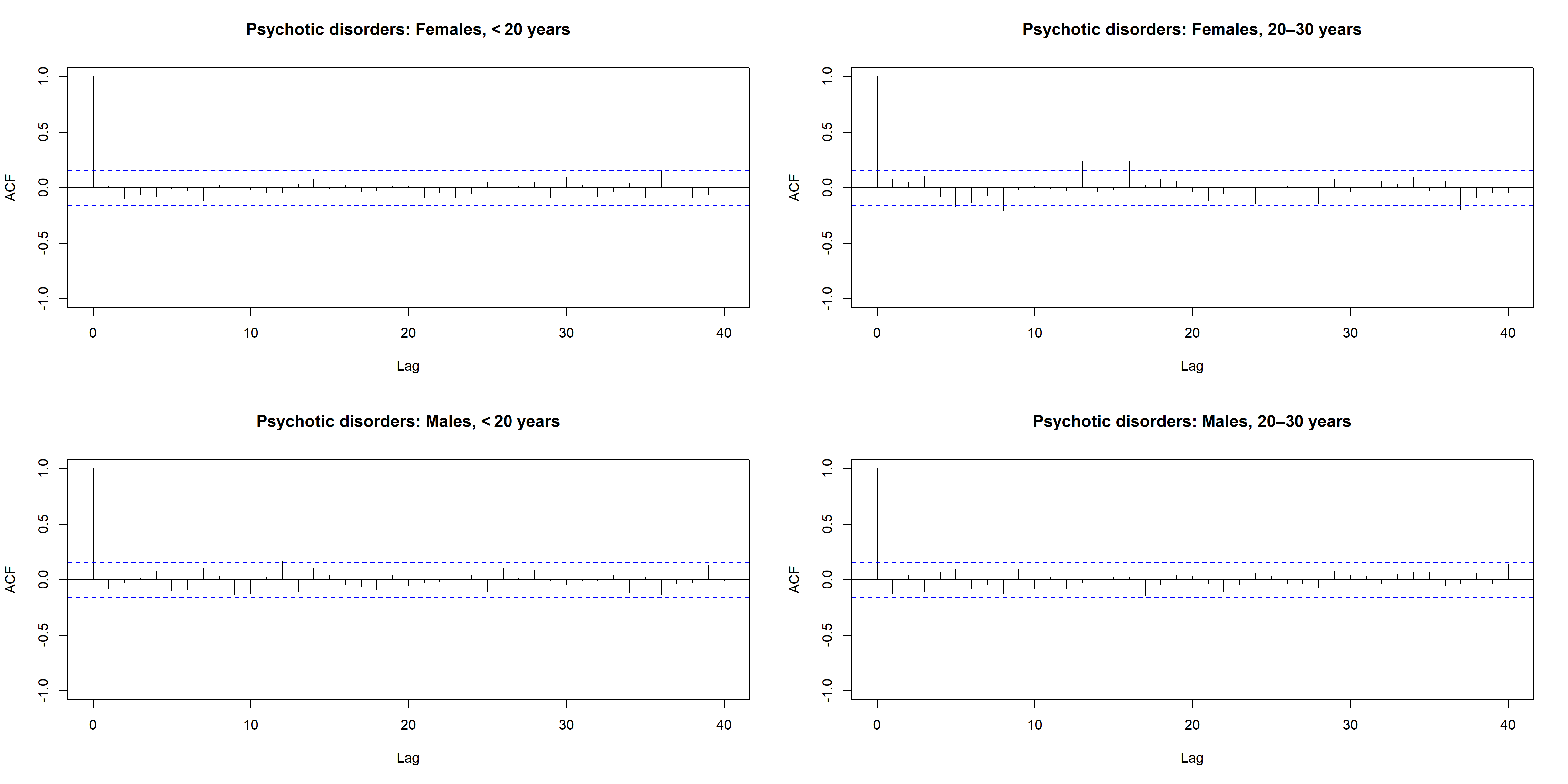

Supplement: Supplementary file 4 [file DataSheet2.ZIP › diagnostic_plots/Inpatient_Stratified_ACF_Psychotic disorders.tiff]

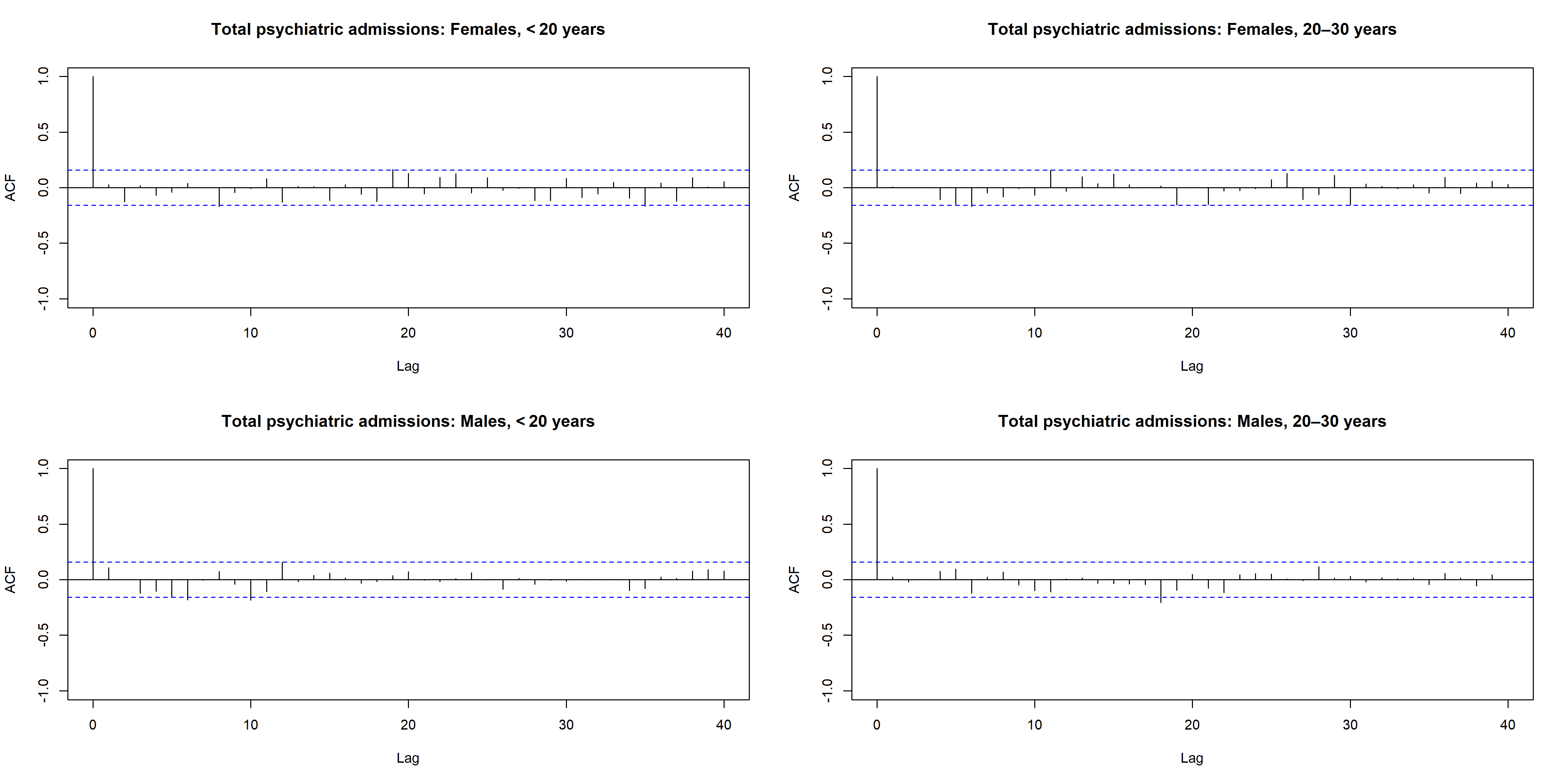

Supplement: Supplementary file 4 [file DataSheet2.ZIP › diagnostic_plots/Inpatient_Stratified_ACF_Total psychiatric admissions.tiff]

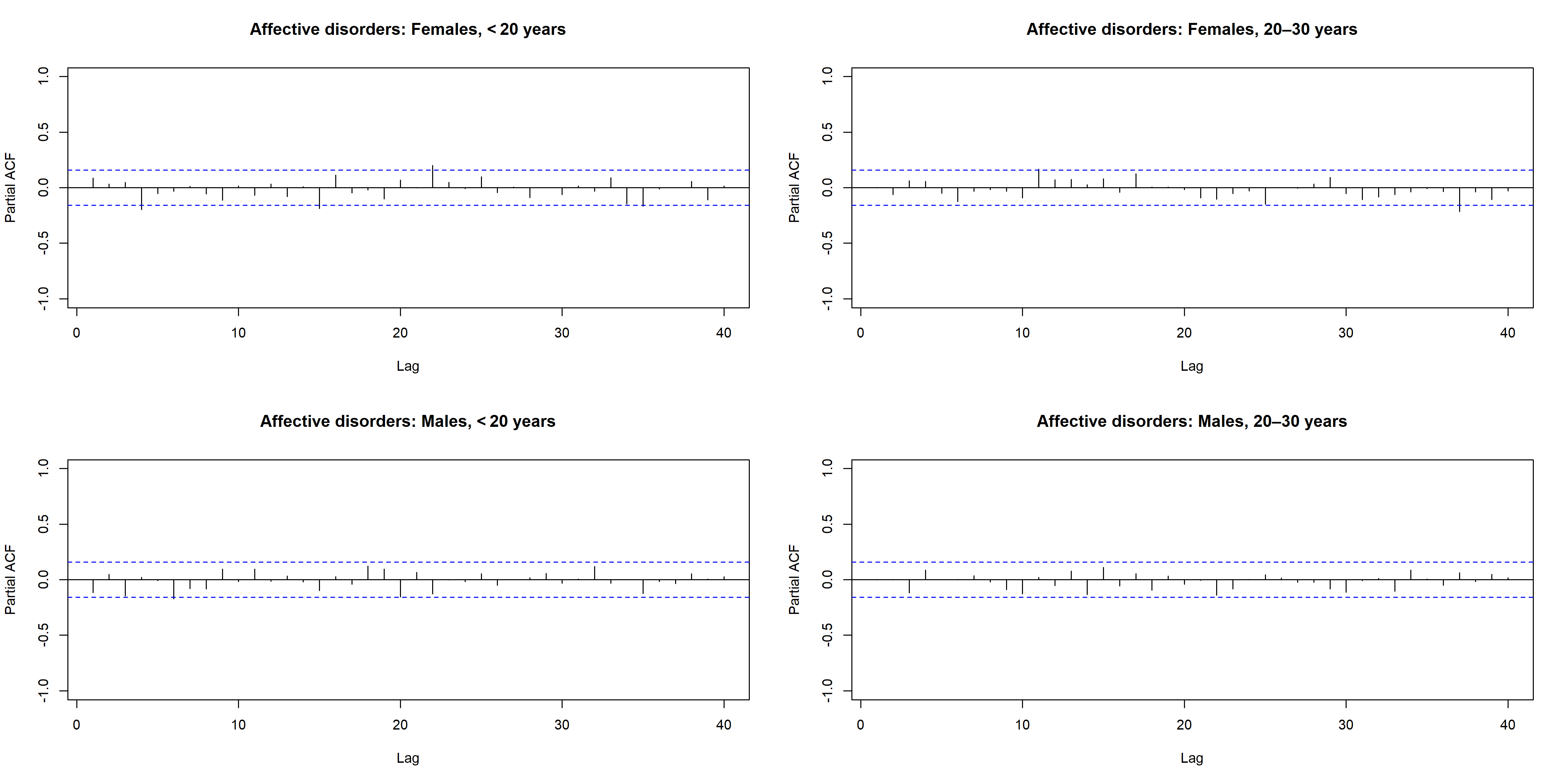

Supplement: Supplementary file 4 [file DataSheet2.ZIP › diagnostic_plots/Inpatient_Stratified_PACF_Affective disorders.tiff]

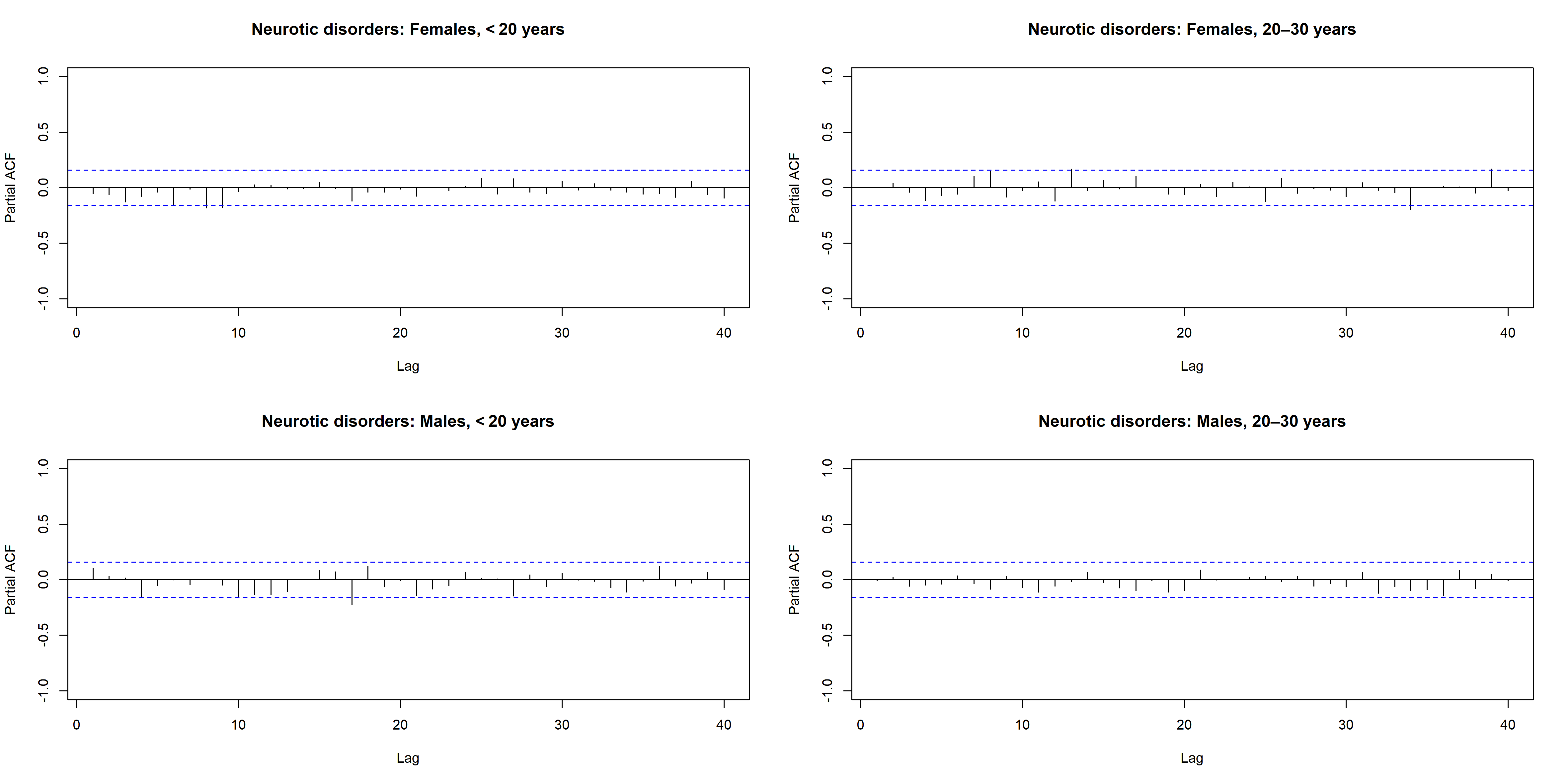

Supplement: Supplementary file 4 [file DataSheet2.ZIP › diagnostic_plots/Inpatient_Stratified_PACF_Neurotic disorders.tiff]

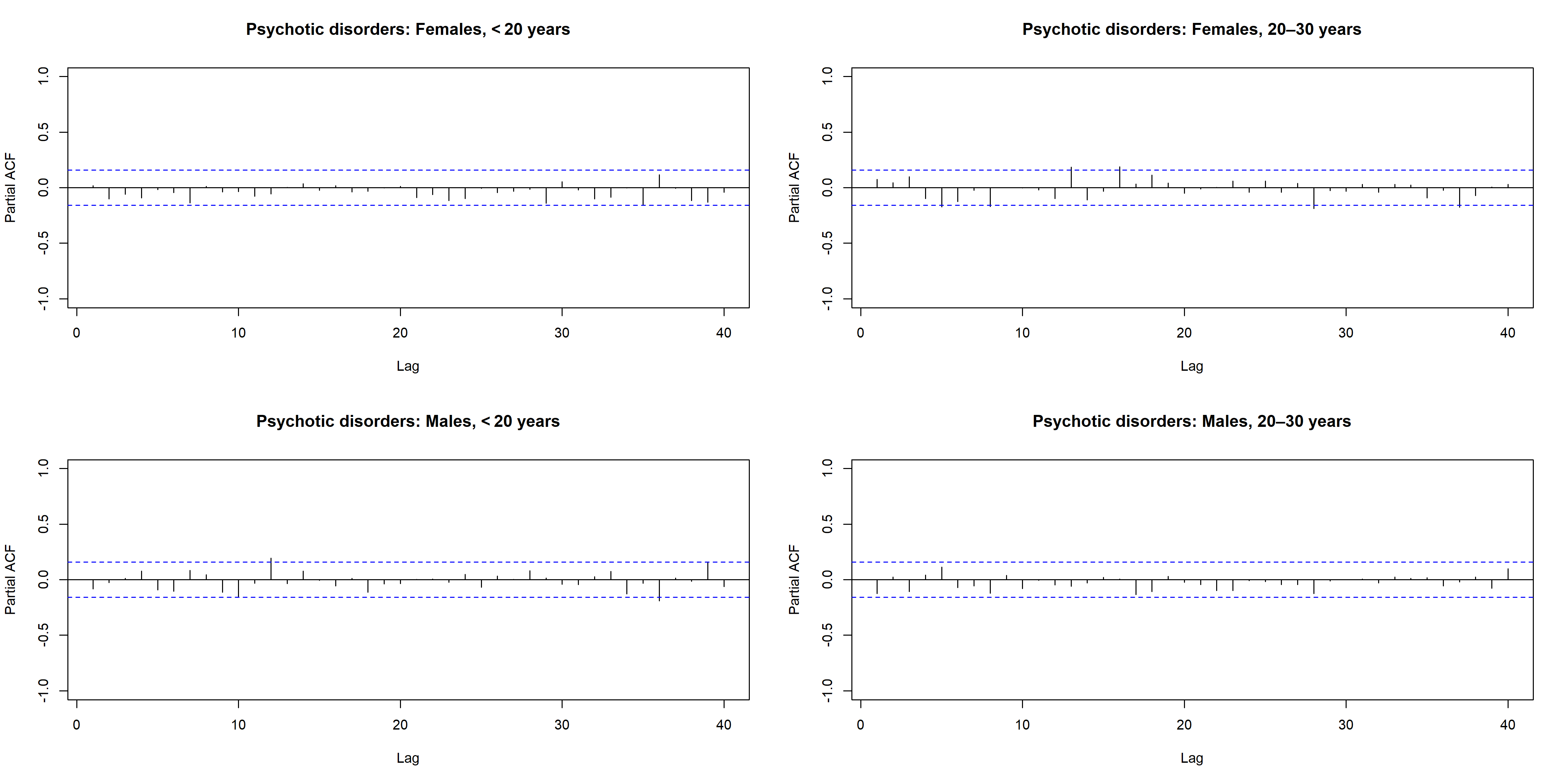

Supplement: Supplementary file 4 [file DataSheet2.ZIP › diagnostic_plots/Inpatient_Stratified_PACF_Psychotic disorders.tiff]

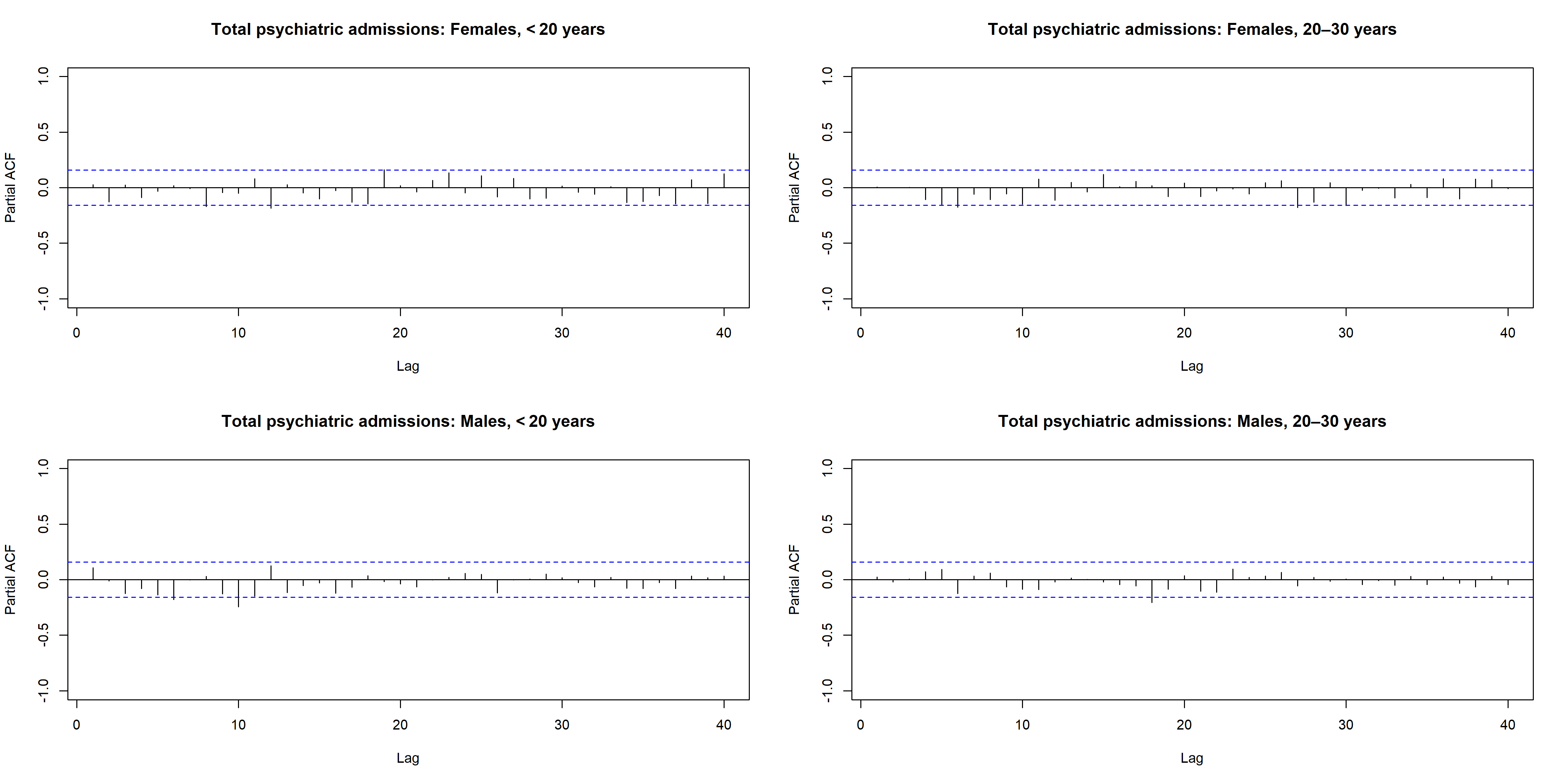

Supplement: Supplementary file 4 [file DataSheet2.ZIP › diagnostic_plots/Inpatient_Stratified_PACF_Total psychiatric admissions.tiff]

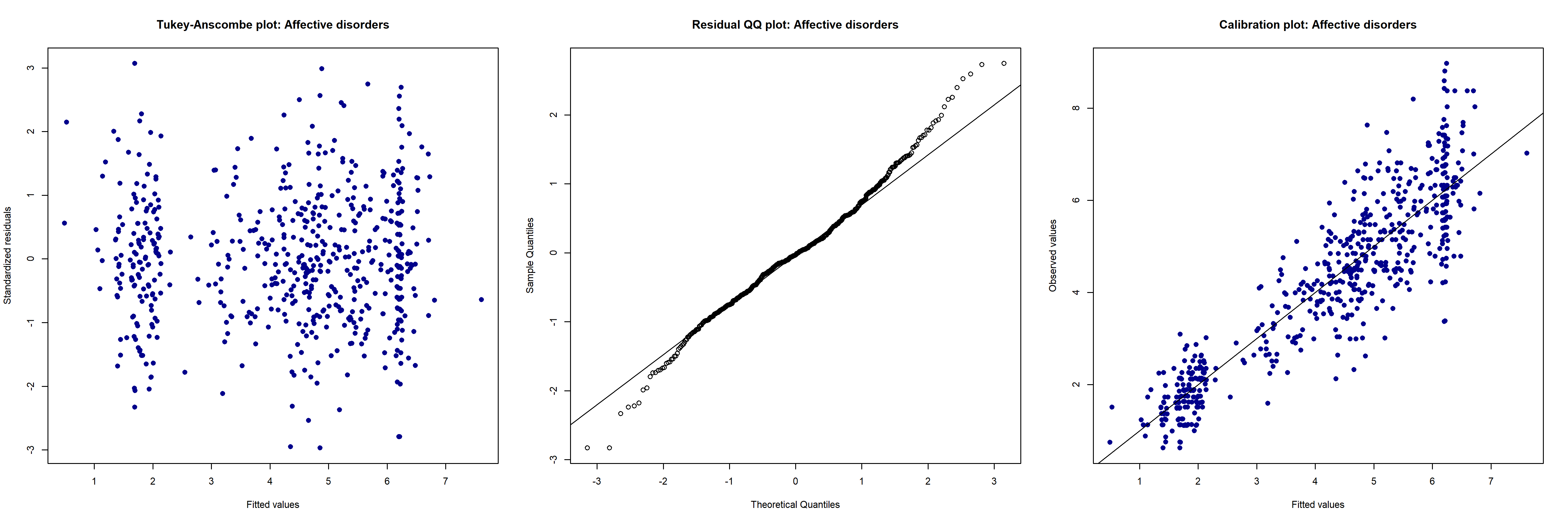

Supplement: Supplementary file 4 [file DataSheet2.ZIP › diagnostic_plots/Inpatient_Stratified_Residuals_Affective disorders.tiff]

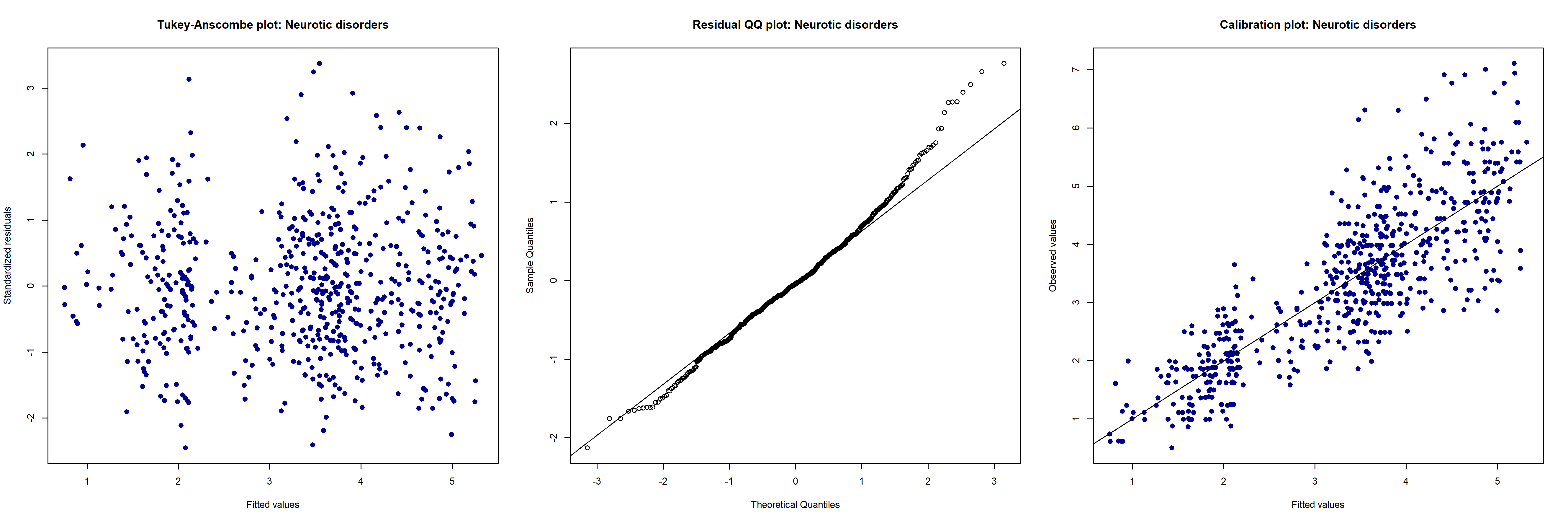

Supplement: Supplementary file 4 [file DataSheet2.ZIP › diagnostic_plots/Inpatient_Stratified_Residuals_Neurotic disorders.tiff]

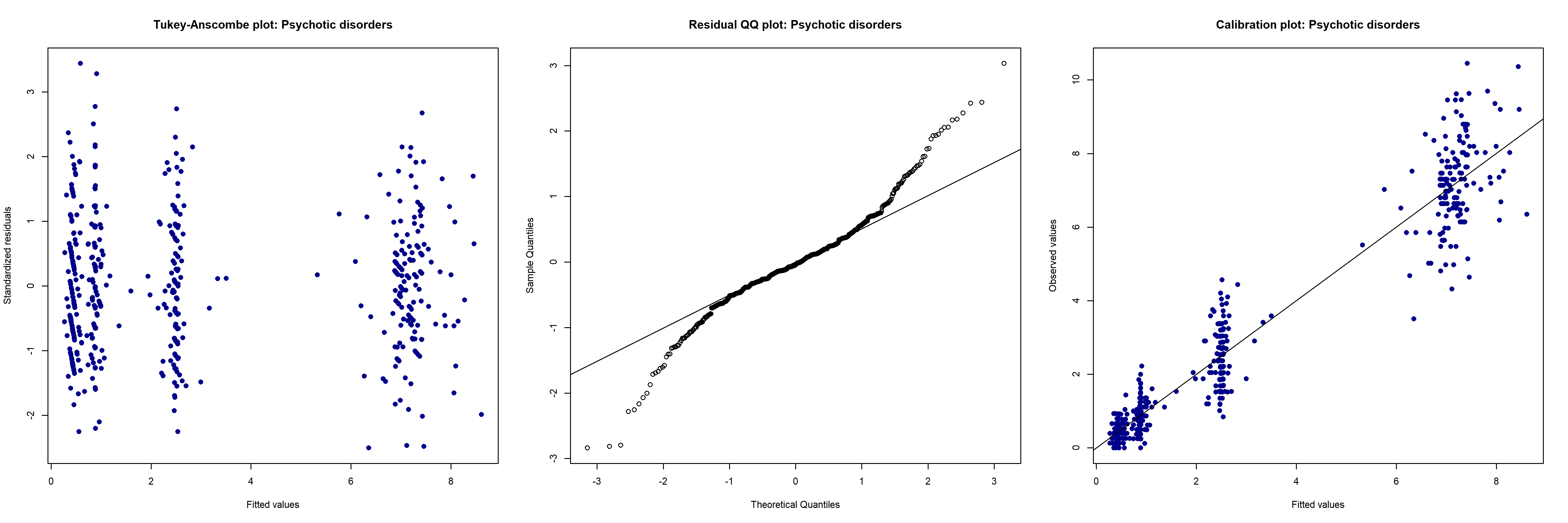

Supplement: Supplementary file 4 [file DataSheet2.ZIP › diagnostic_plots/Inpatient_Stratified_Residuals_Psychotic disorders.tiff]

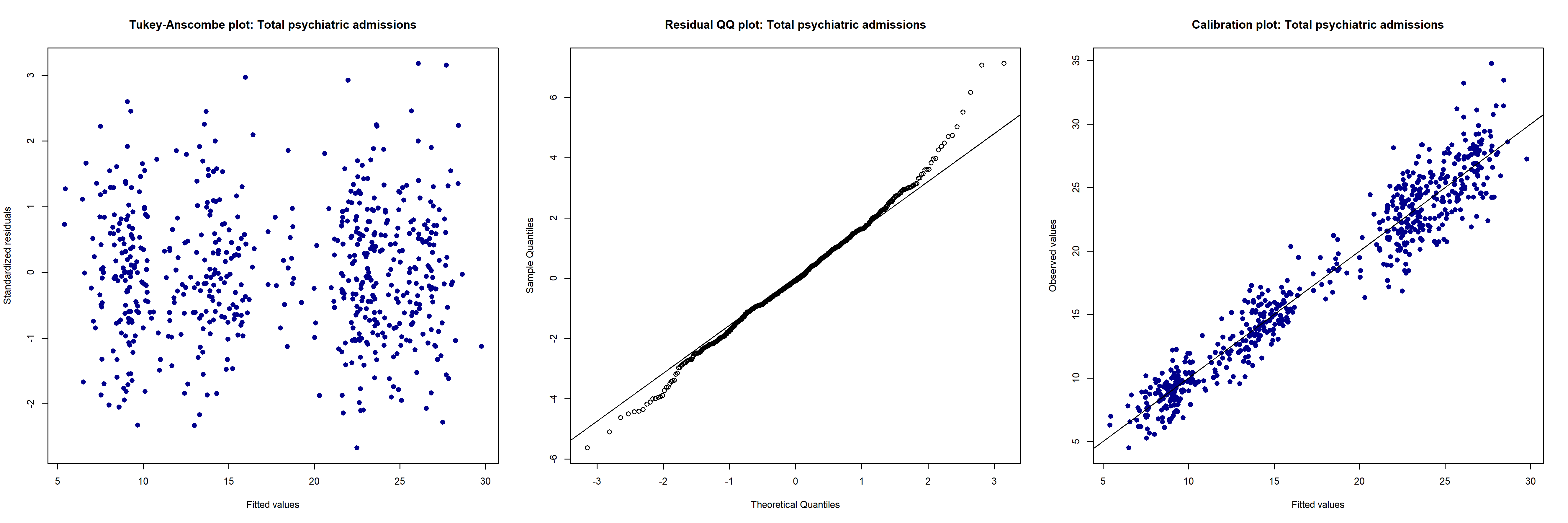

Supplement: Supplementary file 4 [file DataSheet2.ZIP › diagnostic_plots/Inpatient_Stratified_Residuals_Total psychiatric admissions.tiff]

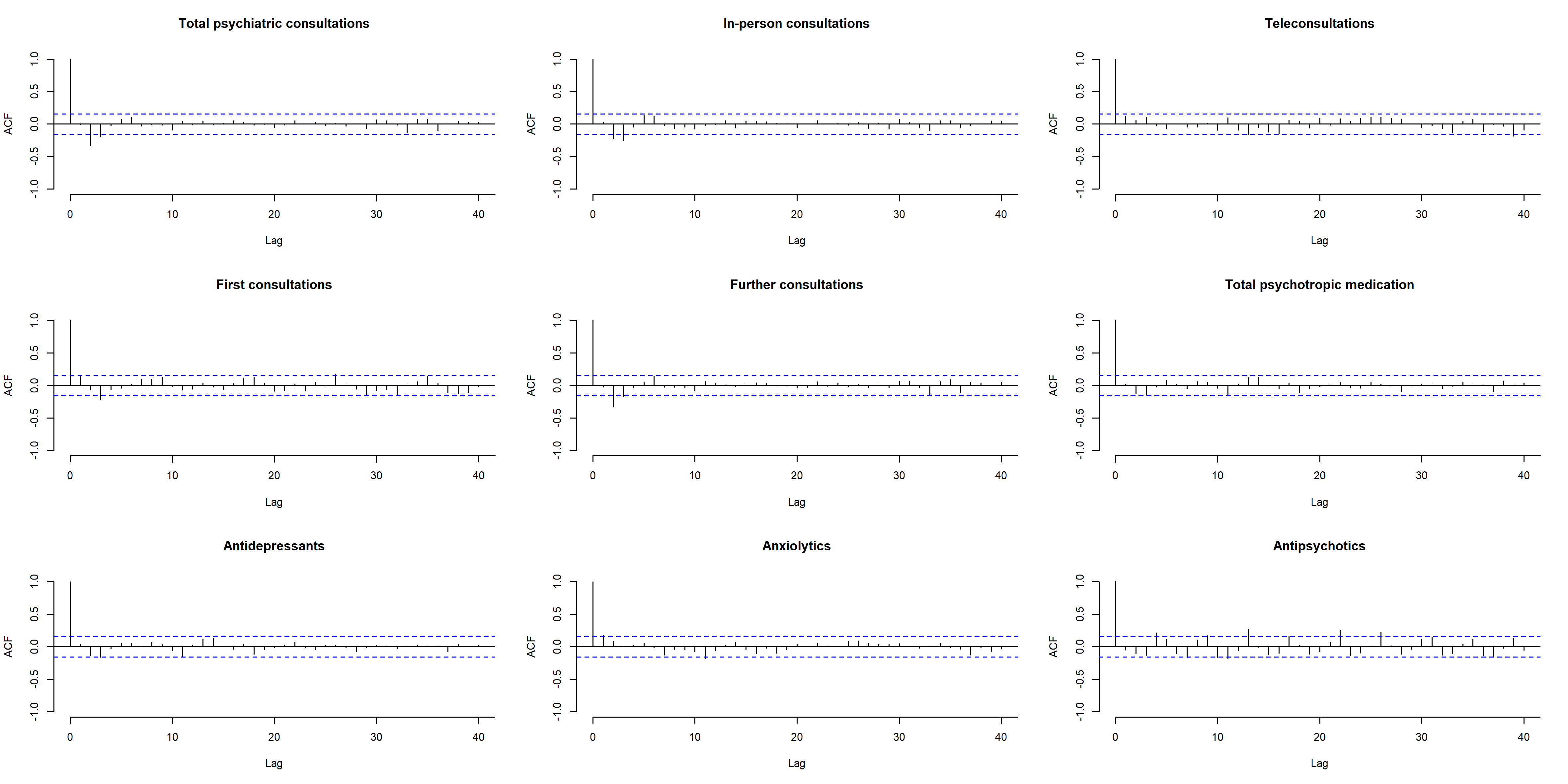

Supplement: Supplementary file 4 [file DataSheet2.ZIP › diagnostic_plots/Outpatient_Overall_ACF.tiff]

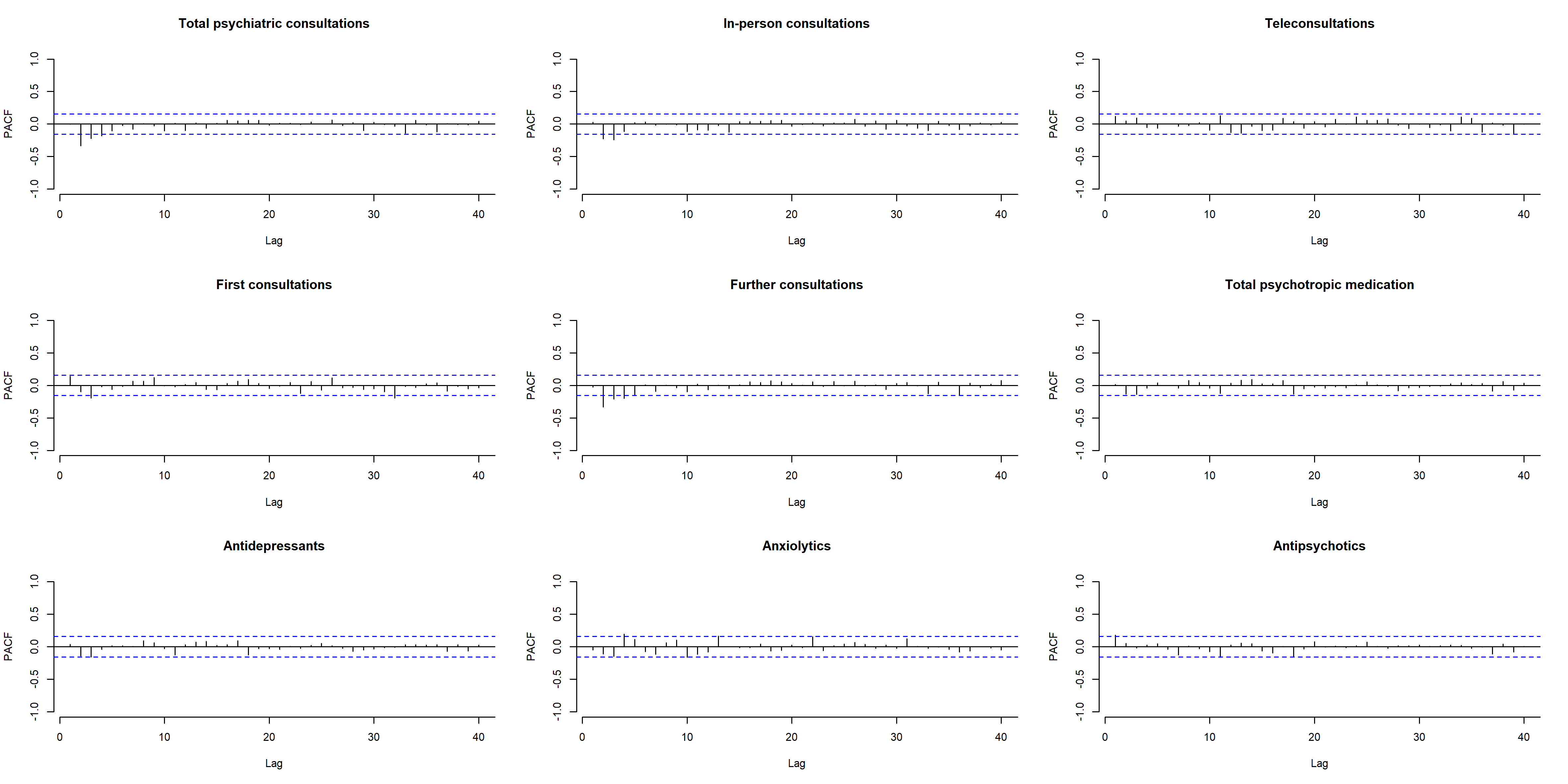

Supplement: Supplementary file 4 [file DataSheet2.ZIP › diagnostic_plots/Outpatient_Overall_PACFs.tiff]

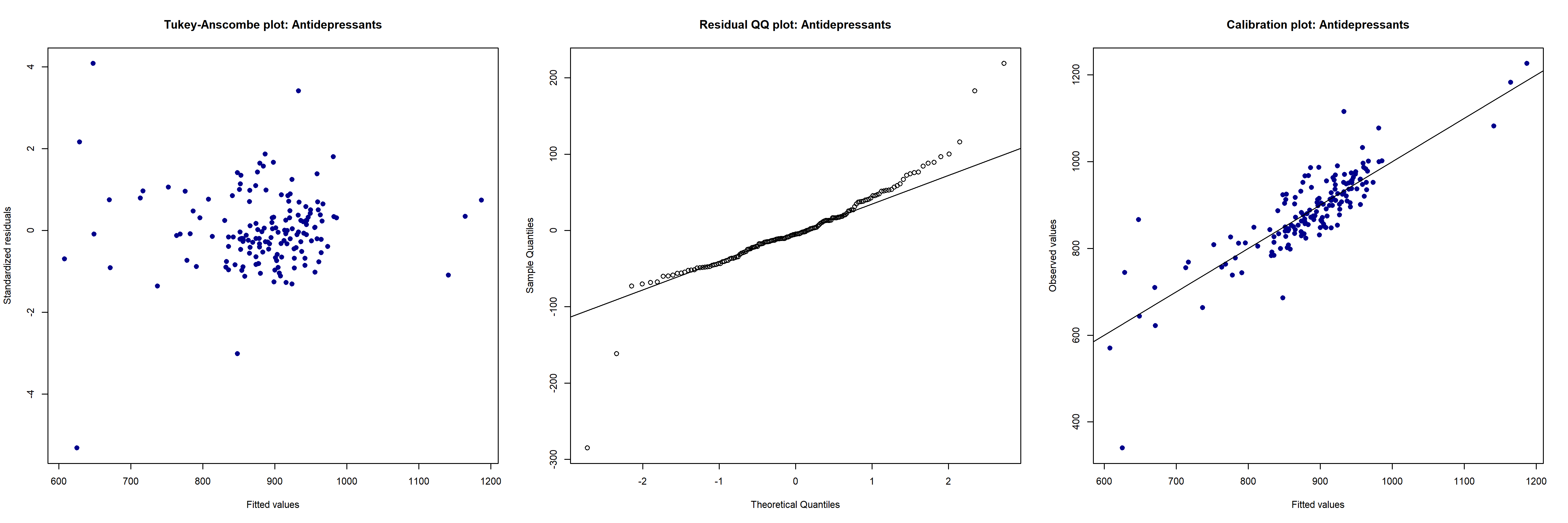

Supplement: Supplementary file 4 [file DataSheet2.ZIP › diagnostic_plots/Outpatient_Overall_Residuals_Antidepressants.tiff]

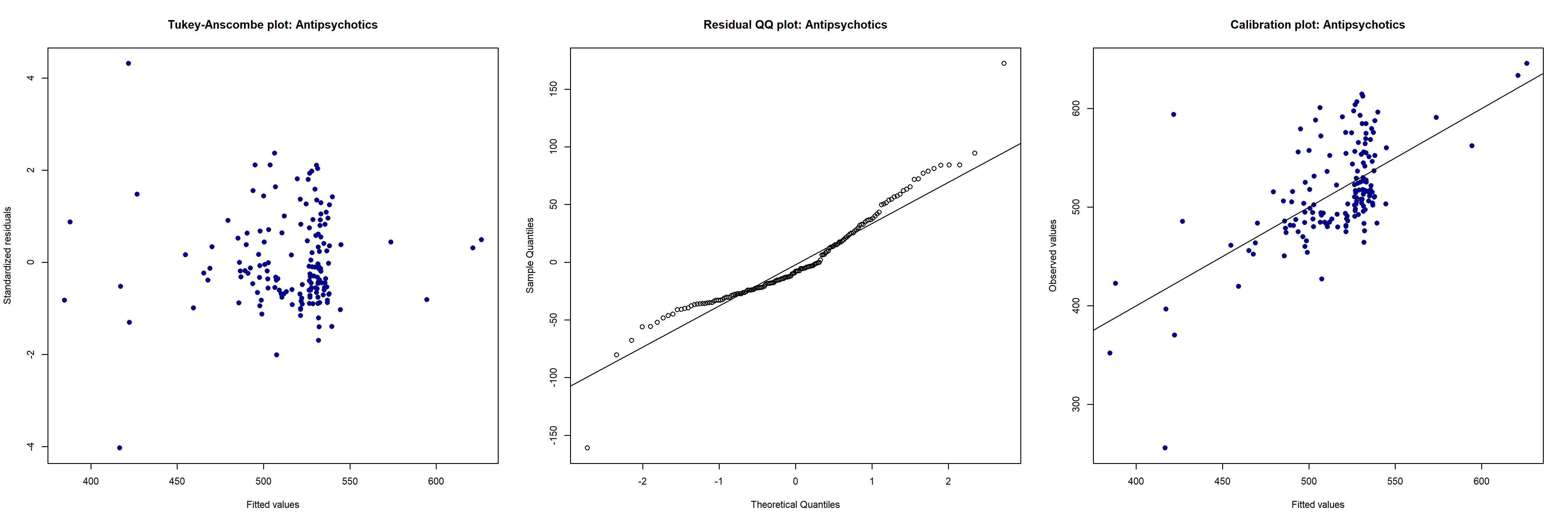

Supplement: Supplementary file 4 [file DataSheet2.ZIP › diagnostic_plots/Outpatient_Overall_Residuals_Antipsychotics.tiff]

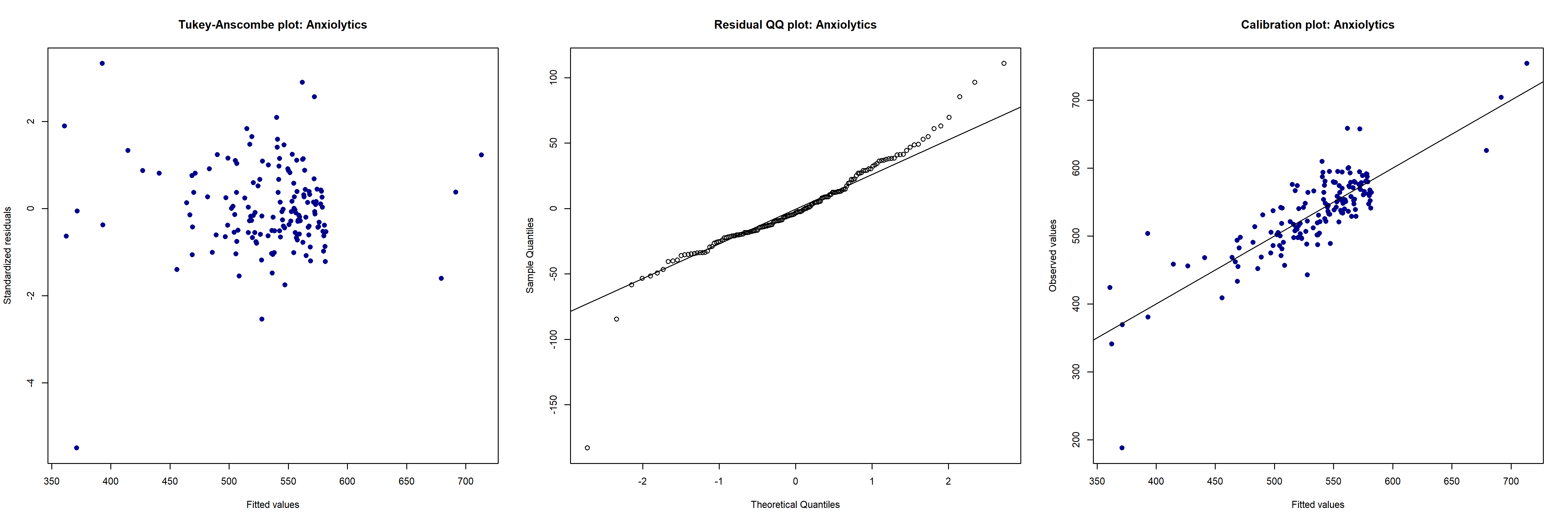

Supplement: Supplementary file 4 [file DataSheet2.ZIP › diagnostic_plots/Outpatient_Overall_Residuals_Anxiolytics.tiff]

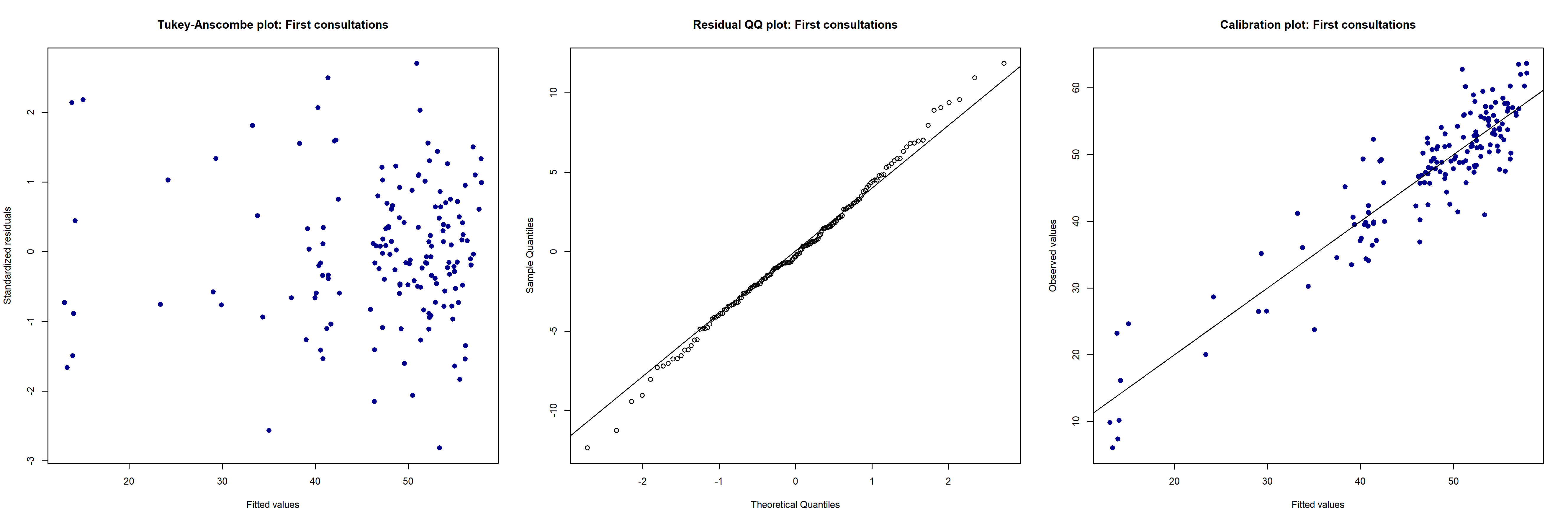

Supplement: Supplementary file 4 [file DataSheet2.ZIP › diagnostic_plots/Outpatient_Overall_Residuals_First consultations.tiff]

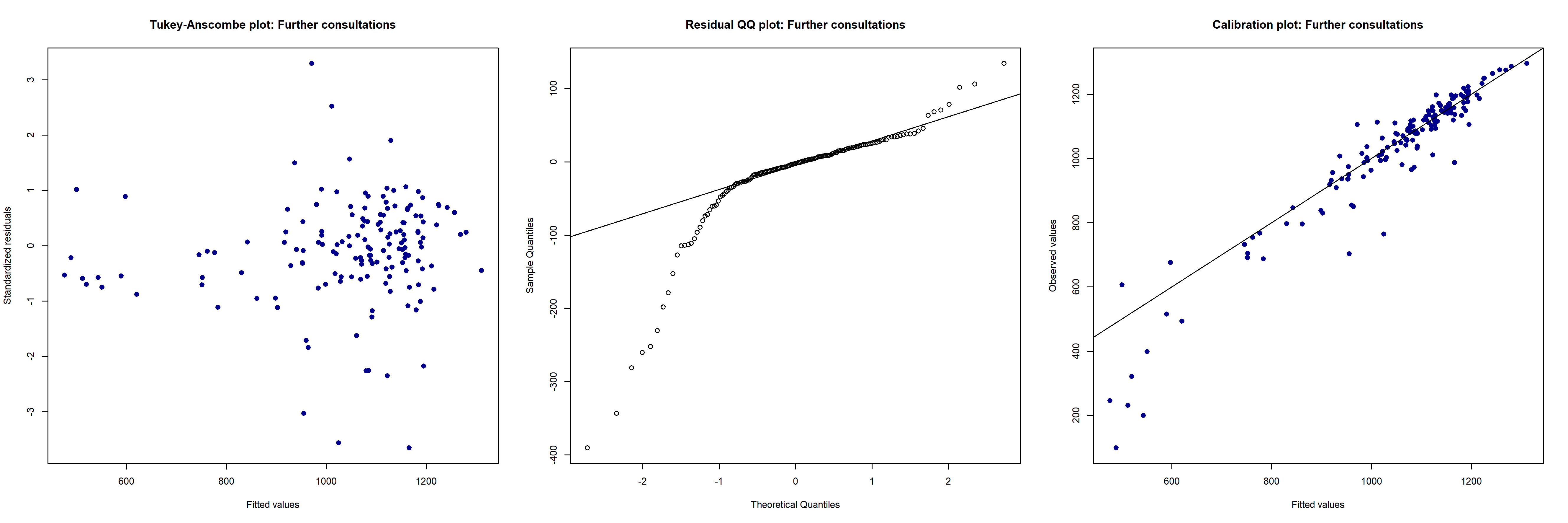

Supplement: Supplementary file 4 [file DataSheet2.ZIP › diagnostic_plots/Outpatient_Overall_Residuals_Further consultations.tiff]

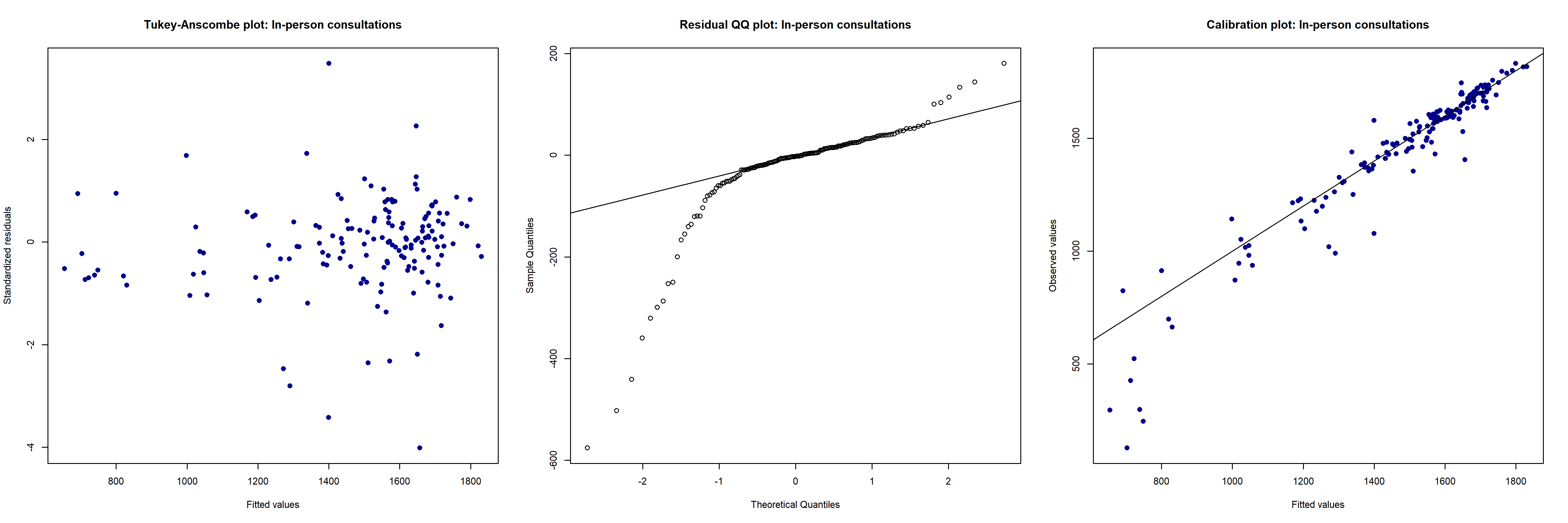

Supplement: Supplementary file 4 [file DataSheet2.ZIP › diagnostic_plots/Outpatient_Overall_Residuals_In-person consultations.tiff]

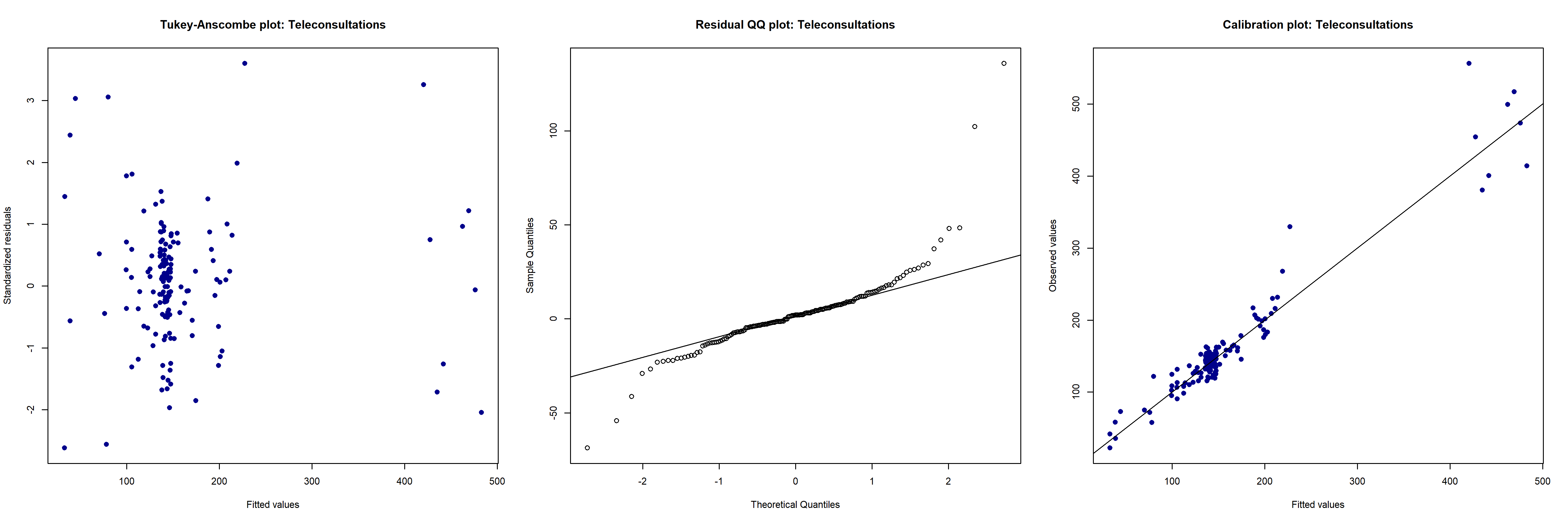

Supplement: Supplementary file 4 [file DataSheet2.ZIP › diagnostic_plots/Outpatient_Overall_Residuals_Teleconsultations.tiff]

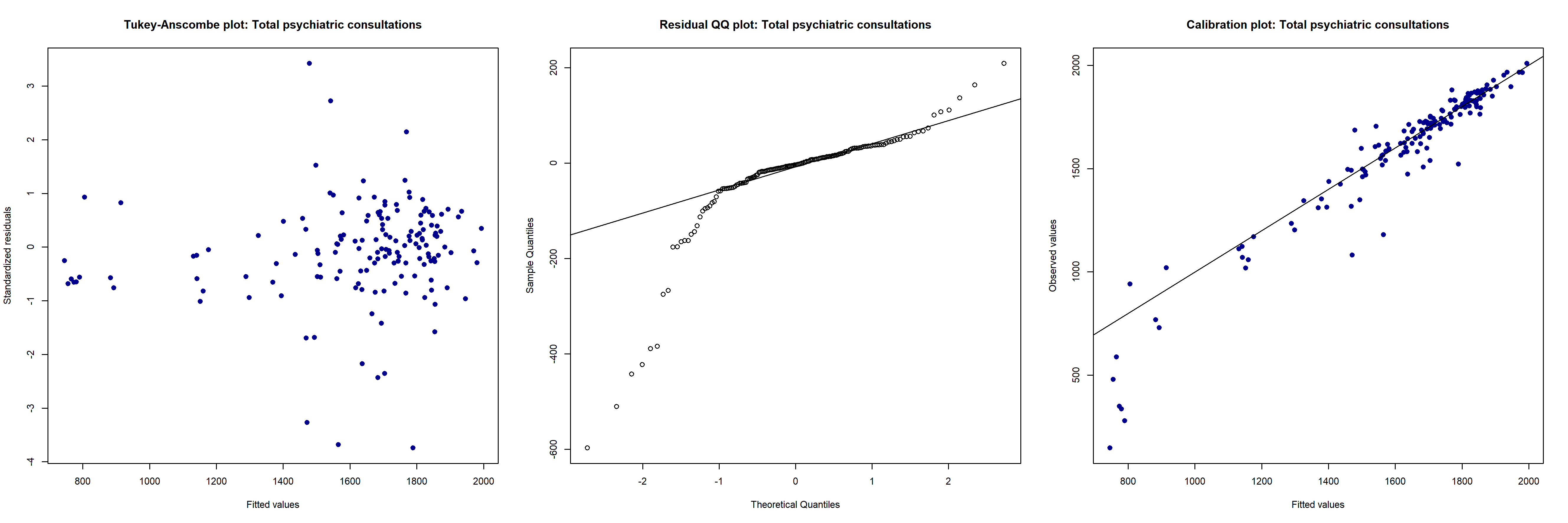

Supplement: Supplementary file 4 [file DataSheet2.ZIP › diagnostic_plots/Outpatient_Overall_Residuals_Total psychiatric consultations.tiff]

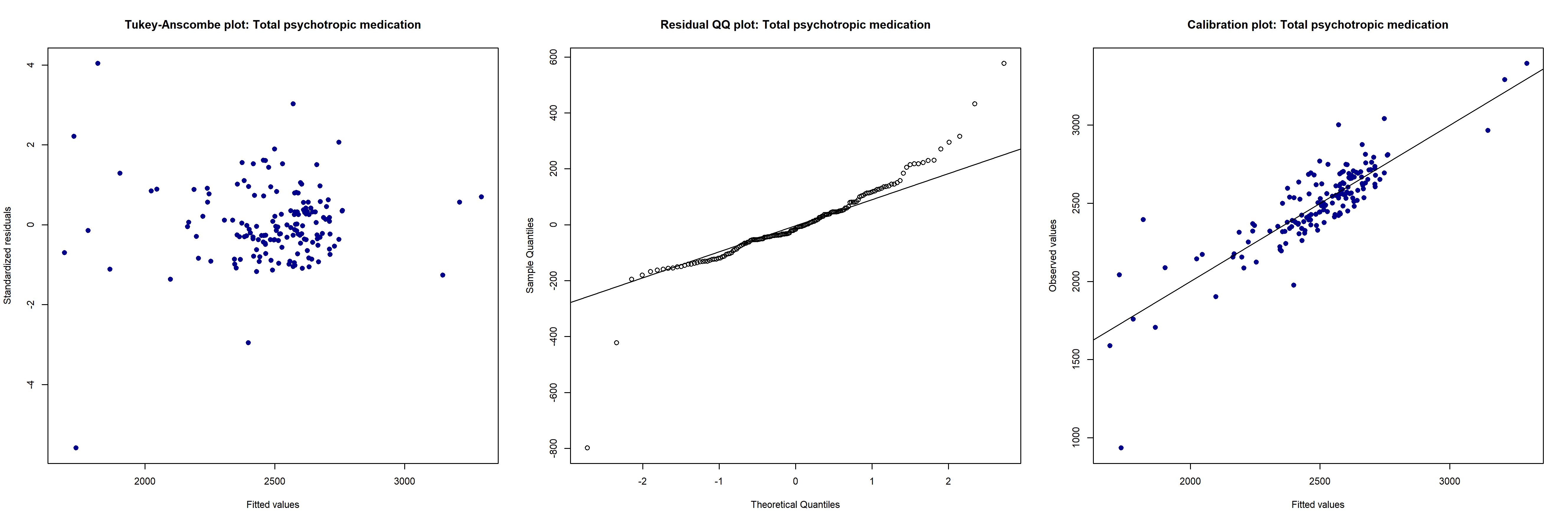

Supplement: Supplementary file 4 [file DataSheet2.ZIP › diagnostic_plots/Outpatient_Overall_Residuals_Total psychotropic medication.tiff]

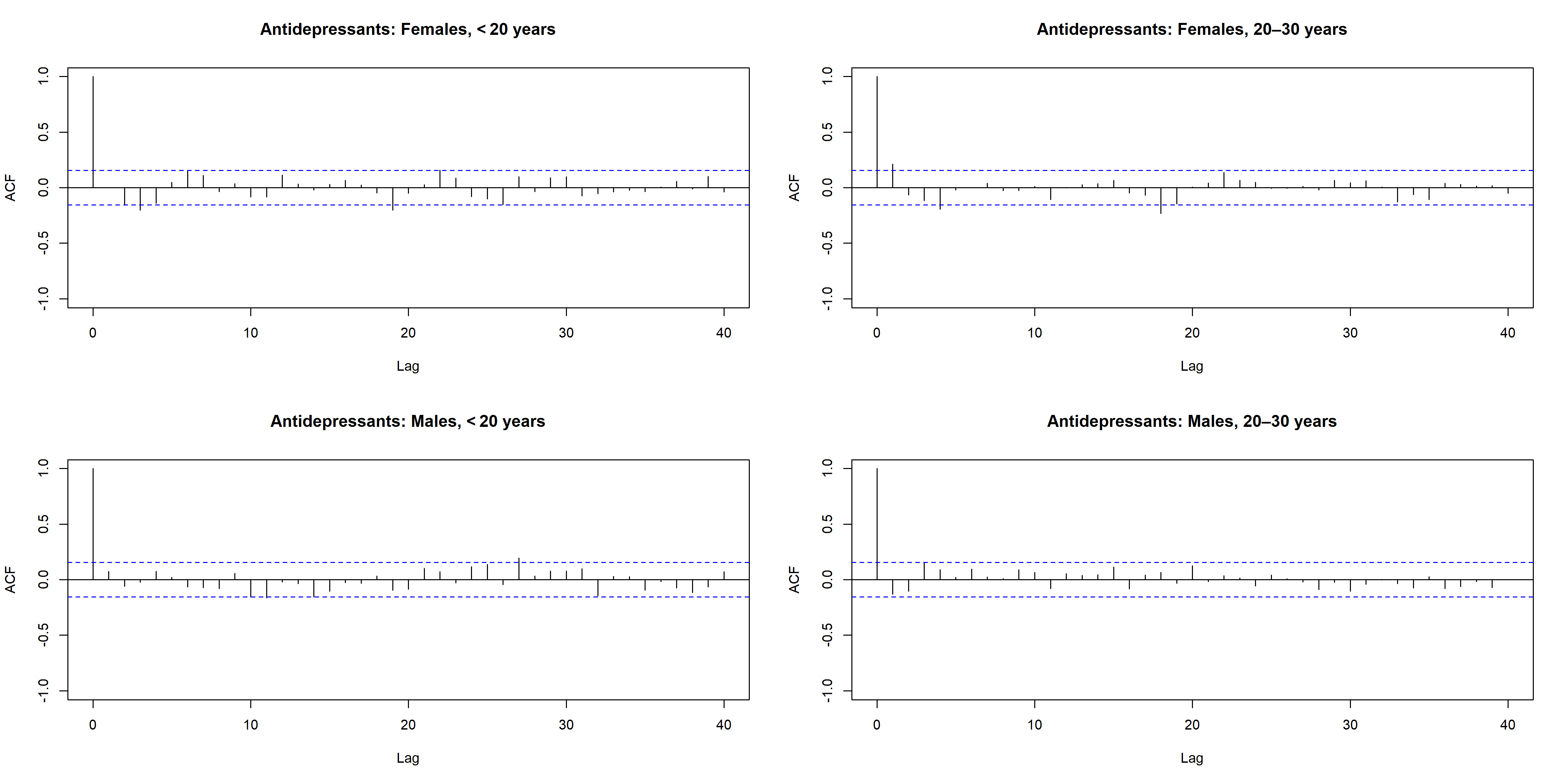

Supplement: Supplementary file 4 [file DataSheet2.ZIP › diagnostic_plots/Outpatient_Stratified_ACF_Antidepressants.tiff]

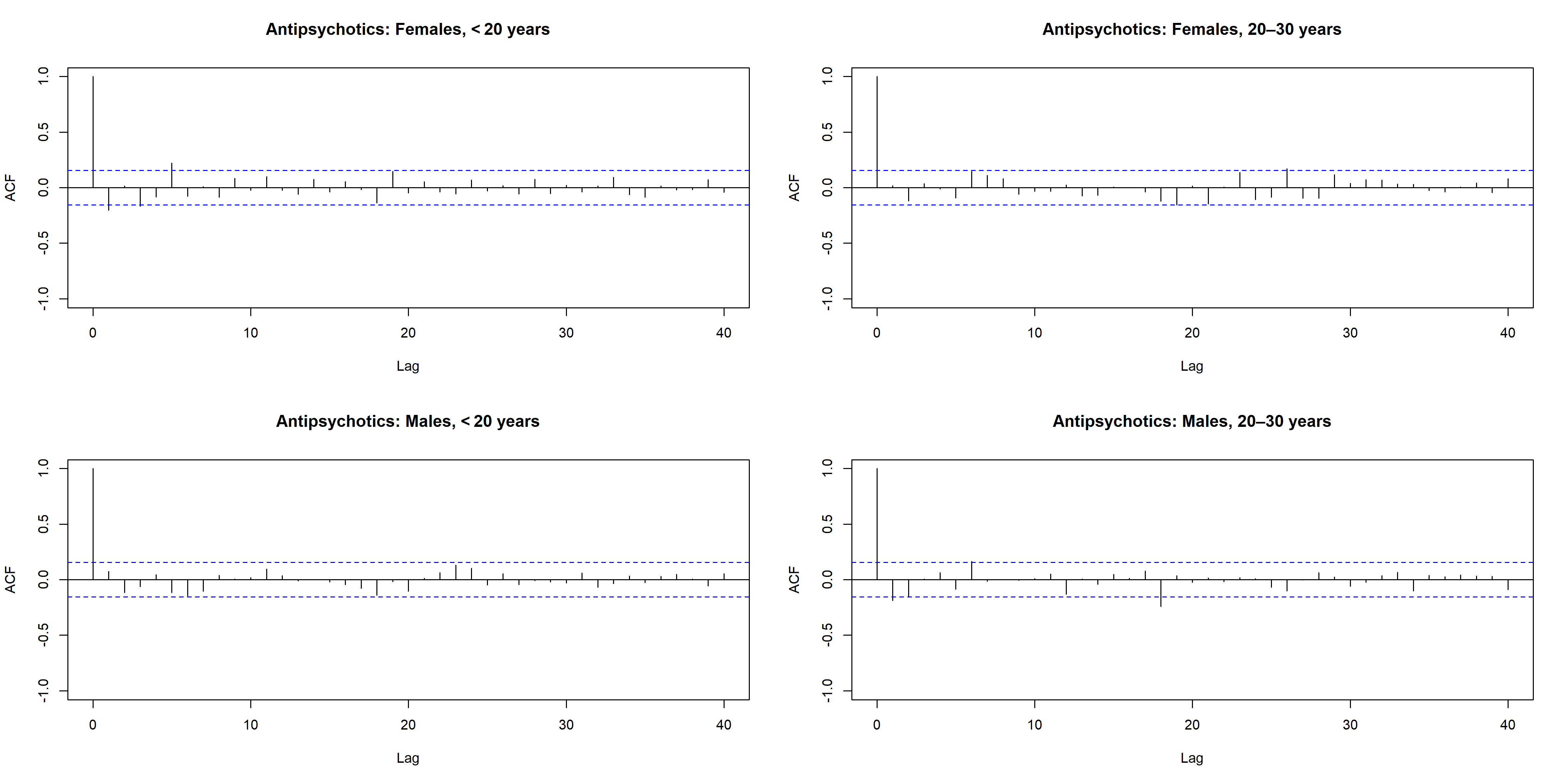

Supplement: Supplementary file 4 [file DataSheet2.ZIP › diagnostic_plots/Outpatient_Stratified_ACF_Antipsychotics.tiff]

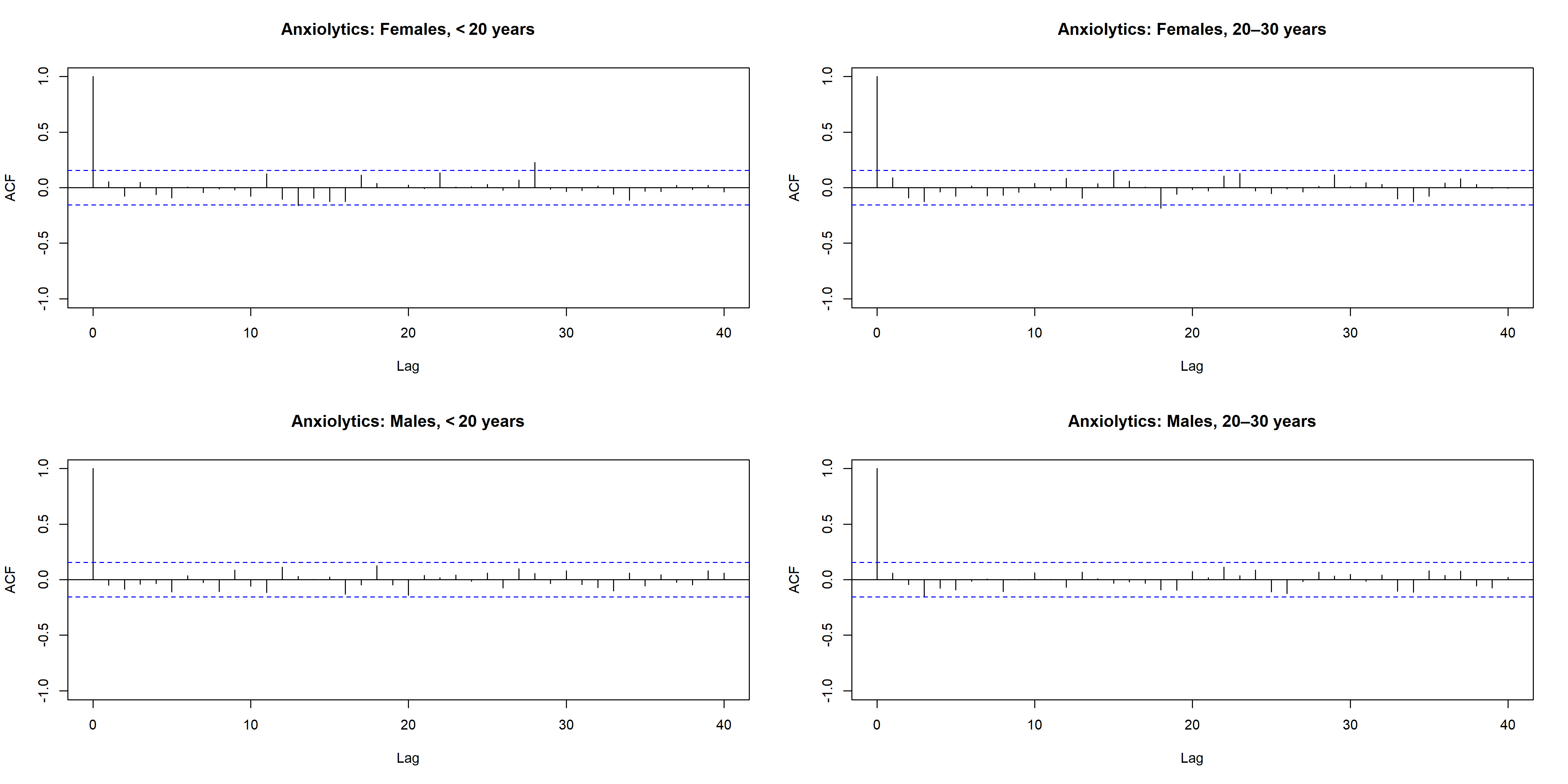

Supplement: Supplementary file 4 [file DataSheet2.ZIP › diagnostic_plots/Outpatient_Stratified_ACF_Anxiolytics.tiff]

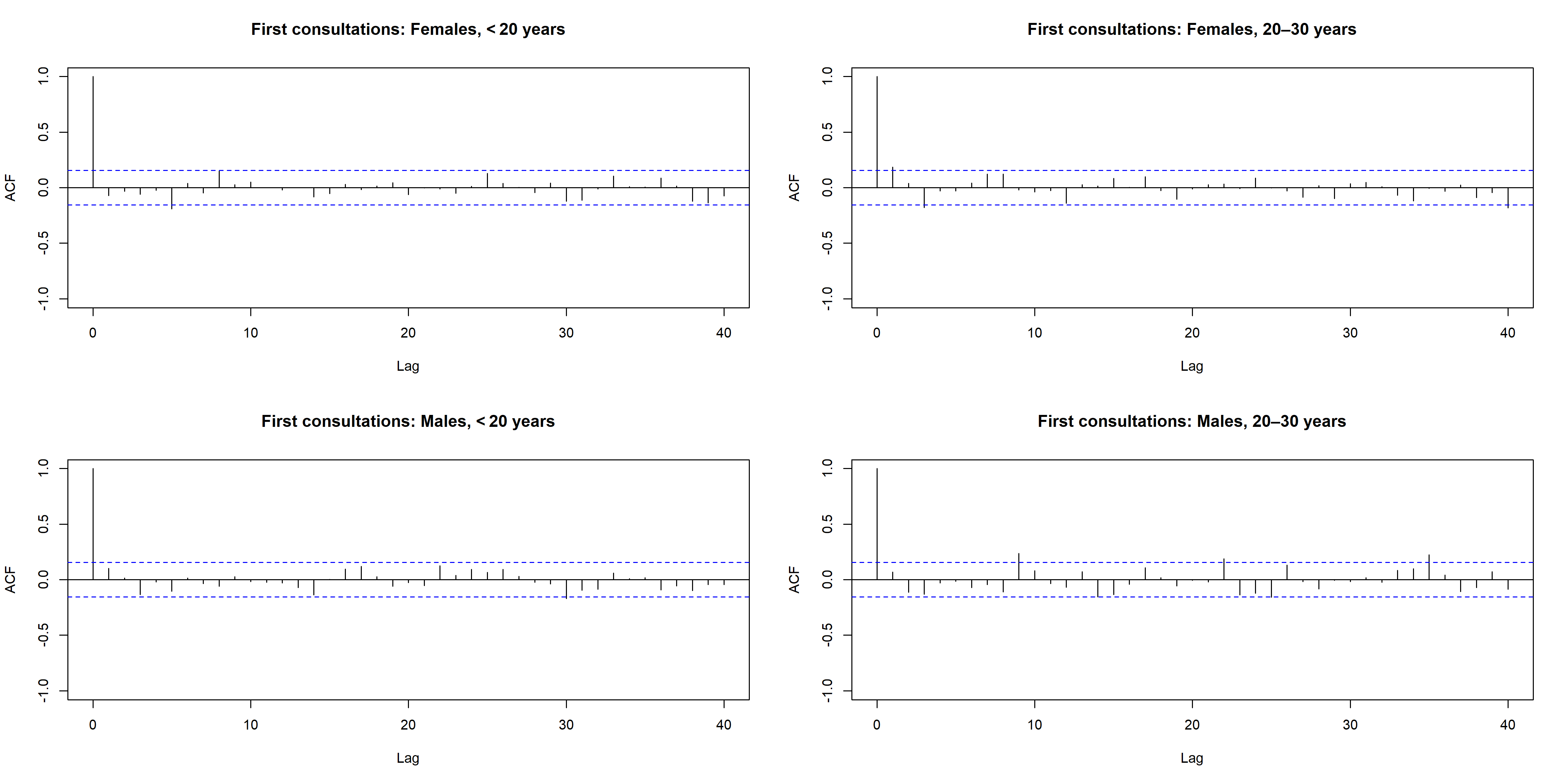

Supplement: Supplementary file 4 [file DataSheet2.ZIP › diagnostic_plots/Outpatient_Stratified_ACF_First consultations.tiff]

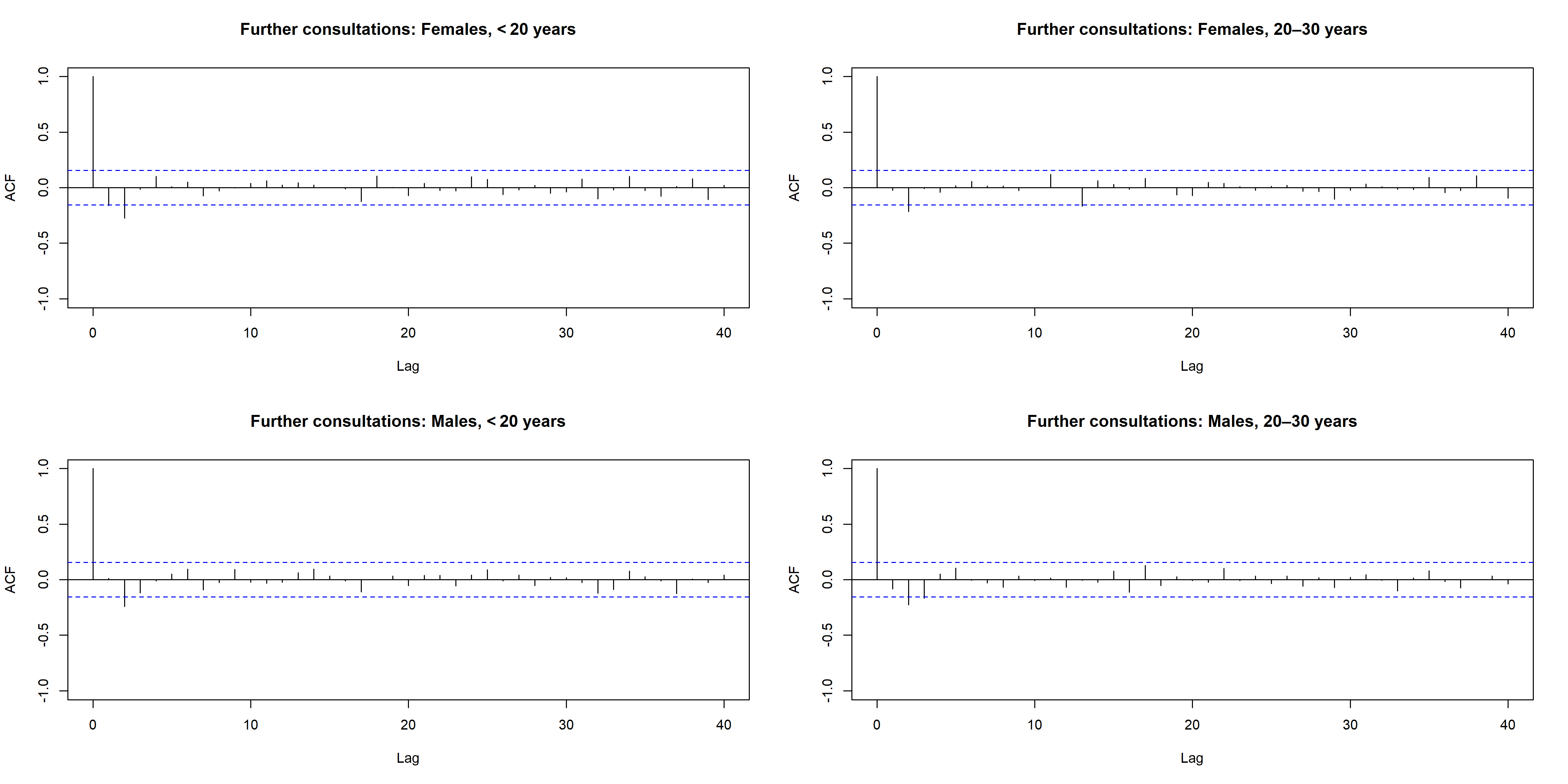

Supplement: Supplementary file 4 [file DataSheet2.ZIP › diagnostic_plots/Outpatient_Stratified_ACF_Further consultations.tiff]

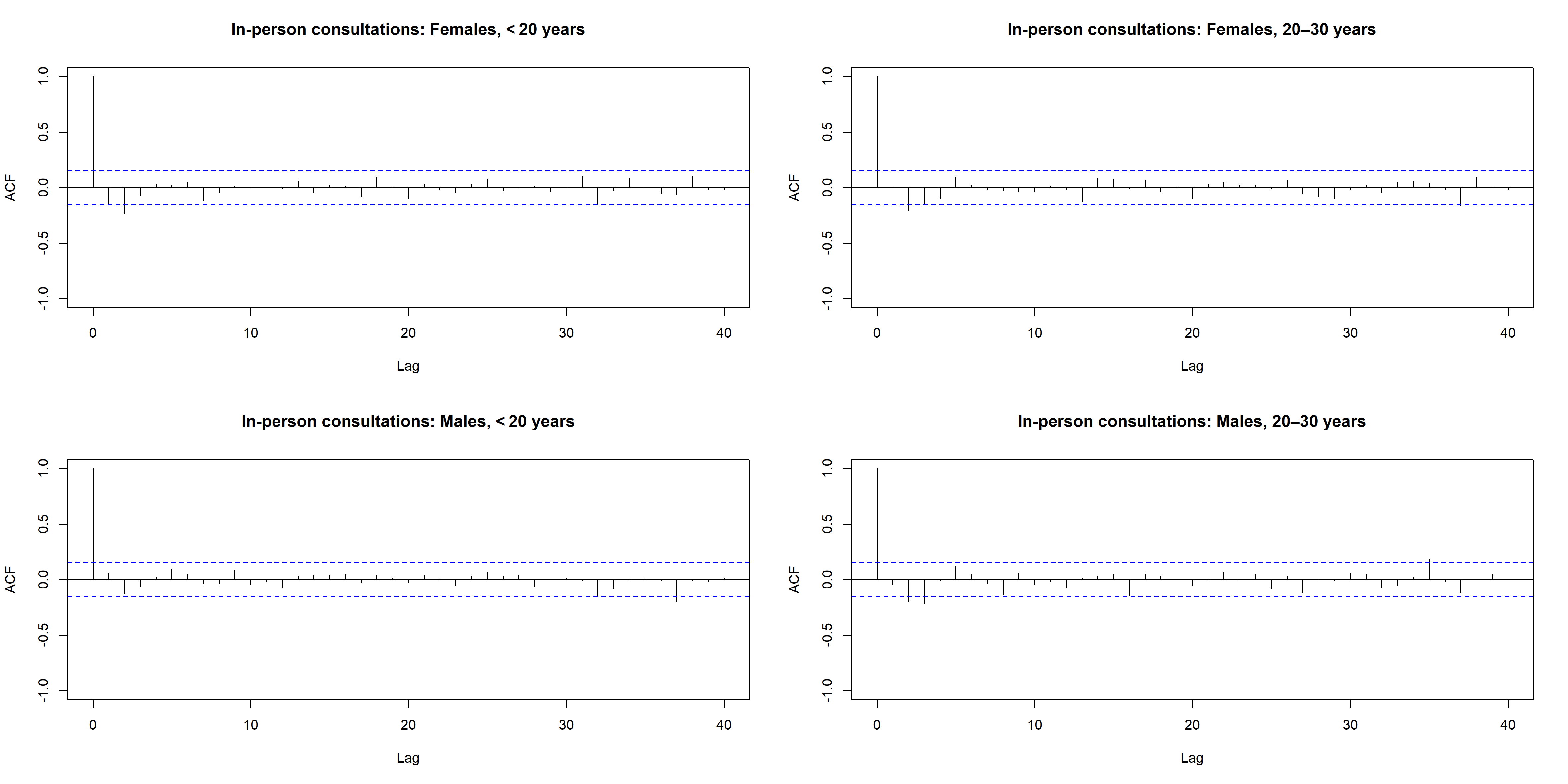

Supplement: Supplementary file 4 [file DataSheet2.ZIP › diagnostic_plots/Outpatient_Stratified_ACF_In-person consultations.tiff]

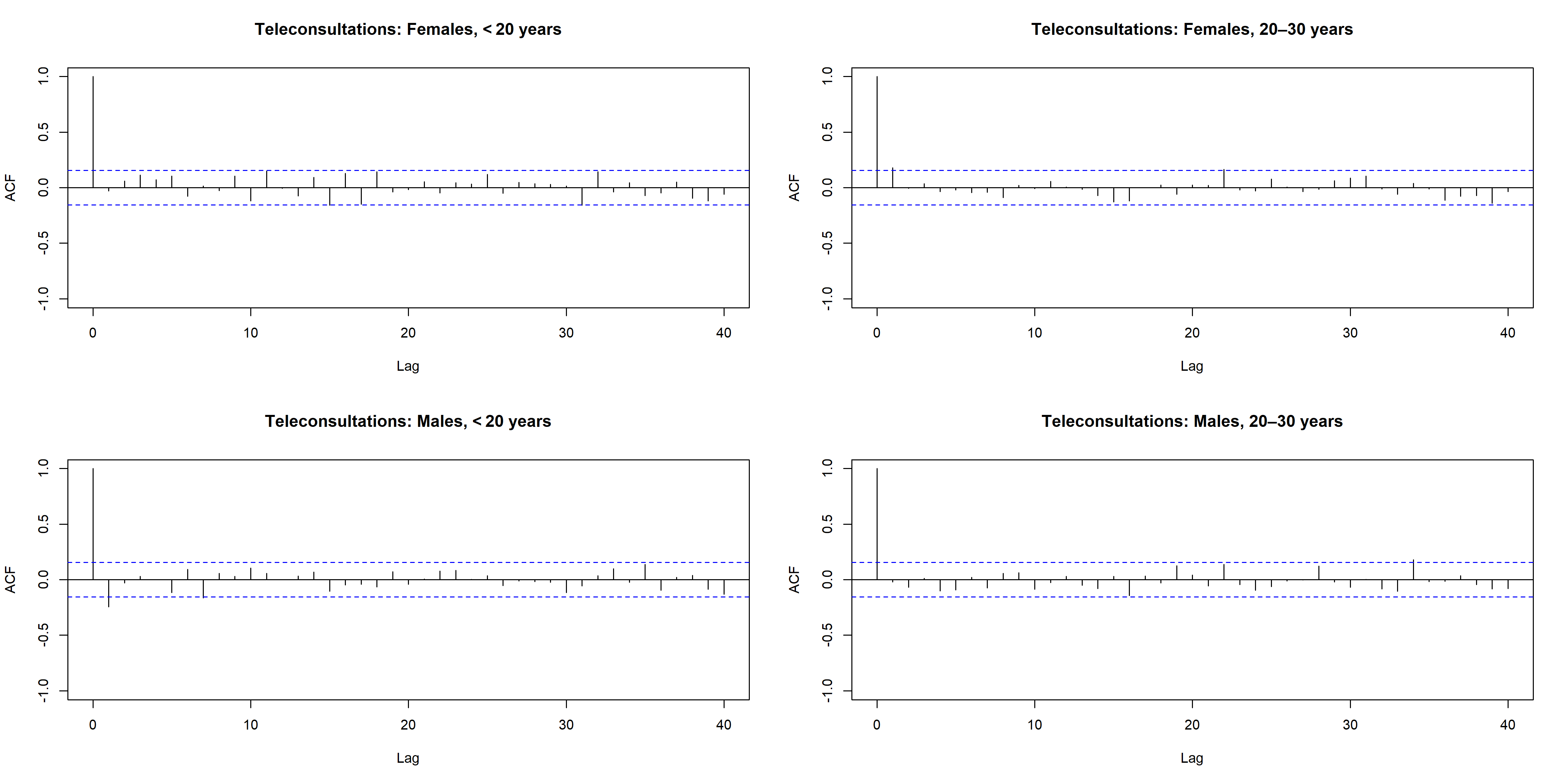

Supplement: Supplementary file 4 [file DataSheet2.ZIP › diagnostic_plots/Outpatient_Stratified_ACF_Teleconsultations.tiff]

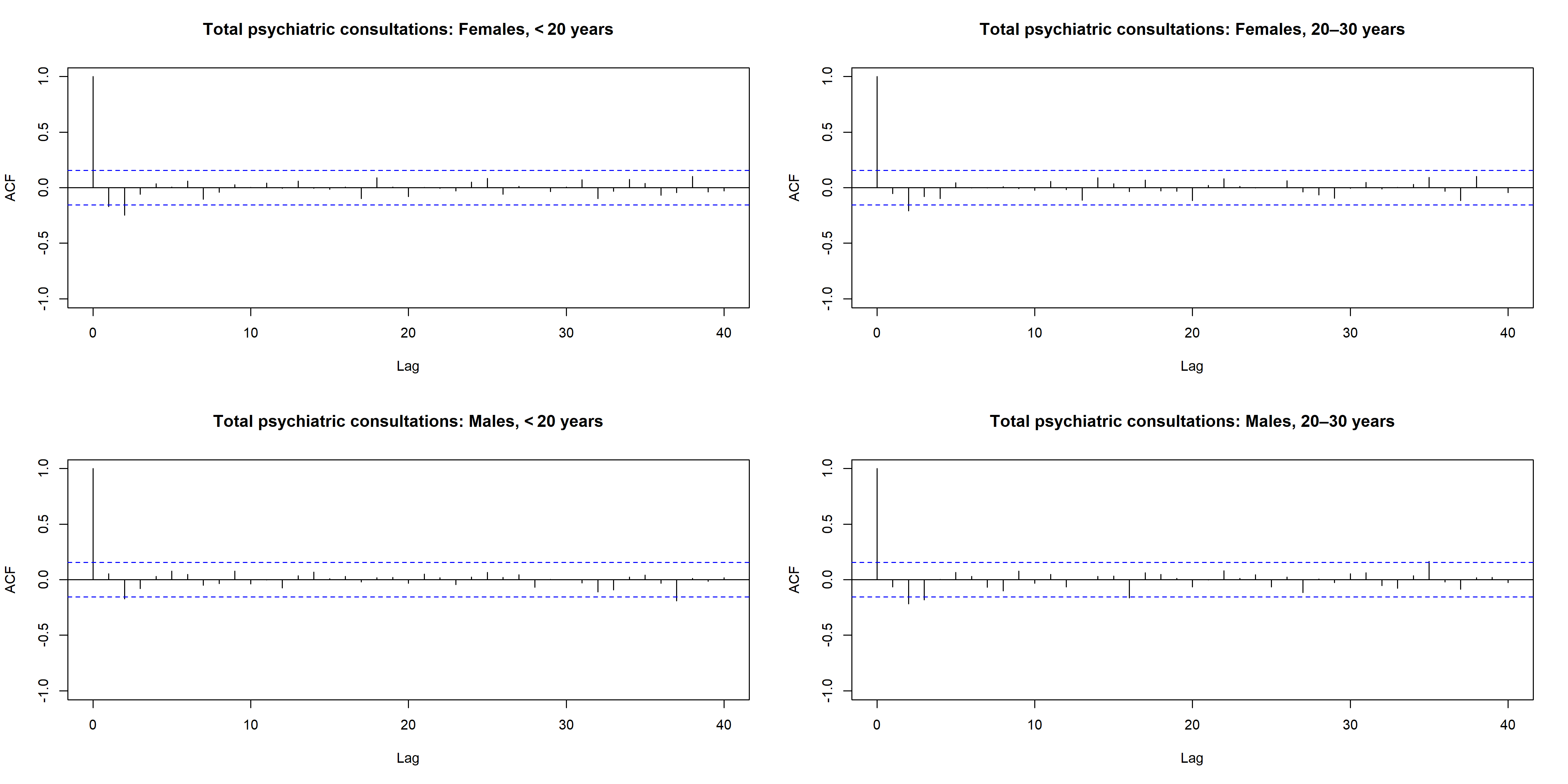

Supplement: Supplementary file 4 [file DataSheet2.ZIP › diagnostic_plots/Outpatient_Stratified_ACF_Total psychiatric consultations.tiff]

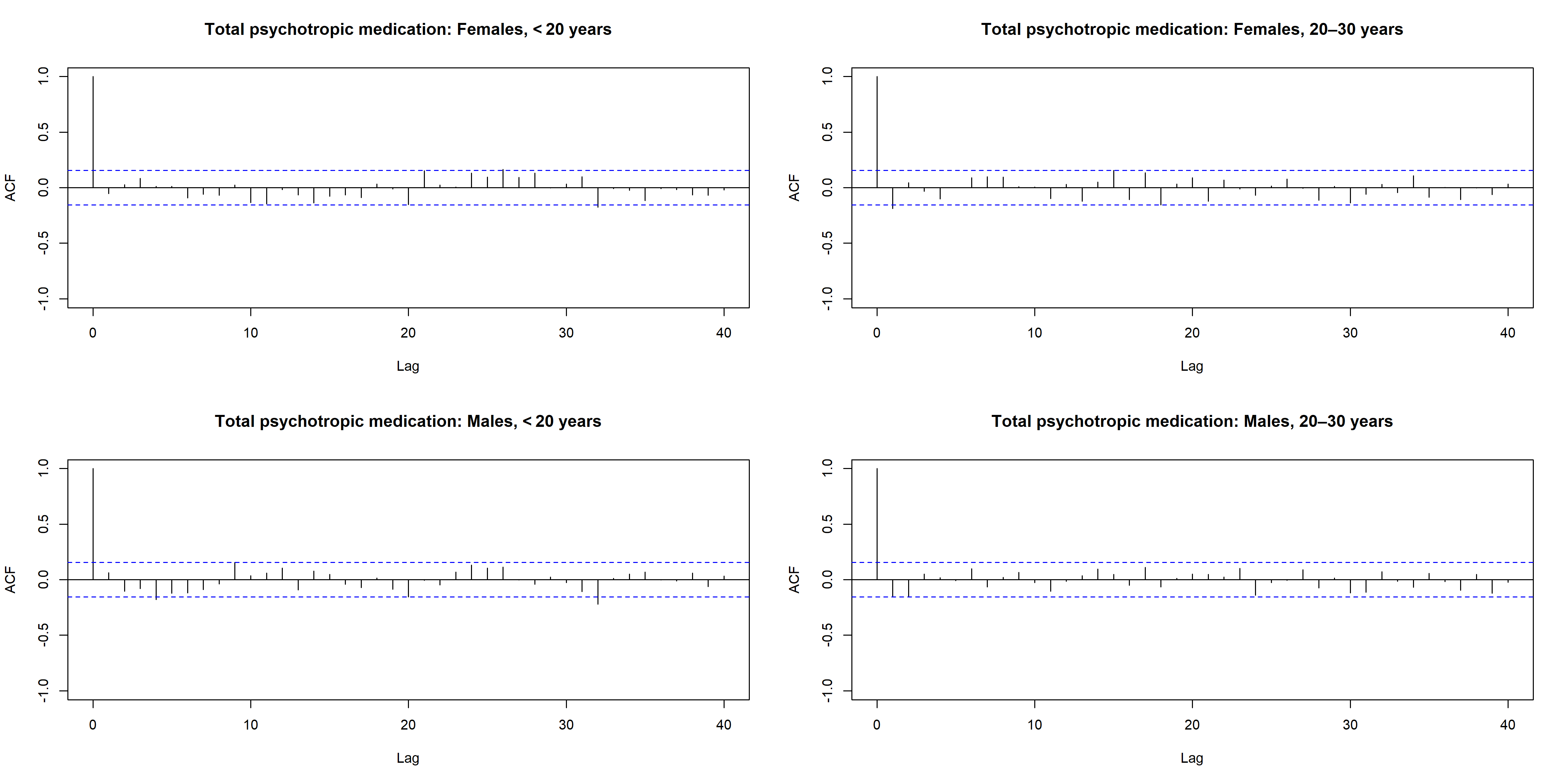

Supplement: Supplementary file 4 [file DataSheet2.ZIP › diagnostic_plots/Outpatient_Stratified_ACF_Total psychotropic medication.tiff]

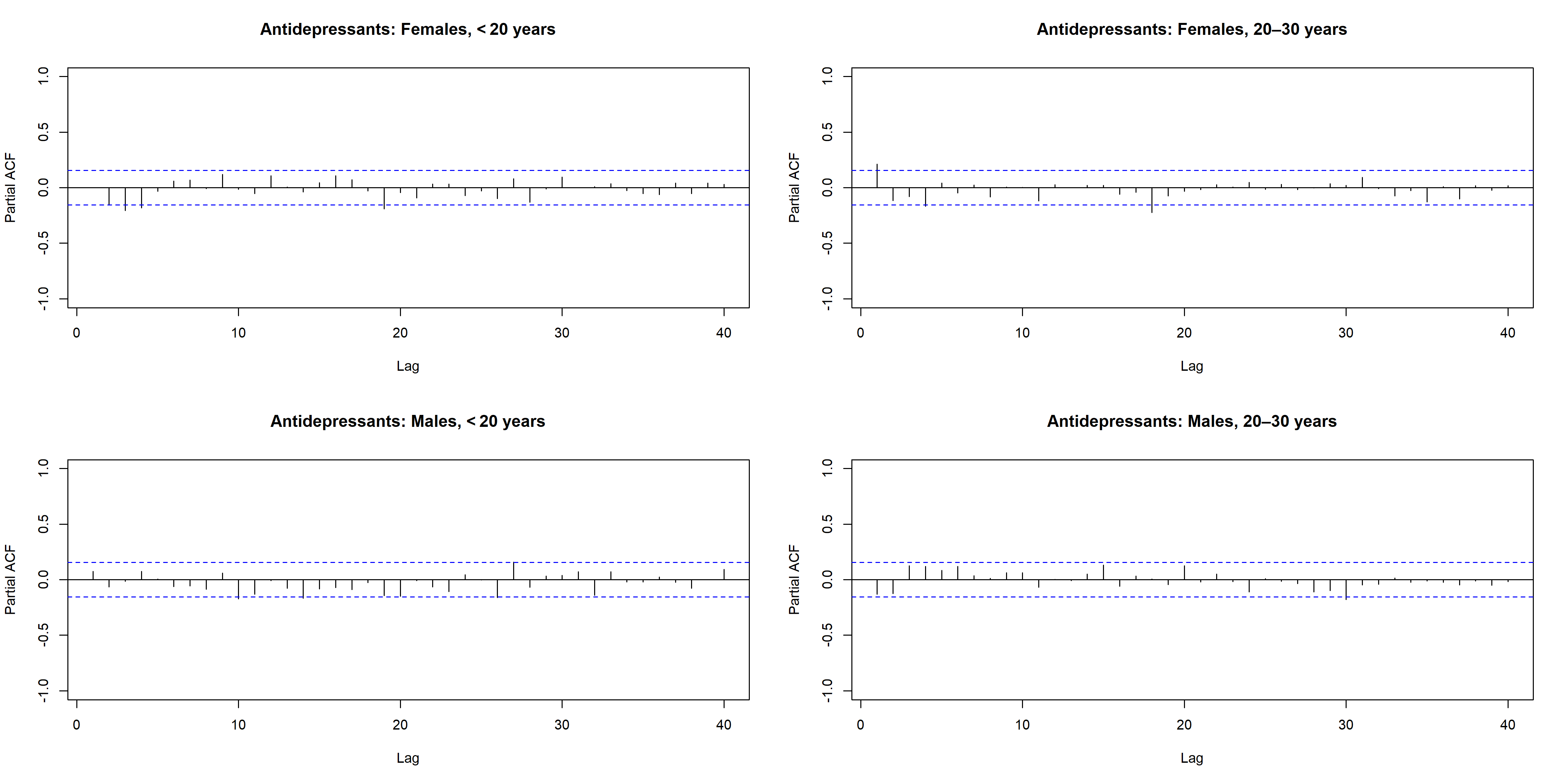

Supplement: Supplementary file 4 [file DataSheet2.ZIP › diagnostic_plots/Outpatient_Stratified_PACF_Antidepressants.tiff]

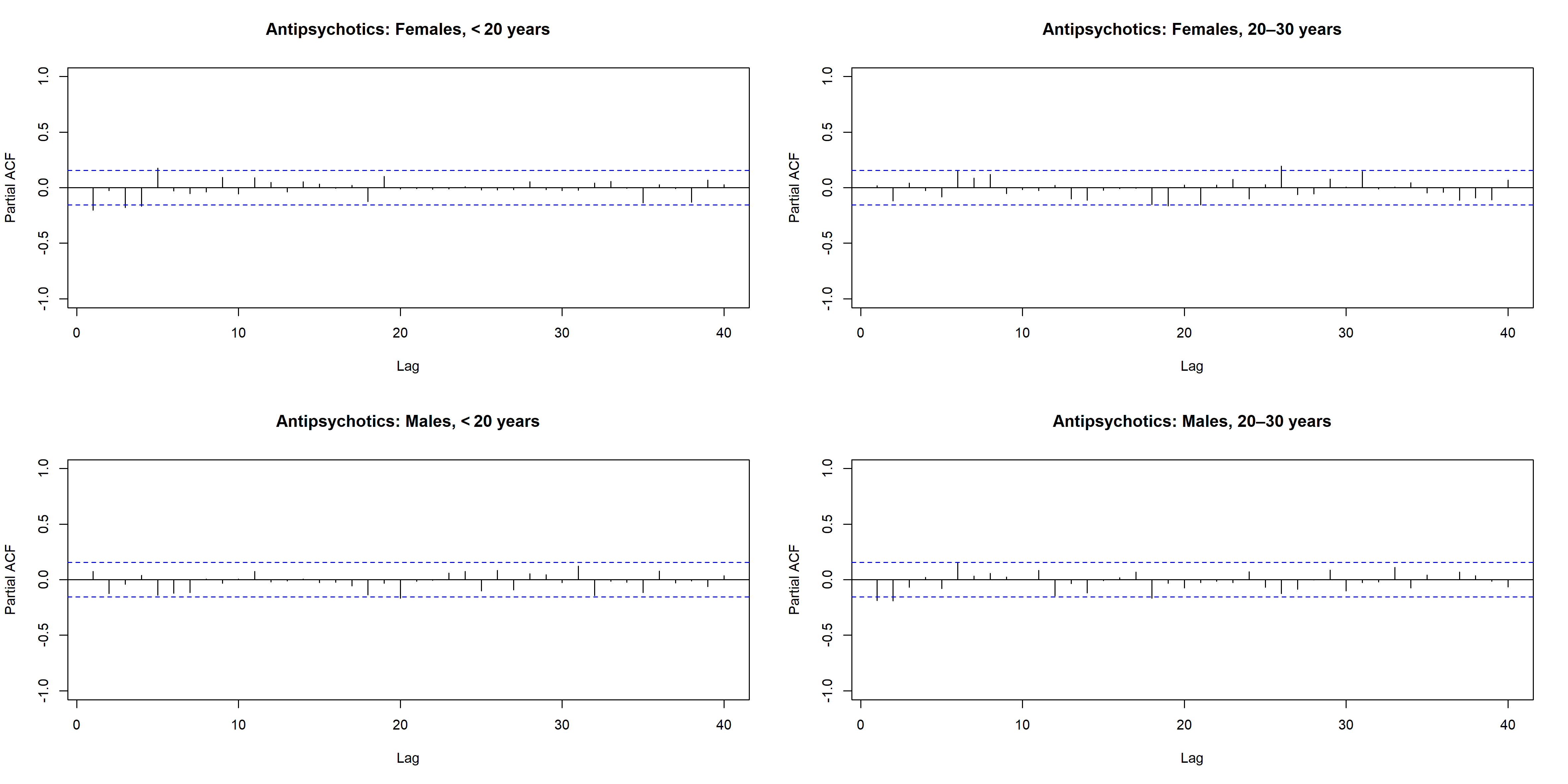

Supplement: Supplementary file 4 [file DataSheet2.ZIP › diagnostic_plots/Outpatient_Stratified_PACF_Antipsychotics.tiff]

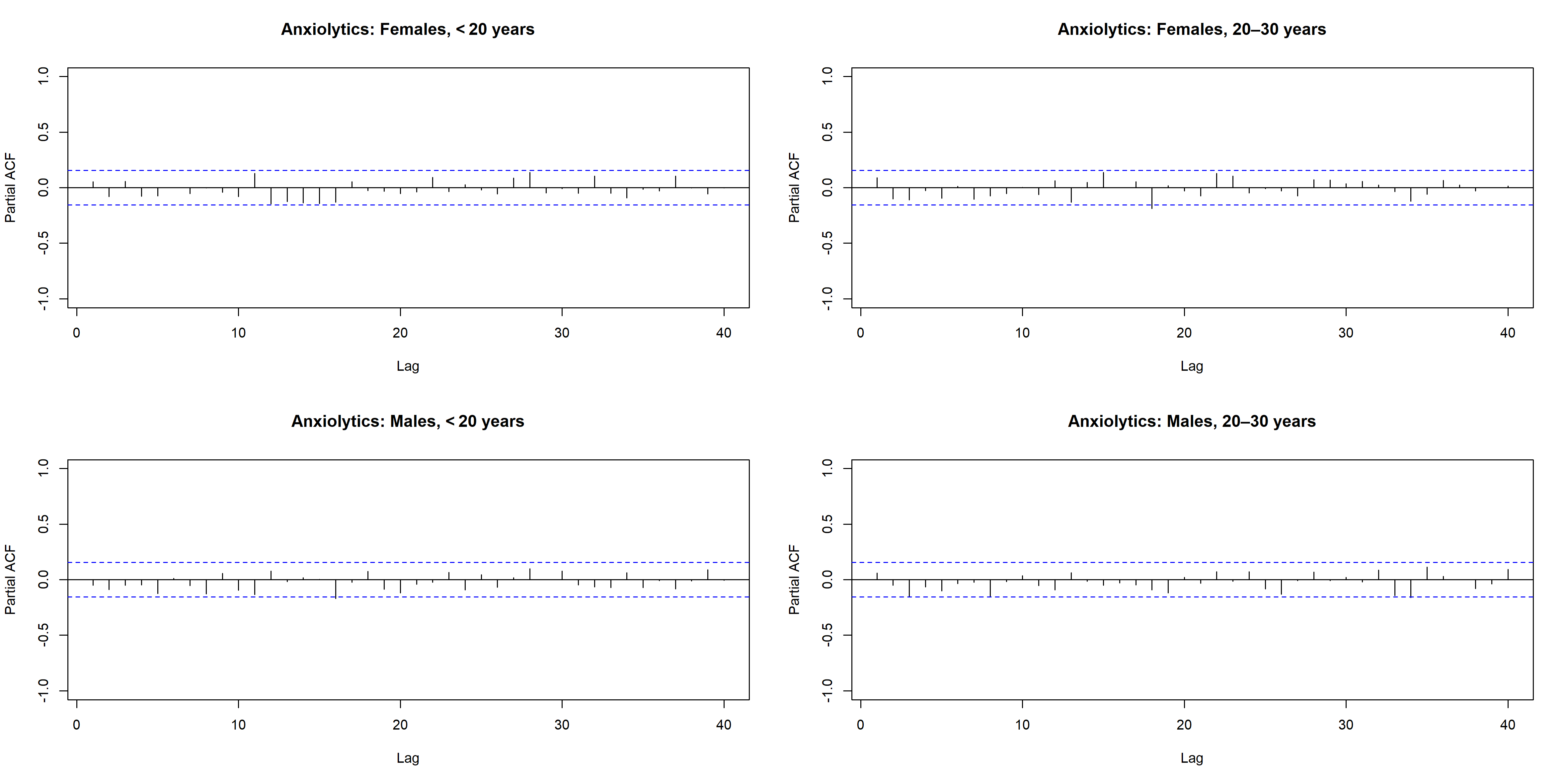

Supplement: Supplementary file 4 [file DataSheet2.ZIP › diagnostic_plots/Outpatient_Stratified_PACF_Anxiolytics.tiff]

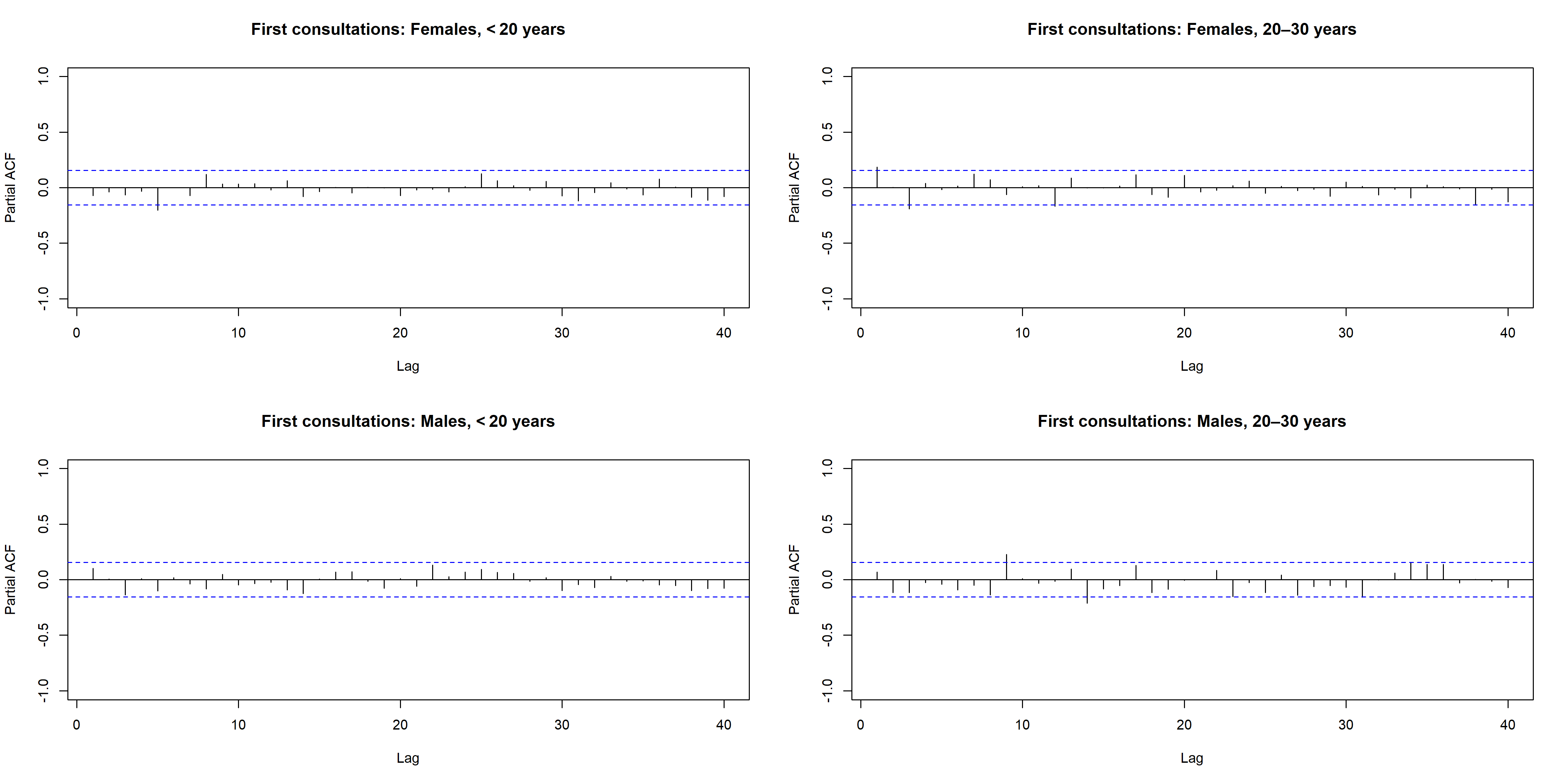

Supplement: Supplementary file 4 [file DataSheet2.ZIP › diagnostic_plots/Outpatient_Stratified_PACF_First consultations.tiff]

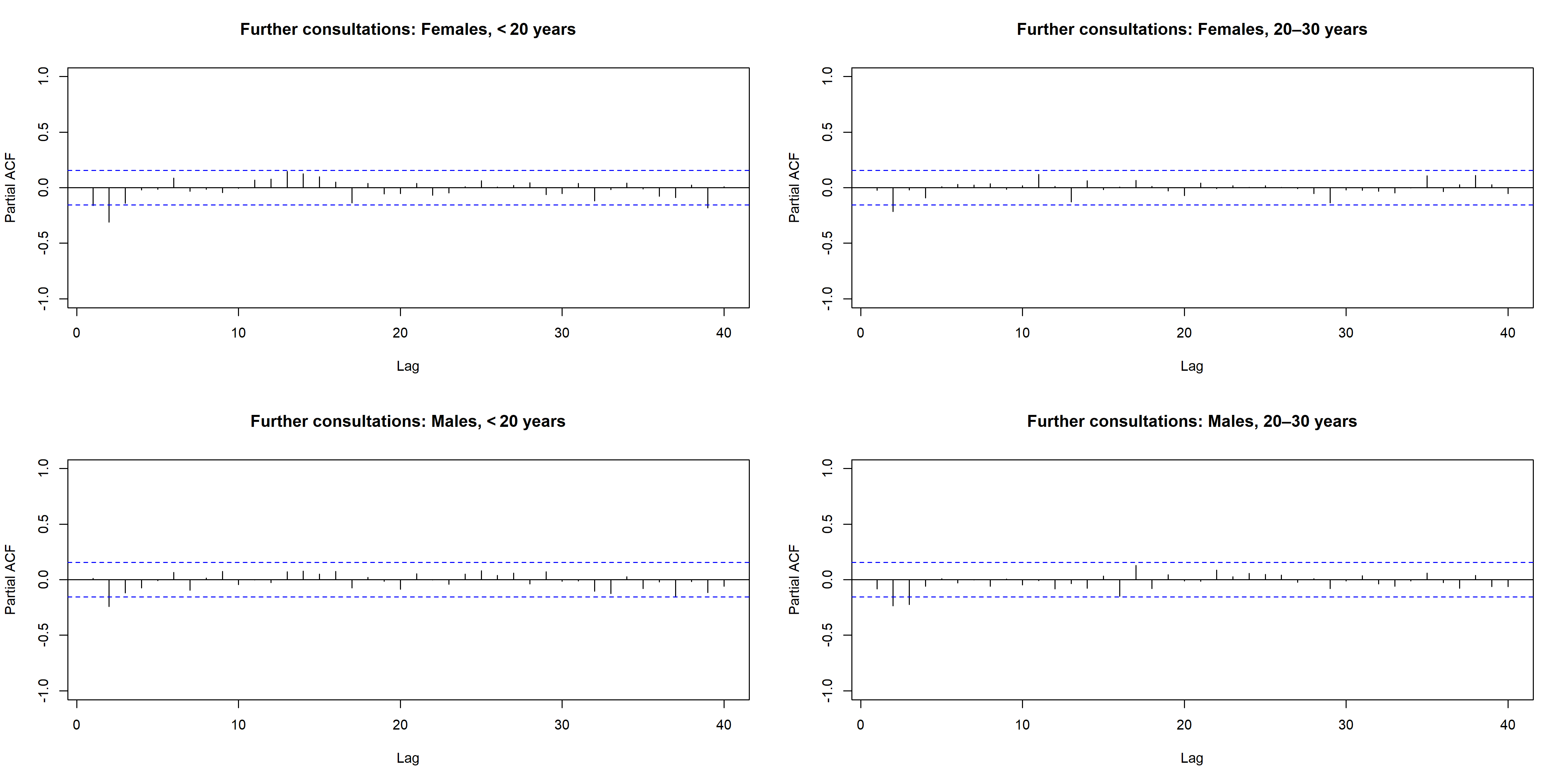

Supplement: Supplementary file 4 [file DataSheet2.ZIP › diagnostic_plots/Outpatient_Stratified_PACF_Further consultations.tiff]

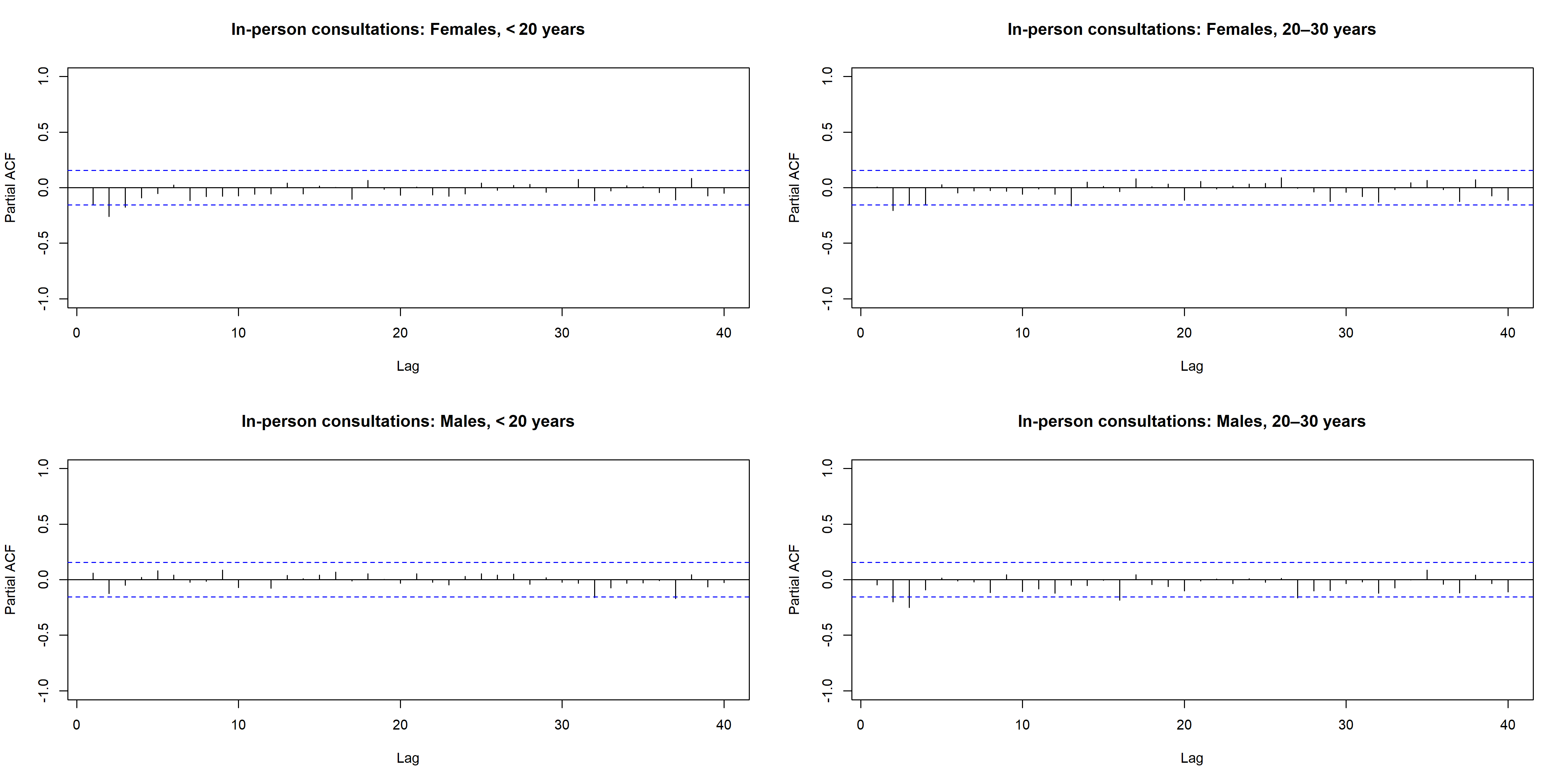

Supplement: Supplementary file 4 [file DataSheet2.ZIP › diagnostic_plots/Outpatient_Stratified_PACF_In-person consultations.tiff]

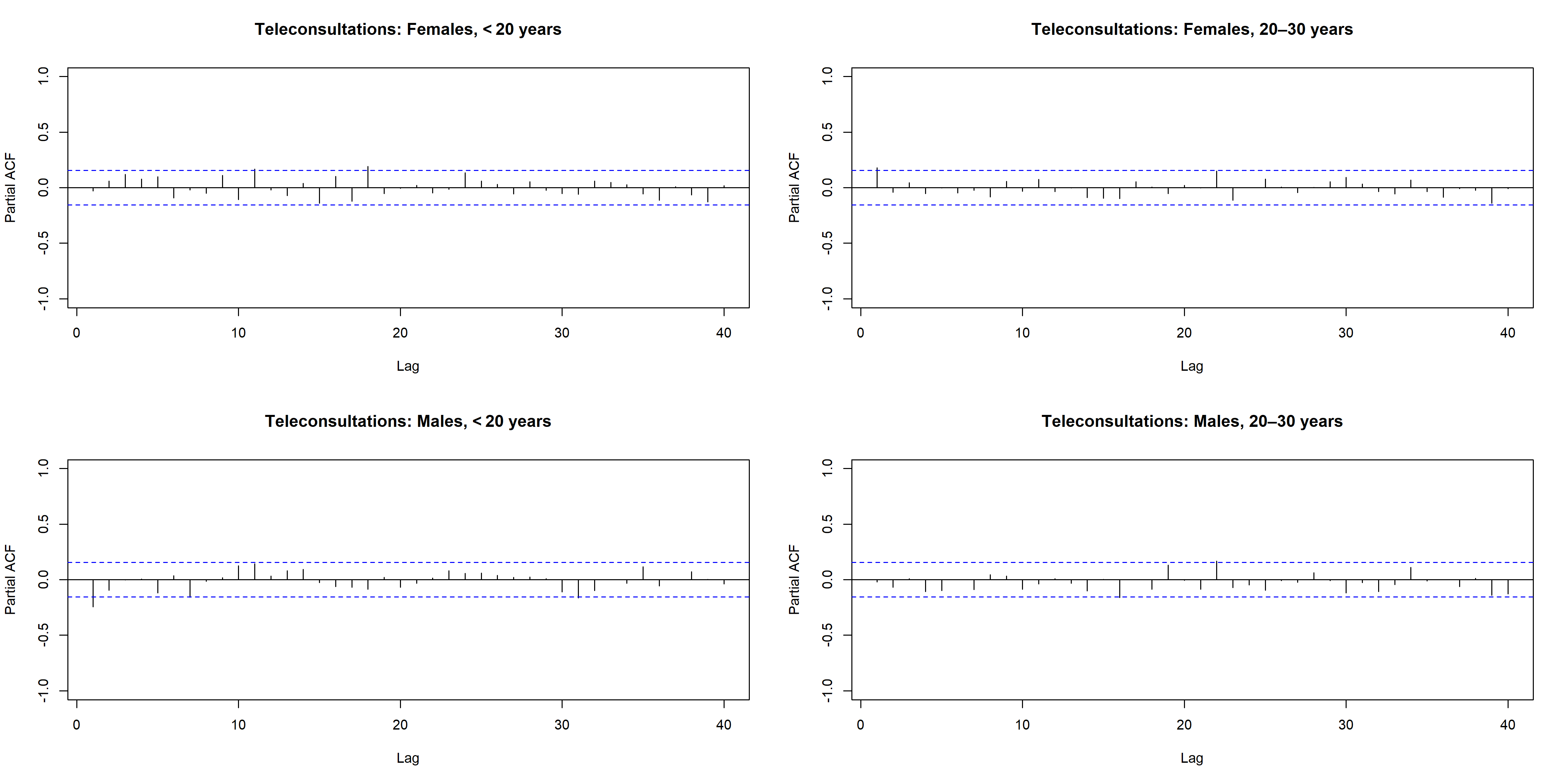

Supplement: Supplementary file 4 [file DataSheet2.ZIP › diagnostic_plots/Outpatient_Stratified_PACF_Teleconsultations.tiff]

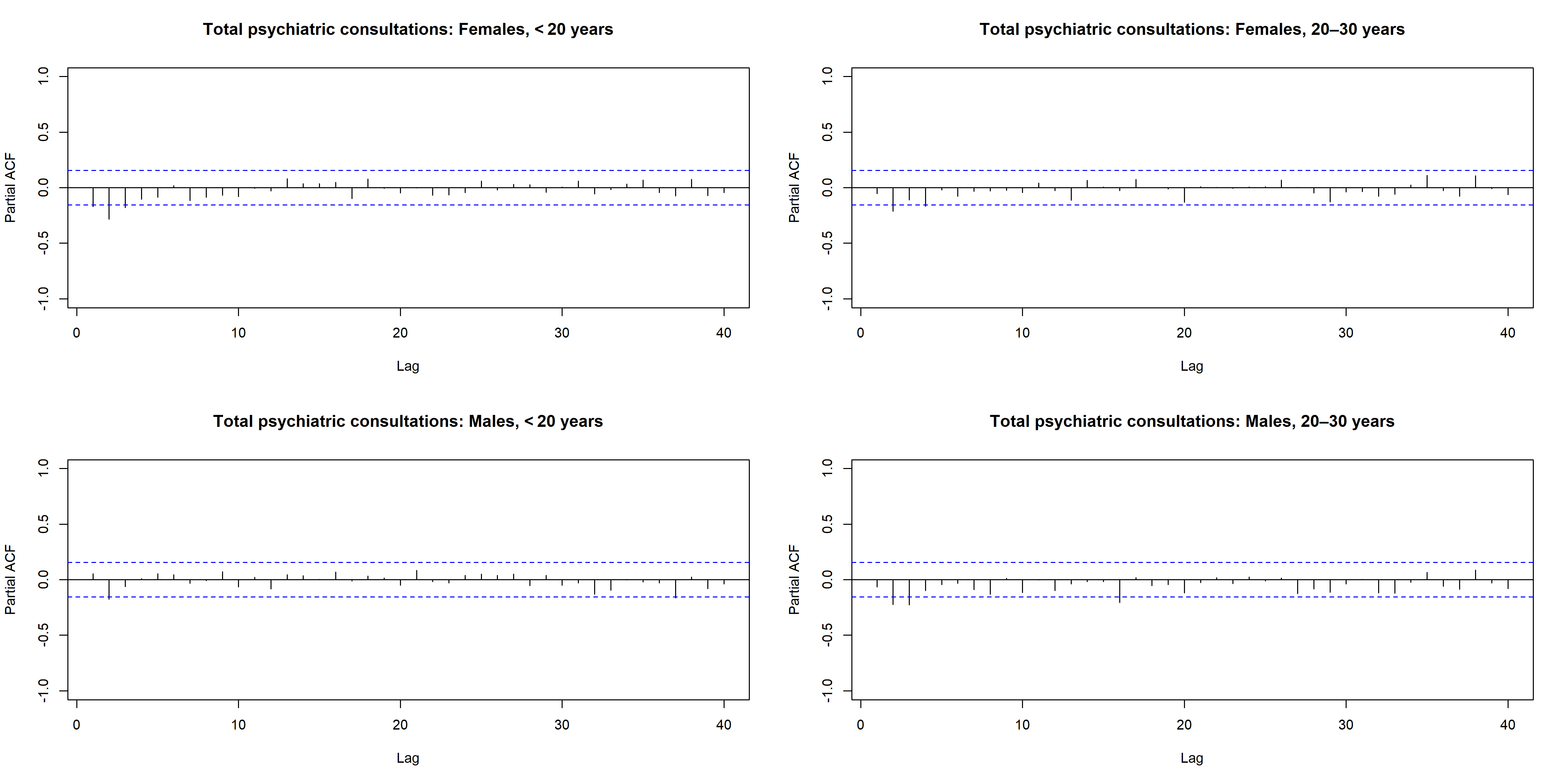

Supplement: Supplementary file 4 [file DataSheet2.ZIP › diagnostic_plots/Outpatient_Stratified_PACF_Total psychiatric consultations.tiff]

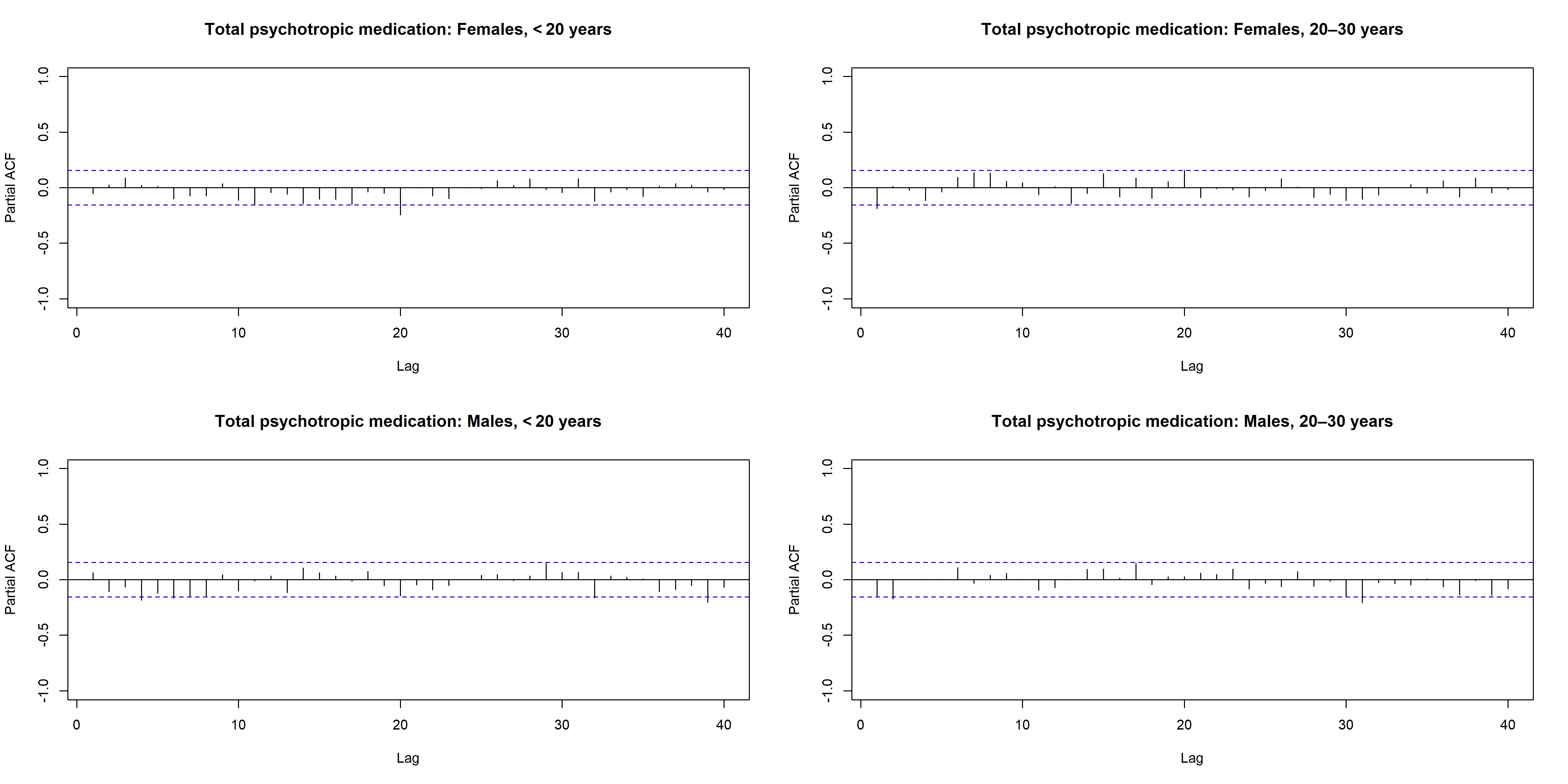

Supplement: Supplementary file 4 [file DataSheet2.ZIP › diagnostic_plots/Outpatient_Stratified_PACF_Total psychotropic medication.tiff]

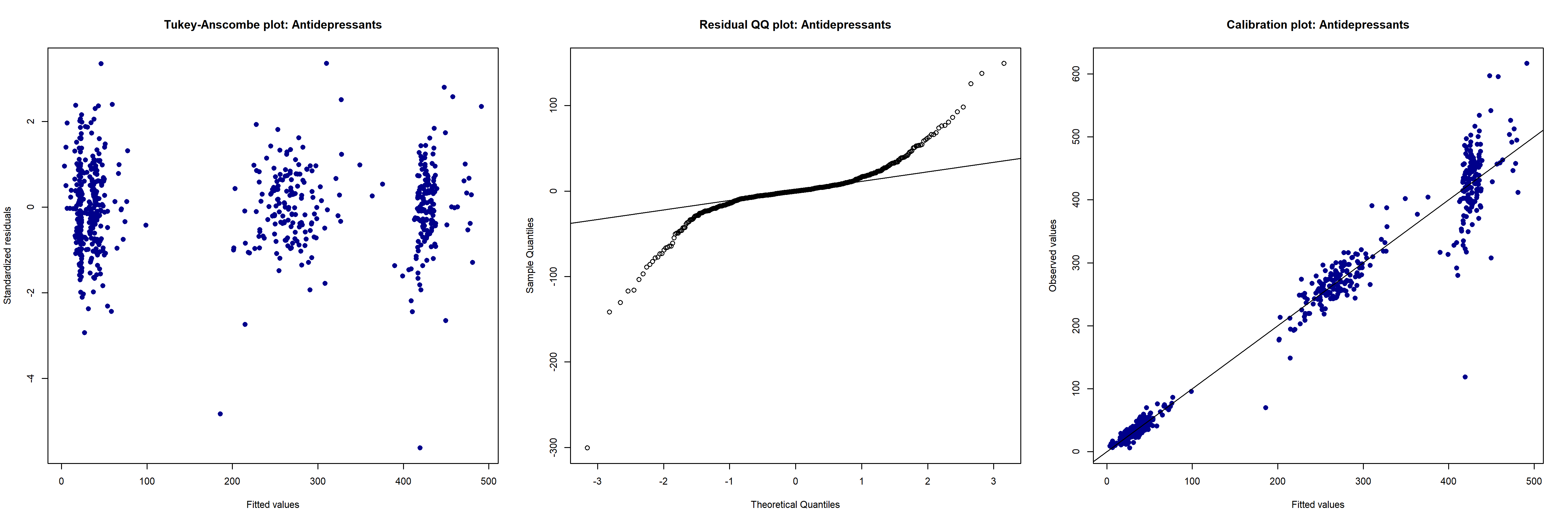

Supplement: Supplementary file 4 [file DataSheet2.ZIP › diagnostic_plots/Outpatient_Stratified_Residuals_Antidepressants.tiff]

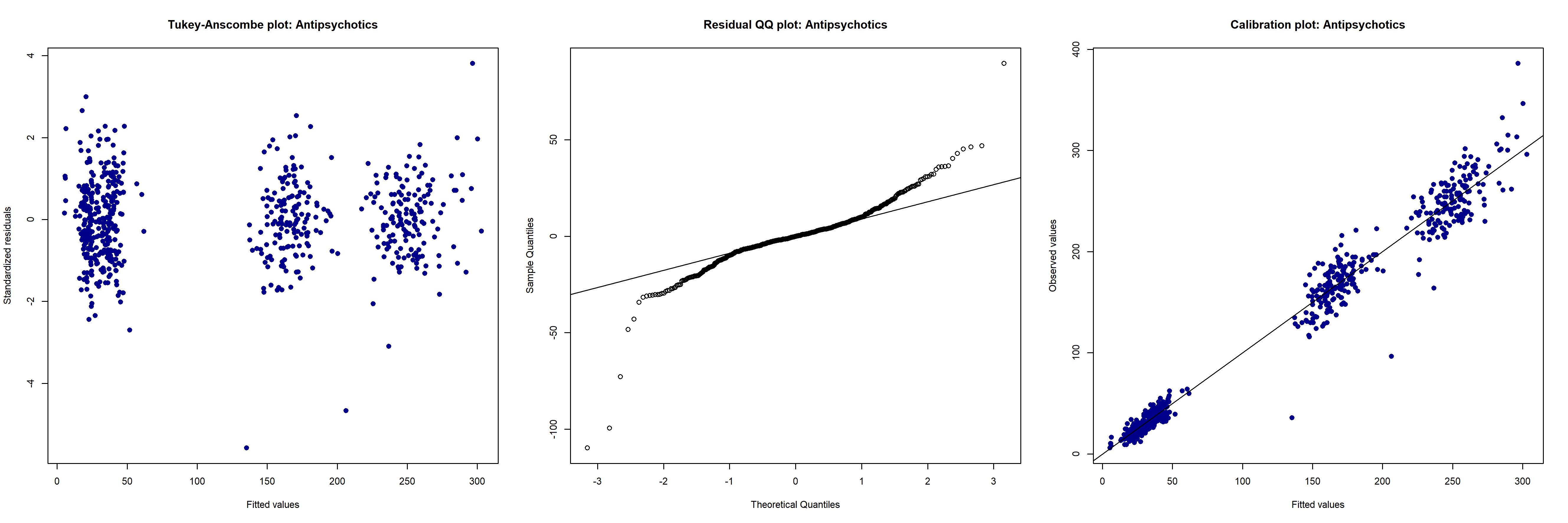

Supplement: Supplementary file 4 [file DataSheet2.ZIP › diagnostic_plots/Outpatient_Stratified_Residuals_Antipsychotics.tiff]

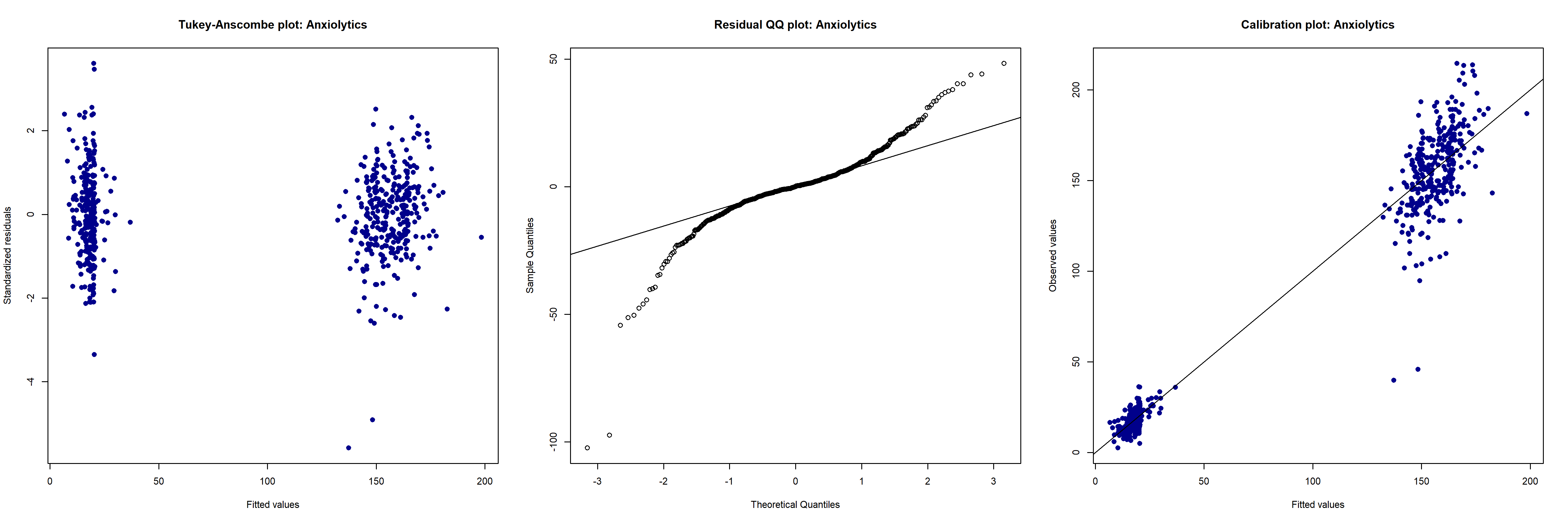

Supplement: Supplementary file 4 [file DataSheet2.ZIP › diagnostic_plots/Outpatient_Stratified_Residuals_Anxiolytics.tiff]

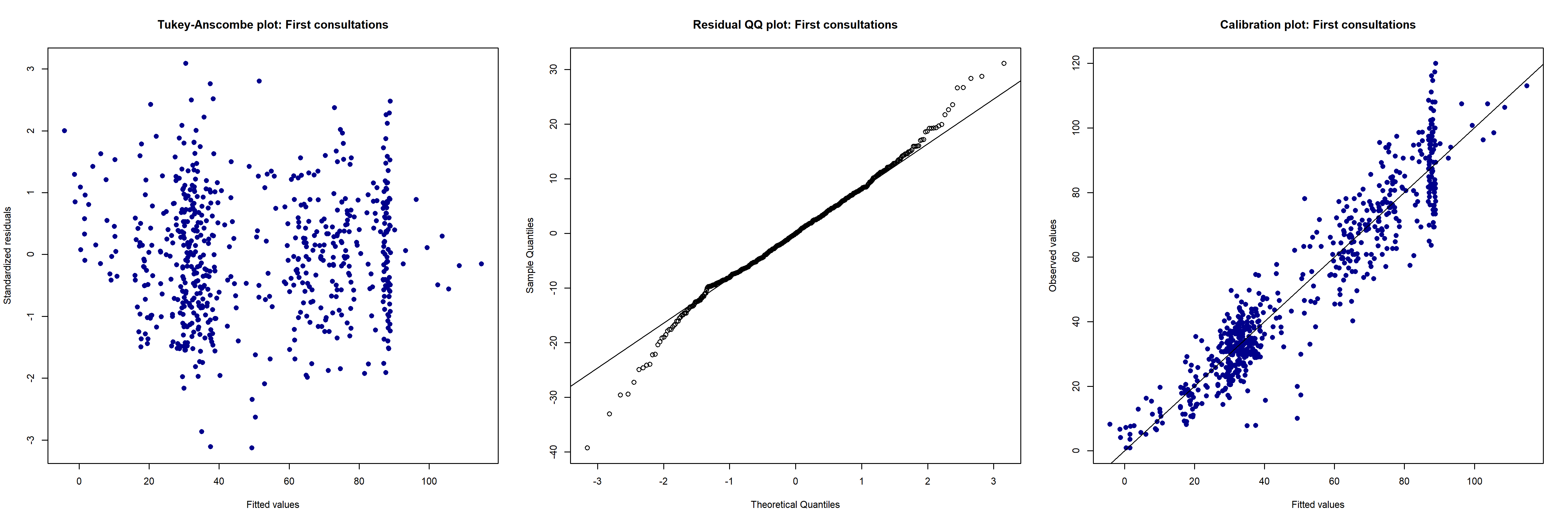

Supplement: Supplementary file 4 [file DataSheet2.ZIP › diagnostic_plots/Outpatient_Stratified_Residuals_First consultations.tiff]

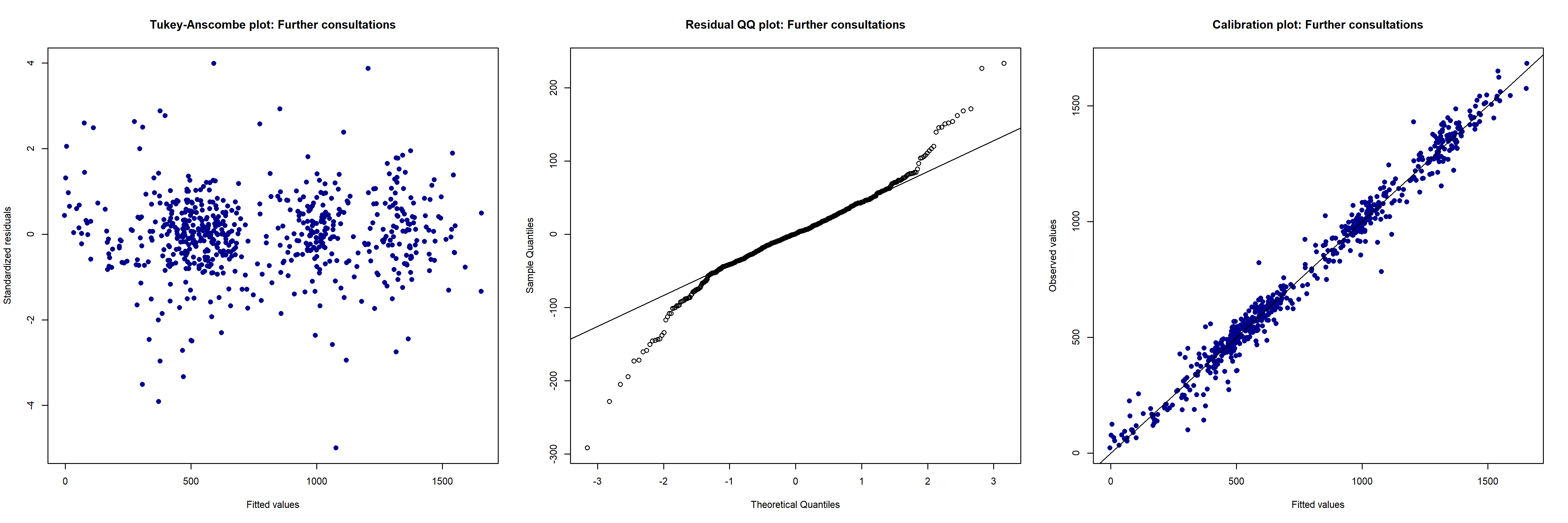

Supplement: Supplementary file 4 [file DataSheet2.ZIP › diagnostic_plots/Outpatient_Stratified_Residuals_Further consultations.tiff]

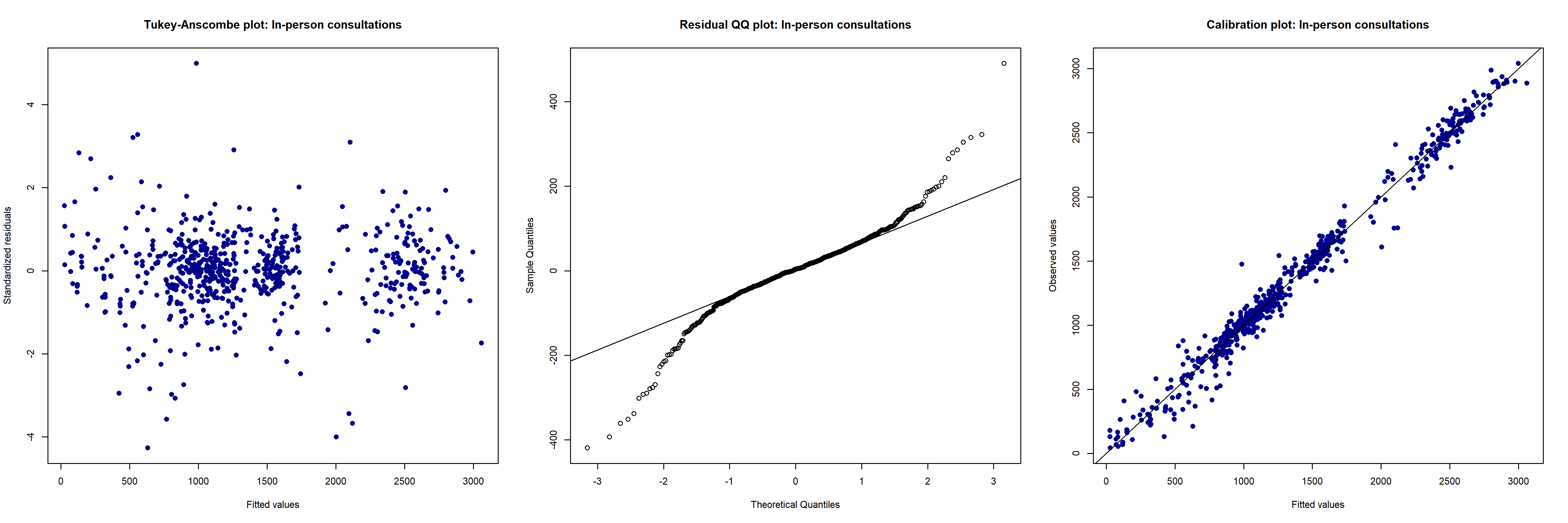

Supplement: Supplementary file 4 [file DataSheet2.ZIP › diagnostic_plots/Outpatient_Stratified_Residuals_In-person consultations.tiff]

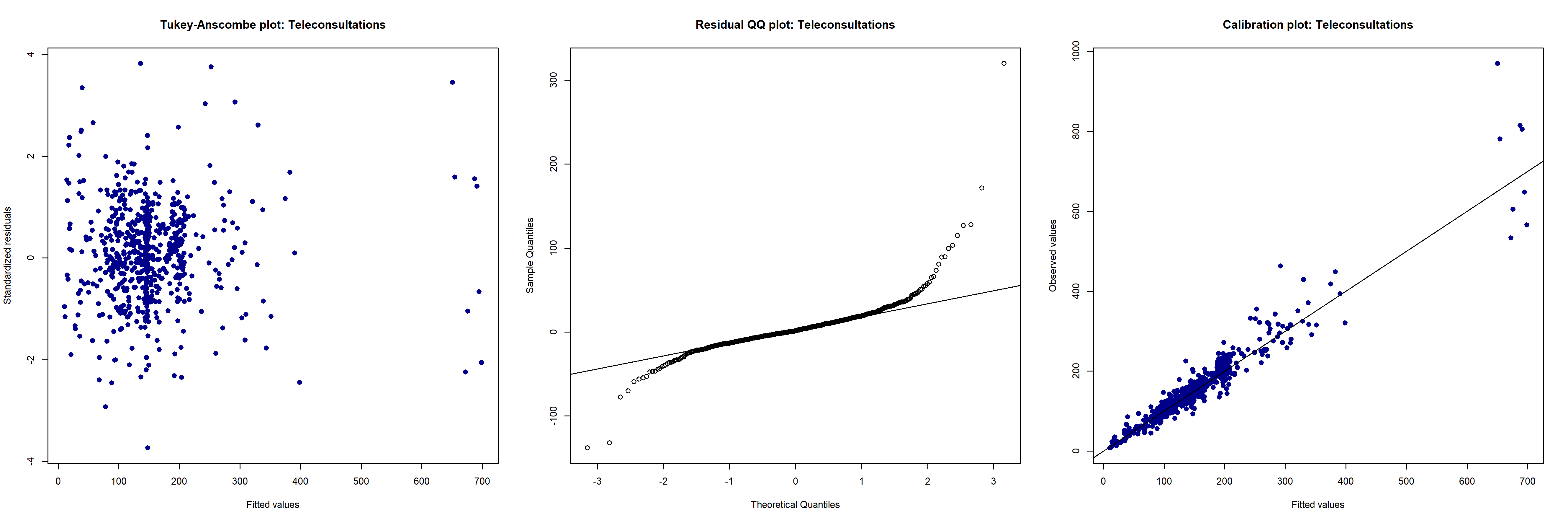

Supplement: Supplementary file 4 [file DataSheet2.ZIP › diagnostic_plots/Outpatient_Stratified_Residuals_Teleconsultations.tiff]

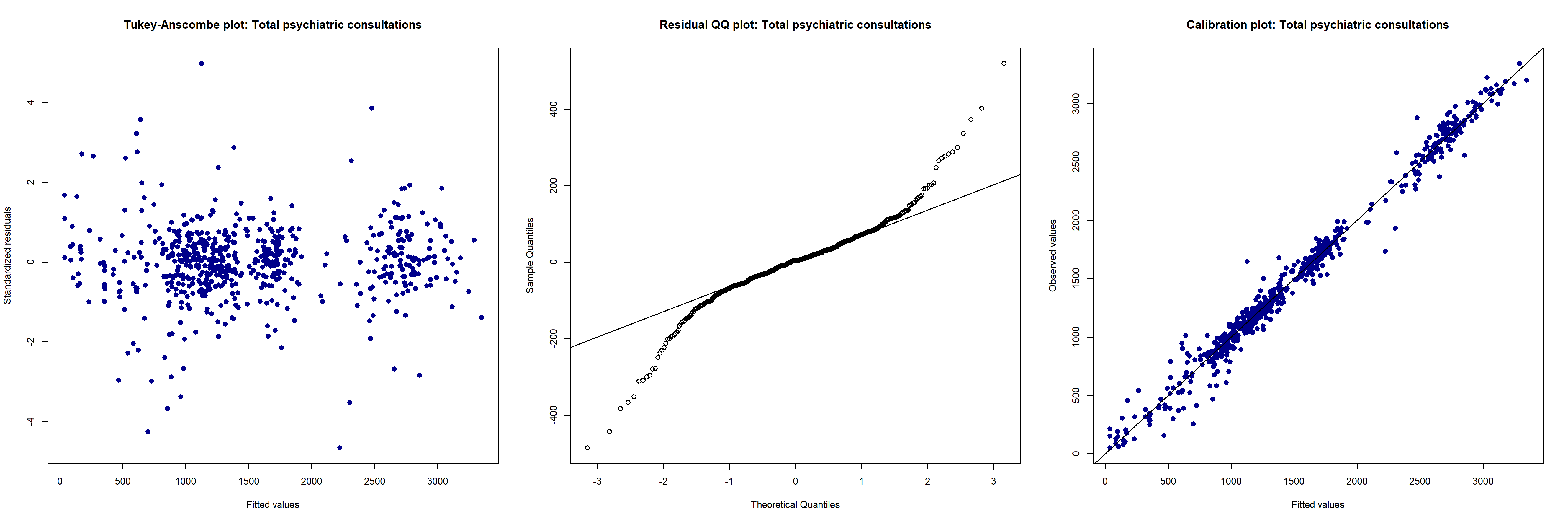

Supplement: Supplementary file 4 [file DataSheet2.ZIP › diagnostic_plots/Outpatient_Stratified_Residuals_Total psychiatric consultations.tiff]

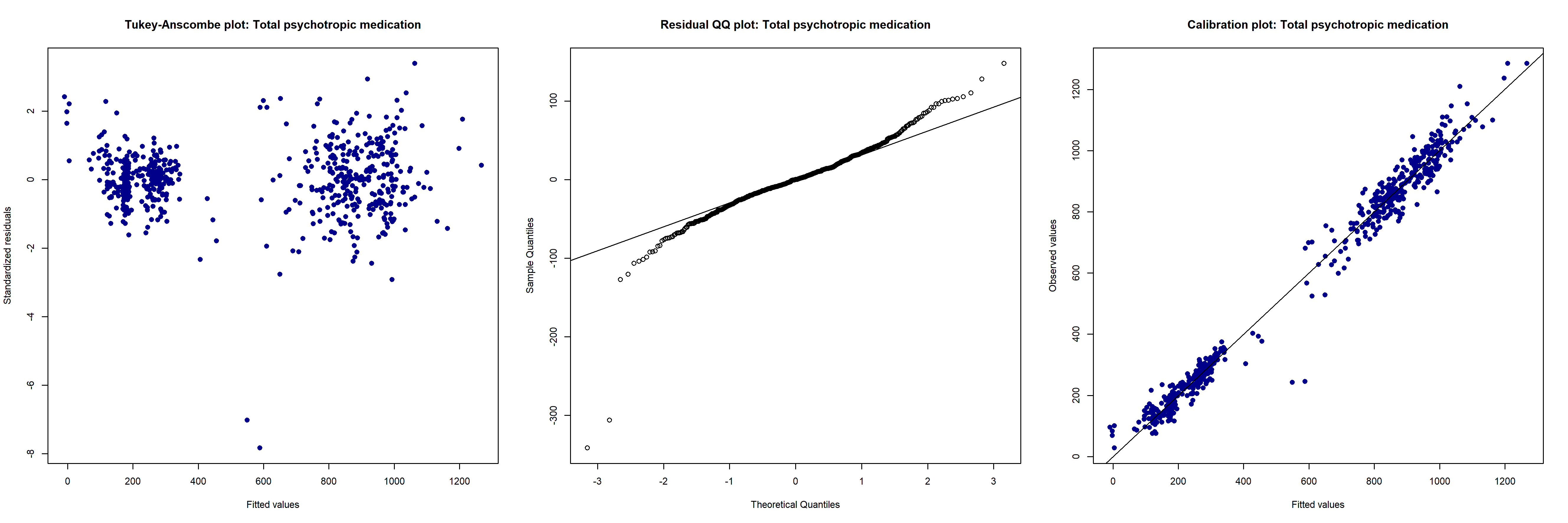

Supplement: Supplementary file 4 [file DataSheet2.ZIP › diagnostic_plots/Outpatient_Stratified_Residuals_Total psychotropic medication.tiff]
